# Seawater transfer alters the intestinal microbiota profiles of Atlantic salmon (*Salmo salar* L.)

**DOI:** 10.1038/s41598-017-13249-8

**Published:** 2017-10-24

**Authors:** Carola E. Dehler, Christopher J. Secombes, Samuel A. M. Martin

**Affiliations:** Institute of Biological and Environmental Sciences, University of Aberdeen, Tillydrone Avenue, Aberdeen, AB24 2TZ UK

## Abstract

Atlantic salmon undergo dramatic physiological changes as they migrate from freshwater to the marine environment. Osmoregulatory adaptation is the most crucial change, necessitating functional adaptations of the gills, kidney and intestine. Additionally, novel pathogens, microbes and dietary items are encountered in the saltwater environment, which suggests major changes in the intestinal microbiota following movement to saltwater. Here we compared the intestinal microbiota harboured in the distal digesta of Atlantic salmon freshwater fish (FW) kept in a commercial Scottish freshwater hatchery with that of their full-siblings after seawater acclimatisation (SW) by a 16S rRNA (V3-V4) high-throughput sequencing approach. Alpha- and beta-diversity were found significantly higher in FW compared to SW, both in terms of richness and diversity. Metastats analysis identified a higher number of Operational Taxonomic Units (OTUs) unique to FW compared to SW, with an additional 238 OTUs found at significantly different abundance. A core microbiota of 19 OTUs was identified in 100% of all fish, which indicates that certain microbes are maintained to fulfil minimal functions within the gut. Furthermore we show that the uniqueness of the respective microbial profiles can be correlated with significant differences in KEGG pathways including lipid and amino acid metabolism.

## Introduction

As part of their natural life history, Atlantic salmon transform from freshwater dwelling parr to seawater adapted smolt when the juvenile salmon migrate to the marine environment. The development to a smolt, termed smoltification, is under environmental cues, especially photoperiod and involves major changes in the fish’s physiology, behaviour and morphology^1,2^.

The salmonid intestine is a complex multifunctional organ, crucial for many functions including absorption of nutrients, immune surveillance^3^ and osmoregulation especially during the transition from freshwater to seawater by balancing uptake and maintenance of electrolytes and water^4,5^. In seawater, fish drink continuously to counteract water loss to the hyperosmotic environment, thereby increasing the luminal alkalinity to that of surrounding medium at a pH above 7.0 to around 8.0^6–8^. Furthermore, whilst the nutritional roles are kept constant, the osmoregulatory functions change, which in turn may further alter physiochemical compositions in the intestine^6^.

Intestinal microbes have key roles in nutrient absorption and protection from pathogens amongst others^9^. The digestive secreta, varies in composition along the gastro-intestinal (GI) tract and provides a substrate for commensal microbes and antimicrobial components protecting the host from potentially harmful bacteria^10^. The colonisation of the intestine by specific microbes may be correlated with physiochemical factors including gastric acidity, digestive enzymes and bile salts^4^. Hence, changes in the host internal pH and osmolality could have important implications for the resident and transient microbes^7^. External microbial changes of the salmon skin microbiota have been reported previously, showing major differences in microbial richness and diversity^11^. Adaptive microbial community shifts may therefore be apparent during the habitat shift of the host caused by the necessary physiological changes required for host survival^12^.

The main uptake of microbes in fish is from the surrounding water and diet and as such it could be predicted that the intestinal microbiota would differ between freshwater and seawater fish, reflecting different environmental composition^13,14^. When examined together, the microbiota of fish and their environment cluster together, but a number of bacterial species are uniquely found in the fish intestine and absent in the water column^15–18^. This suggests a much more complex mechanism for microbial colonisation with a stochastic environmental component (i.e. certain microbes may be locally distinct/abundant and by chance fill the niche within the host as long as it is beneficial for the host) and a deterministic host physiology component (i.e. host physiology and immune system actively select certain microbes necessary to fulfil minimum functionality of the gut ecosystem)^7,17,18^.

In this study we have compared the intestinal microbiota profiles of the anadromous Atlantic salmon in freshwater fish and seawater dwelling fish to deduce the “normal” microbial profile at each life cycle stage within a commercial aquaculture setting. Furthermore we explored if differences in microbial profiles relate to differences in predicted metagenomics pathways and hence changes in functional activity of the microbiome.

We focussed our investigation on the digesta extracted from the distal intestine, as it has long been identified as the most diverse community within the gastro intestinal (GI) tract^4^. A deep sequencing approach was applied to the extracted DNA to amplify the variable regions V3 and V4 of the common marker gene 16S rRNA, which has been suggested as preferred method to confidently assign taxonomy to the sequences^19^.

The results from this study will contribute to a better understanding of the complex metamorphosis Atlantic salmon undergo during their life history, and the intrinsic interplay between a host and its commensal microbial communities.

## Results

### Sequencing overview and microbiota characterisation

Of the 40 samples sequenced, 33 were maintained for analysis (FW n = 15, SW n = 18), with an average of 77,380 ± 6,327 quality controlled reads (of 92,667 ± 7,231 raw reads). This allowed for rarefaction and even sampling of 5,348 sequences per sample (average sequencing depth 13,498 ± 556 per individual sample) after removal of singletons, *Cyanobacteria* spp. and OTUs mapping to “Other Bacteria” (OB) and “Unclassified Bacteria” (UB). *Cyanobacteria* were removed as known contaminants (planktonic photosynthetic bacteria) and made up 28.4% of the quality controlled reads. OB were found significantly more in FW reads (57 ± 3.3%) compared to SW reads (14 ± 1.6%) (2-tailed t-test, *P* < 0.001), which is consistent with the findings of the alpha and beta diversity analysis performed on the cropped dataset. The removal of OB from further analysis allowed for the focus on differences of classified bacteria, which would have been otherwise masked by the large amount of OB OTUs present. Together OB and UB made up 61.9% of all cleaned-up sequences (data not shown) and were not used in subsequent analysis. Sequences for all OTUs detected are available in supplementary file 1. All raw sequences were submitted to NCBI under Bioproject PRJNA412587, SRA Archive SRP119216.

### Core microbiota

A core microbiota was detected for all samples, regardless of FW or SW habitat (Table 1). Nineteen OTUs were found to be shared overall, with accessory cores in the respective life cycle stage groups (FW = 12 OTUs, SW = 21 OTUs).

**Table 1:** Number of Operational Taxonomic Units (OTUs) in the gut microbiota of Atlantic salmon shared between (overall core) and within the freshwater and seawater groups (accessory freshwater pre-smolt core, accessory seawater post-smolt core).

Phylum	Genus	overall core	accessory FM core	accessory SW core
*Actinobacteria*	*Propionibacterium*		1	
*Bacteroidetes*	*Prevotella*			1
	*Bacillus*		1	1
	*Brochothrix*			1
	*Clostridium*		1	
	*Geobacillus*			1
	*Lactobacillus*	5	2	4
*Firmicutes*	*Lactococcus*	1		1
	*Leuconostoc*		1	
	*Megasphaera*			1
	Other *Ruminococcaceae*		1	
	*Staphylococcus*	1		
	*Streptococcus*	2	1	
	*Veillonella*		1	
	*Weissella*	1		
*Fusobacteria*	Other *Fusobacteriaceae*	1		
	*Arcobacter*			1
	*Brevundimonas*		1	
	*Escherichia/Shigella*	1		
	*Mesorhizobium*		1	
	Other *Enterobacteriaceae*	1		3
*Proteobacteria*	Other *Oxalobacteraceae*			2
	*Paracoccus*			1
	*Photobacterium*	1		
	*Pseudomonas*	2		3
	*Rhizobium*	1		
	*Sphingomonas*	1	1	
	*Stenotrophomonas*			1
*Tenericutes*	*Mycoplasma*	1		

The common OTUs mapped predominantly to lactic acid bacteria (LAB, phylum *Firmicutes*, n = 10), well known intestinal microbes including *Escherichia/Shigella* (phylum *Proteobacteria*) and *Mycoplasma* spp. (phylum *Tenericutes*), a genus that has been consistently identified in studies of Atlantic salmon intestines and appears especially important for SW fish in this study.

Notably, all FW samples shared a *Propionibacterium* OTU (phylum *Actinobacteria*), which is in agreement with the higher diversity of this phyla for this life stage group (Supplementary Figure S2, Supplementary Table S5). Furthermore, the *Firmicutes* class *Clostridiales* was represented by 3 OTUs mapping to well-known intestinal microbe genera *Clostridium*, *Ruminococcaceae* and *Veillonella*. We found that unique LAB OTUs mapping to the genera *Lactobacillus*, *Lactococcus*, *Leuconostoc* and *Streptococcus* are maintained by FW and SW fish. This is consistent with the observation with the high number of unique LAB found in one group but not the other, with significantly different abundances, as suggested by Metastats analysis (Supplementary Tables S6 and S7).

### Microbiota differences between freshwater and seawater life cycle stages

In total 2,864 OTUs were retained for analysis after removal of *Cyanobacteria* spp., OB, UB, singletons and chimeric sequences. These OTUs mapped to 19 phyla in FW fish and 18 in SW fish. Phyla present only in FW samples were *Bacteria_incertae_sedis* (144 sequences), *Chloroflexi* (19 sequences), *Gemmatimonadetes* (357 sequences) and *Synergistetes* (7 sequences). SW unique phyla were identified as *Fibrobacteres*, *Nitrospira* and *Thermatogae*, however, these were at very low relative abundances, with eight, two and twelve sequences identified respectively.

**Figure 1:**
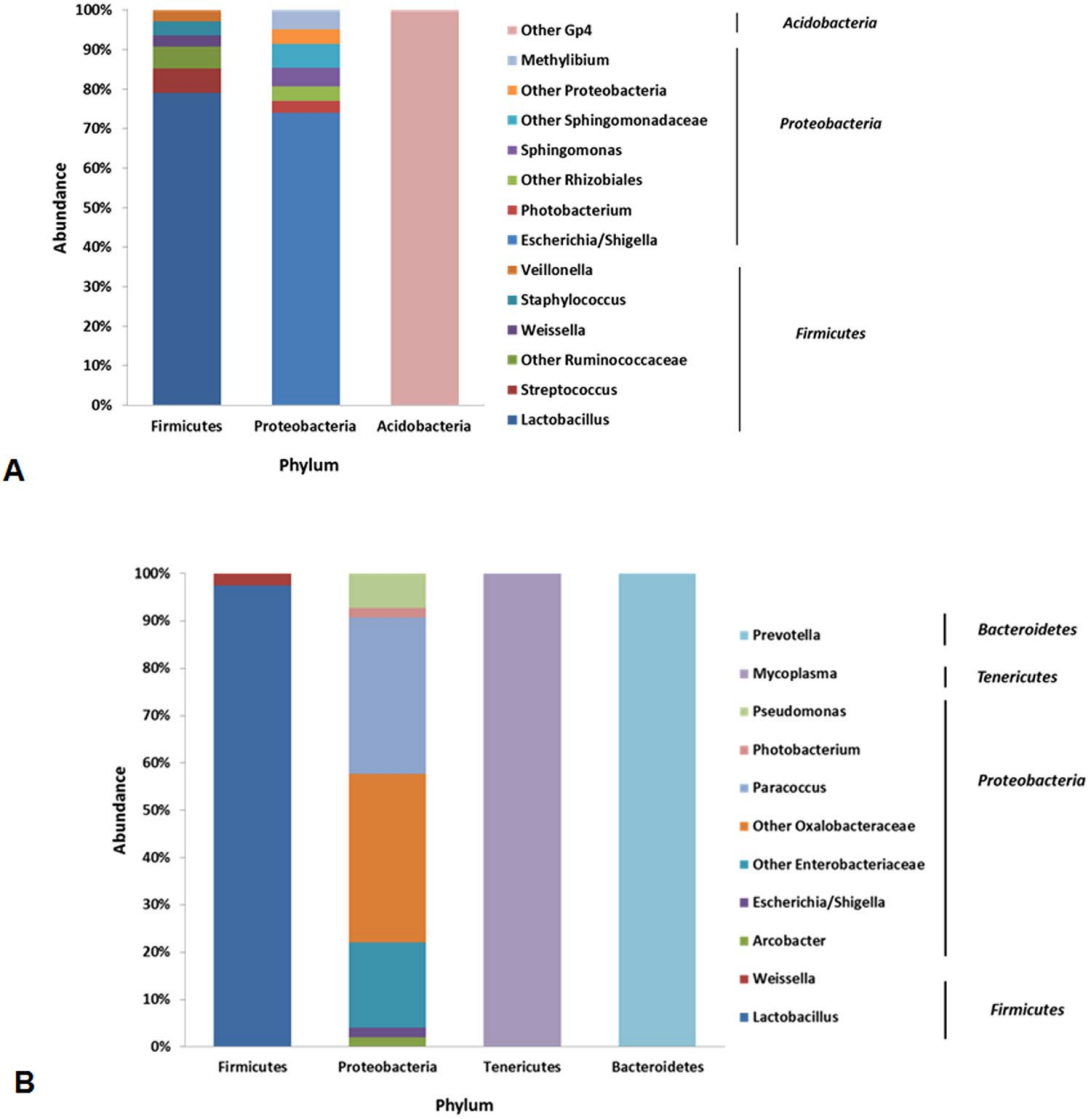
Top 20 most abundant Operational Taxonomic Units (OTUs) detected in Atlantic salmon in either fresh-or saltwater stages. The percent abundance refers to the relative proportion of a top 20 OTU containing genera within its parent phylum displayed on the x-axis. (**A**) Freshwater stage (Pre-smolt), overall abundance distribution of phyla containing the top 20 OTUs: *Firmicutes* 36.8%, *Proteobacteria* 60.8% and *Acidobacteria* 2.4%. (**B**) Seawater stage (Post-smolt), overall abundance distribution of phyla containing the top 20 OTUs: *Firmicutes* 55.6%, *Proteobacteria* 21%, *Tenericutes* 23% and *Bacteroidetes* 0.4%.

Microbial communities of the FW fish contained on average 359 ± 37 OTUs, whereas SW fish harboured an average of 275 ± 24 OTUs. The top 20 OTUs found in FW fish mapped to three phyla (*Firmicutes, Proteobacteria* and *Acidobacteria*) and 14 genera (one *Acidobacteria*, six *Firmicutes* and seven *Proteobacteria*) (Fig. 1A). In SW fish, the top 20 OTUs mapped to four phyla (*Firmicutes*, *Proteobacteria*, *Tenericutes* and *Bacteroidetes*) and eleven genera (one *Bacteroidetes*, two *Firmicutes*, seven *Proteobacteria* and one *Tenericutes*) (Fig. 1B). Interestingly, the top 20 OTUs overall in FW fish only accounted for 12,079 sequences, whereas in the overall SW fish group 31,790 sequences were mapped to the top 20 OTUs, suggesting higher microbial diversity in FW samples. This is supported by the full breakdown of microbial profiles by sample and life cycle group, which can be found in the supplementary material (Supplementary Figures S2 and S3).

The order *Lactobacillales* was targeted for additional analysis due to its importance for fish health and the observed significantly different abundance in FW and SW fish (36.7% and 54.8% of all bacterial orders respectively, *P* = 0.0262). Within this order, five families showed significantly different abundance, with *Lactobacillaceae* more abundant in SW fish, whilst *Streptococcaceae*, *Leuconostocaceae*, *Enterococcaceae* and *Carnobacteriaceae* were more abundant in FW fish. Nine genera were found to be significantly different abundant between FW and SW fish (Supplementary Figure S4). *Lactobacillus* was the dominant genera in SW fish compared to FW fish (95.23% and 52.28% of LAB respectively, *P* < 0.0001). FW fish also maintained a notable proportion of *Streptococcus* (36.44%) and *Weissella* (5.68%), which were significantly more abundant compared to SW fish (1.06% and 2.31%, *P* < 0.0001 and *P* = 0.0086 respectively). Other LAB genera significantly more abundant in FW fish included *Pediococcus, Leuconostoc, Vagococcus, Enterococcus* and *Carnobacterium*, whilst “Other *Enterococcaceae*” were significantly more abundant in SW fish.

### Alpha diversity

**Figure 2:**
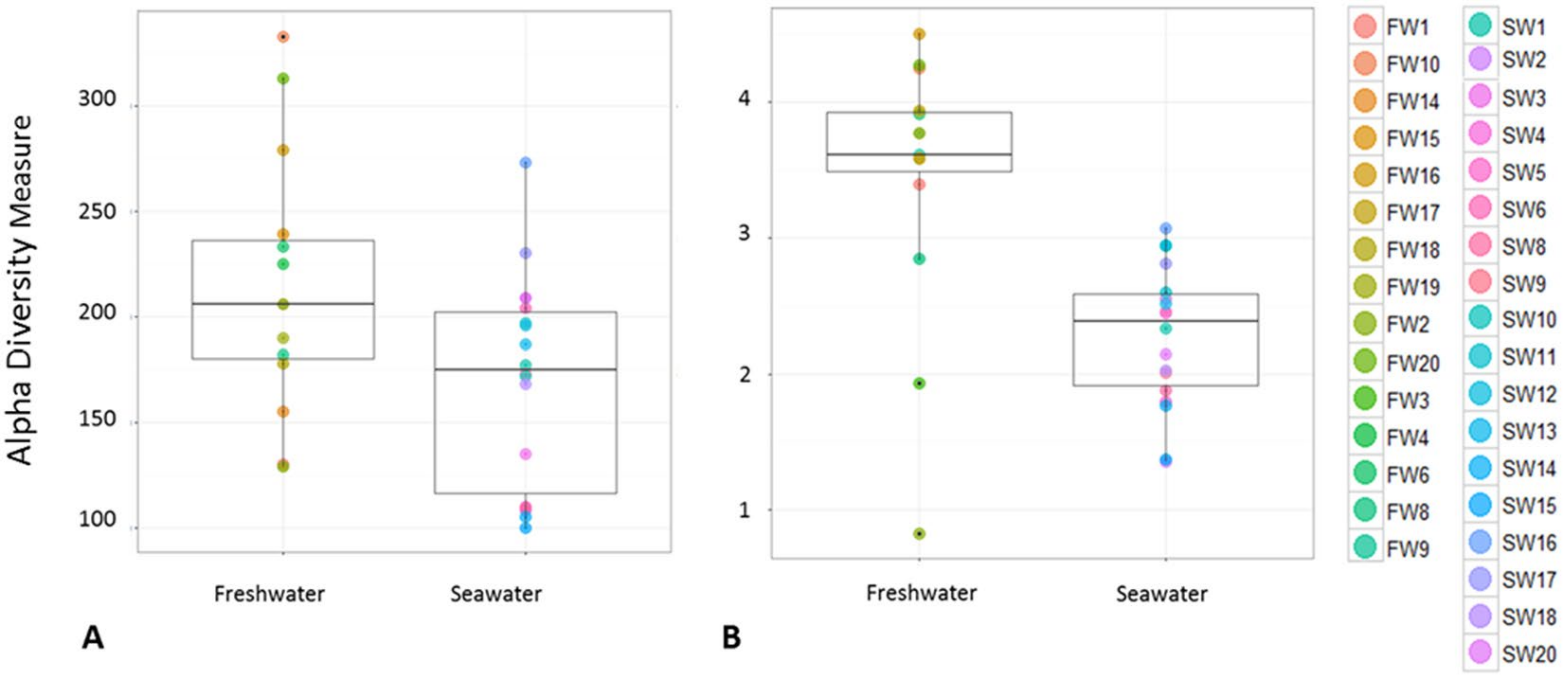
Boxplots showing the distribution of alpha diversity metric values of individual intestinal microbiota profiles detected in Atlantic salmon in fresh- or saltwater. Freshwater (pre-smolt fish), Seawater (post-smolt fish). (**A**) Observed Species metric showing significant differences in species richness between the habitats (*P* < 0.05). (**B**) Shannon index showing significant differences in species diversity between the habitats (*P* < 0.0001).

The common rarefaction level for all samples was identified to be 5,218 sequences. The dataset was accordingly rarefied to allow even sampling depth and comparison between the life stage groups. Species richness and species diversity we calculated by observed OTUs and Shannon diversity indexes respectively (Fig. 2).

By considering only species richness, microbial profiles of FW samples had a significantly larger number of OTUs compared to SW samples (*P* < 0.05). Additionally, Shannon index identified significant differences in the species diversity found in the microbial composition of the two life stages with higher species diversity in FW samples (*P* < 0.0001).

### Beta diversity

**Figure 3:**
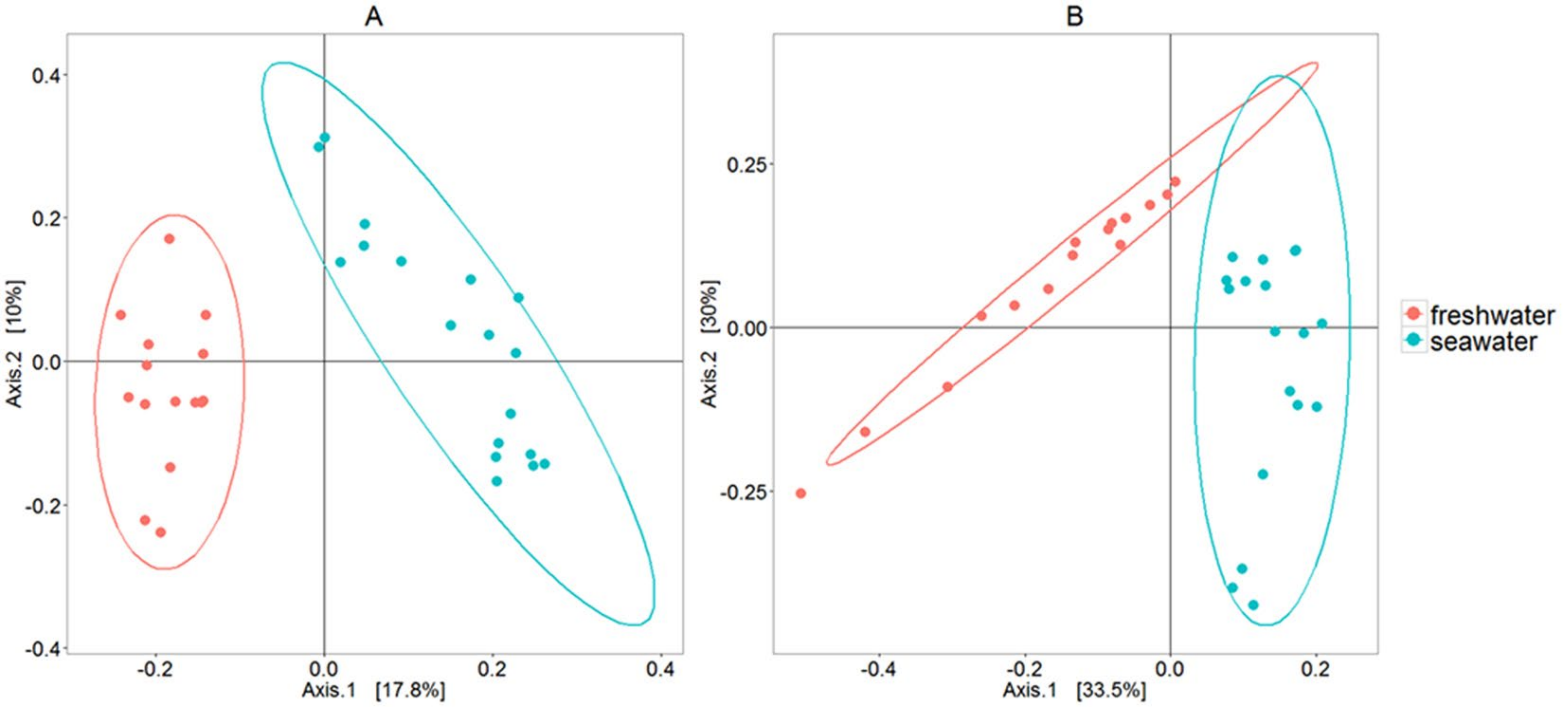
Multiple dimension scale (MDS) plots showing the beta diversity results of intestinal microbial profiles detected in Atlantic salmon (freshwater pre-smolt fish and seawater post-smolt fish). (**A**) Unweighted Unifrac: presence/absence of operational taxonomic units (OTUs) (phylogenetic richness) was found significantly different between the groups (PERMANOVA, Pseudo F-statistic 5.9892, *P* < 0.001, based on 999 permutations). (**B**) Weighted Unifrac: presence/absence/abundance of OTUs (phylogenetic diversity), was also found significantly different (PERMANOVA, Pseudo F-statistic 12.9564, *P* < 0.001, based on 999 permutations).

Phylogenetic richness and diversity were assessed by unweighted and weighted Unifrac respectively, based on a phylogenetic tree of all OTUs identified in the dataset (Lozupone and Knight, 2005; Lozupone *et al*., 2007). Both phylogenetic richness (unweighted Unifrac) and phylogenetic diversity (weighted Unifrac) were significantly higher in FW digesta samples compared to SW digesta samples (PERMANOVA, Pseudo-F = 5.9892, *P* < 0.001 and Pseudo-F = 12.9564, *P* < 0.001 respectively) and is visualised by MDS plots which show a significant relationship by strong clustering of the samples by life stage group (Fig. 3). This strongly supports that changes associated with seawater transfer have an impact on the microbial composition, both in terms of alpha and beta-diversity.

**Figure 4:**
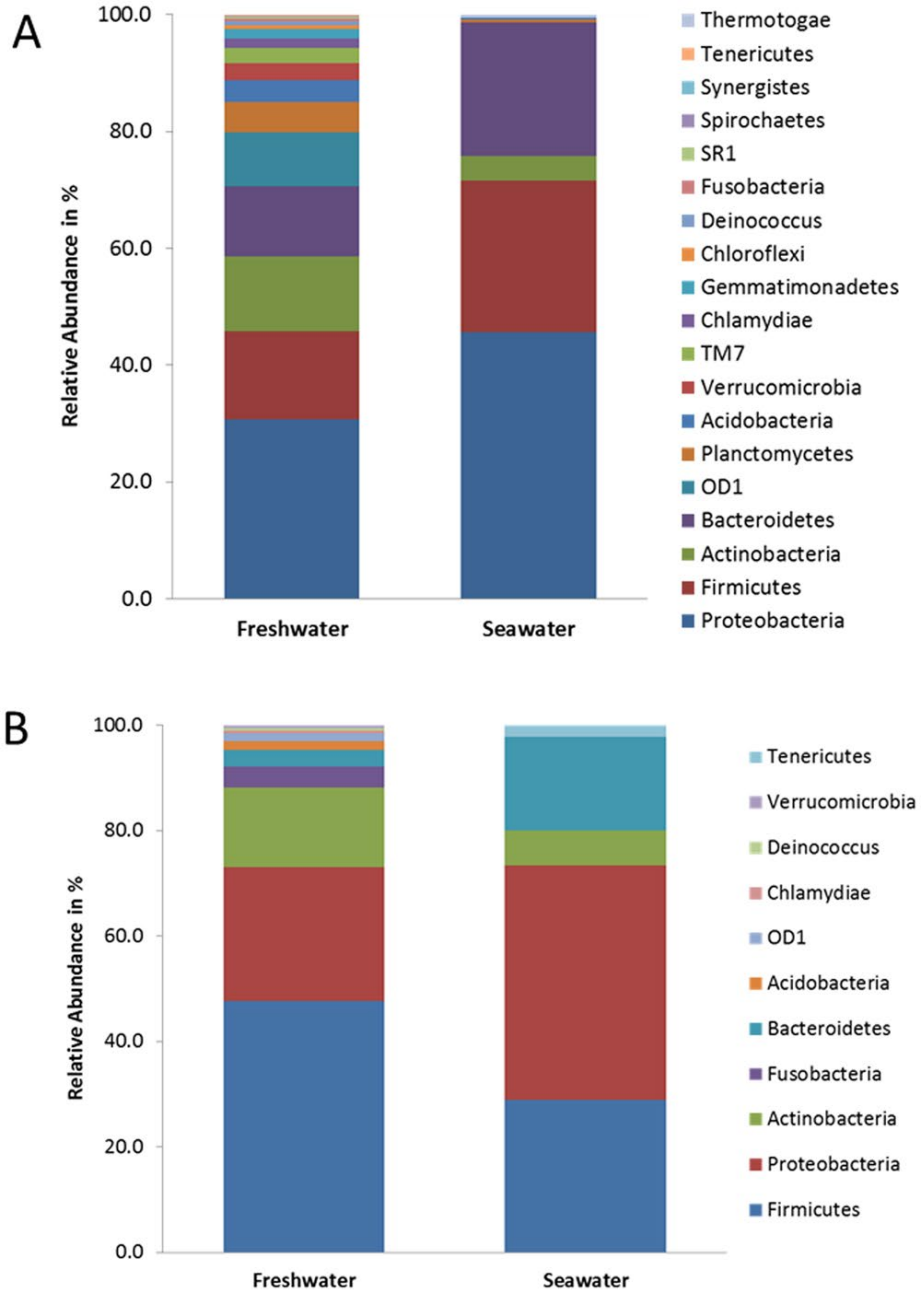
Intestinal microbe Operational Taxonomic Units (OTUs) characteristic for Atlantic salmon (freshwater pre-smolt fish and seawater post-smolt fish) as identified by Metastats analysis and summarised at phylum level. The percent abundance along the y-axis refers to the relative proportions of phyla to which the characteristic OTUs were taxonomically mapped. (**A**) OTUs found uniquely in either the freshwater group (Total = 716 OTUs) or the seawater group (Total = 215 OTUs) summarised by phylum and given as percentage of the absolute OTU number. Comprehensive results can be found in Supplementary Tables S3 and S4. (**B**) OTUs found at significantly higher abundances in either the freshwater group (Total = 193 OTUs) or the seawater group (Total = 45 OTUs) summarised by phylum and given as percentage of the absolute OTU number. Comprehensive results can be found in Supplementary Table S5.

Metastats analysis showed that the FW group contained a higher number of unique OTUs mapping to a wider variety of phyla when compared to the SW group (Fig. 4A). We identified 716 OTUs unique to the FW group (Supplementary Table S3) and 215 OTUs unique to the SW group (Supplementary Table S4). The majority of significantly different abundant OTUs were also found to be at higher levels in the FW group (Fig. 4B). 238 OTUs were found to have a statistically different abundance, with 193 OTUs having a higher abundance in the FW group and 45 OTUs in the SW group (Supplementary Table S5).

### Functional analysis

**Figure 5:**
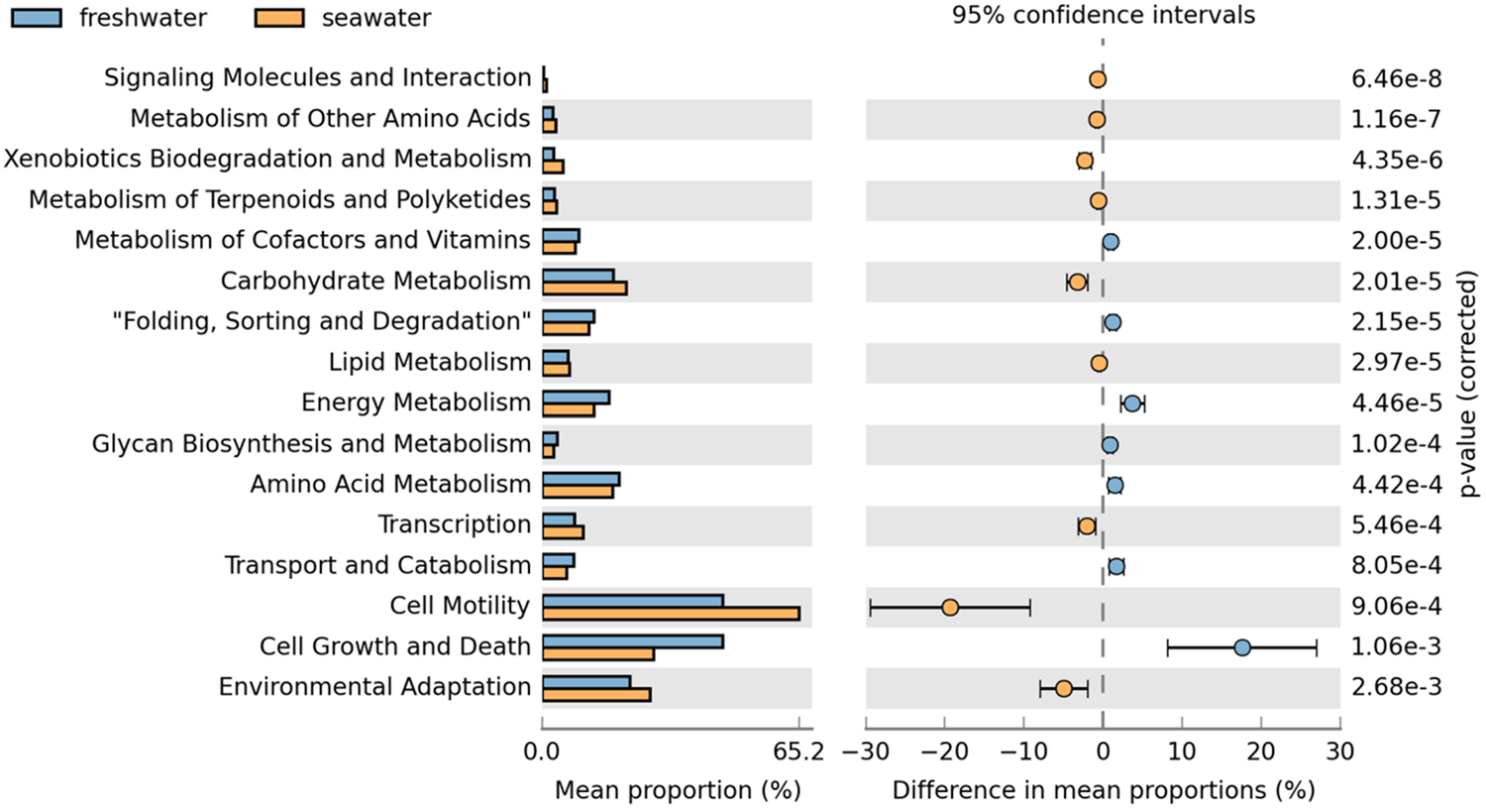
KEGG pathways found at significantly different abundances in the gut microbiota metagenomics profiles of freshwater and seawater Atlantic salmon as identified by PICRUSt and STAMP analysis. The q-values are based on Welsh’s t-test and corrected with Benjamini-Hochberg FDR.

The OTU table used for this analysis featured 2,401 OTUs, which is 463 OTUs less than with the *de novo* approach used to analyse the microbial taxonomy profiles. Accuracy was calculated in PICRUSt by NSTI (Nearest Sequenced Taxon Index), with a mean of 0.44 ± 0.01 for the FW group and 0.24 ± 0.02 for the SW group. Functional analysis revealed a significant difference in the metagenomics potential between the FW and SW group for a number of KEGG pathways (Fig. 5, Table 2). Whilst a significantly higher proportion of the combined FW bacterial metagenome was associated with the level 1 term “Genetic Information Processing”, the SW bacterial metagenome showed significantly higher abundances associated with “Environmental Information Processing” and “Metabolism”.

**Table 2:** KEGG pathways found at significantly different abundances in the gut microbiota metagenomics profiles of freshwater and seawater adapted Atlantic salmon (*Salmo salar*).

KEGG level	KEGG pathway term	FW (% of parent)	SW (% of parent)	p-values (BH corrected)^1^
1	**Cellular Processes**	3.41	3.30	0.7581
2	Transport and Catabolism	8.10	6.38	0.0015
2	Cell Motility	45.92	65.22	0.0016
3	*Bacterial motility proteins*	33*.79*	*44.94*	*0.00*3*1*
3	*Cytoskeleton proteins*	3*5.56*	*16.72*	*0.0080*
3	*Bacterial chemotaxis*	*14.82*	*18.68*	*0.0*33*8*
3	*Flagellar assembly*	*15.8*3	*19.66*	*0.0*3*51*
2	Cell Growth and Death	45.98	28.40	0.0018
3	*Apoptosis*	*5.89*	*7.58*	*0.0014*
1	**Environmental Information Processing**			
2	Signaling Molecules and Interaction	0.48	1.16	<0.0001
3	*Ion channels*	*21.58*	3*7*.3*5*	<*0.0001*
3	*Bacterial toxins*	*54.90*	*44*.33	*0.0*3*77*
1	**Genetic Information Processing**			
2	Folding, Sorting and Degradation	13.25	12.01	0.0001
2	Transcription	8.36	10.41	0.0011
1	**Metabolism**			
2	Metabolism of Other Amino Acids	2.89	3.63	<0.0001
2	Xenobiotics Biodegradation and Metabolism	3.08	5.35	<0.0001
2	Metabolism of Terpenoids and Polyketides	3.10	3.67	0.0001
2	Metabolism of Cofactors and Vitamins	9.39	8.44	0.0001
2	Carbohydrate Metabolism	18.20	21.45	0.0001
3	*C5-Branched dibasic acid metabolism*	3*.09*	*2.02*	<*0.0001*
3	*Amino sugar and nucleotide sugar metabolism*	*7.86*	*10*.3*8*	<*0.0001*
3	*Fructose and mannose metabolism*	*4.49*	*7.04*	<*0.0001*
3	*Citrate cycle (TCA cycle)*	*20.02*	*9.70*	<*0.0001*
3	*Pentose phosphate pathway*	*4*.3*5*	*6.86*	*0.0001*
3	*Ascorbate and aldarate metabolism*	*0.78*	*2.72*	*0.0012*
3	*Glyoxylate and dicarboxylate metabolism*	*7*.3*9*	*5.40*	*0.0014*
3	*Starch and sucrose metabolism*	3*.33*	*5.46*	*0.0023*
*3*	*Glycolysis*/*Gluconeogenesis*	*12.46*	*14.12*	*0.0072*
*3*	*Inositol phosphate metabolism*	*2.57*	*2.16*	*0.0131*
*3*	*Butanoate metabolism*	*9.30*	*8.16*	*0.0257*
*3*	*Galactose metabolism*	*2.51*	*3.47*	*0.0270*
2	Lipid Metabolism	6.64	7.08	0.0001
*3*	*Primary bile acid biosynthesis*	*0.17*	*0.54*	<*0.0001*
*3*	*Lipid biosynthesis proteins*	*25.78*	*20.34*	<*0.0001*
*3*	*Linoleic acid metabolism*	*0.74*	*2.61*	<*0.0001*
*3*	*Secondary bile acid biosynthesis*	*0.11*	*0.40*	<*0.0001*
*3*	*Fatty acid biosynthesis*	*21.56*	*15.73*	<*0.0001*
*3*	*Synthesis and degradation of ketone bodies*	*1.14*	*2.69*	*0.0001*
*3*	*Arachidonic acid metabolism*	*0.70*	*1.63*	*0.0001*
*3*	*Glycerolipid metabolism*	*7.51*	*13.70*	*0.0004*
*3*	*Ether lipid metabolism*	*0.12*	*0.30*	*0.0014*
*3*	*Fatty acid metabolism*	*8.85*	*12.57*	*0.0053*
2	Energy Metabolism	16.99	13.26	0.0001
*3*	*Oxidative phosphorylation*	*40.59*	*29.26*	<*0.0001*
*3*	*Nitrogen metabolism*	*5.24*	*9.14*	<*0.0001*
*3*	*Carbon fixation pathways in prokaryotes*	*18.13*	*16.61*	*0.0005*
*3*	*Methane metabolism*	*7.24*	*12.71*	*0.0008*
2	Glycan Biosynthesis and Metabolism	3.92	3.06	0.0002
2	Amino Acid Metabolism	19.57	18.06	0.0009

The % of parent refers to the respective higher level term. ^1^BH corrected = Benjamini-Hochberg correction for multiple comparisons.

Within “Environmental Information Processing”, the level 2 term “Signalling Molecules and Interaction”, showed the largest difference in significant abundance between FW and SW fish. Contained in this level 2 pathway, the level 3 term “Ion channels” was significantly more abundant in the SW fish group, than the FW fish group and “Bacterial toxins” significantly higher in the FW group compared to the SW group.

Most KEGG pathways significantly different were related to metabolism. The largest differences between FW and SW fish samples were found for the terms “Energy Metabolism” (3.7% higher in FW), “Carbohydrate metabolism” (3.2% higher in SW) and “Xenobiotics Degradation and Metabolism” (2.3% higher in SW). Within “Energy Metabolism”, the FW group showed a significantly increased capacity for “Oxidative phosphorylation” and “Carbon fixation”, whilst “Nitrogen metabolism” and “Methane metabolism” were significantly more abundant in the SW group. Within “Carbohydrate metabolism”, the most striking difference was found in the “Citrate cycle (TCA cycle)” pathway (FW = 20.0%, SW = 9.7%). Both “Amino Acid Metabolism” and Lipid Metabolism” were significantly differently abundant between FW and SW fish bacterial communities (*P* = 0.0009 and *P* = 0.0001 respectively), but the percent differences were fairly low. Amino acid metabolism indicated differing capacity for essential amino acid synthesis between FW and SW (data not shown). Within “Lipid Metabolism”, the largest differences were observed in “Fatty acid biosynthesis, “Lipid biosynthesis proteins” and “Glycerolipid metabolism”. Furthermore, the pathways “Linoleic acid metabolism” and “Arachidonic acid metabolism” were significantly more abundant in the SW group compared to the FW group.

On a deeper resolution level (KEGG level 2), pathways associated with the parent “Cellular processes” were also found significantly different in abundance, although the parent term itself was not significant. The largest differences for relative abundance to the parent term was found in this group for the level 2 pathways “Cell Motility” (19.3% increase in SW fish) and “Cell Growth and Death” (17.6% decrease in SW fish). Within “Cell Motility” the level 3 terms “Bacterial motility proteins”, “Bacterial chemotaxis” and “Flagellar Assembly” were found significantly more abundant in SW fish, whilst “Cytoskeleton proteins” was more abundant in FW fish. The only significant level 3 term within “Cell Growth and Death” was “Apoptosis”, found significantly increased in SW fish.

## Discussion

The Atlantic salmon is well-known for the successful adaptation from a freshwater (FW) living parr to an ocean going smolt, and whilst there has been much attention paid to the physiological control to allow survival, to date little attention has been given to how the intestinal microbiota changes at this critical time, with a very different environmental microbiota present. Here we examined salmon shortly before transfer to seawater (SW) and then three weeks following entry to SW, by which time we hypothesised that the marine phase of the microbiota would be established.

We found significant differences in microbial profiles in Atlantic salmon intestines correlated with transfer from FW to SW, which is in accordance with previous studies^12,17^. The microbial phyla and genera detected in the present study have also been found in previous deep-sequencing studies on salmonids^12,20^. FW fish did not only harbour an increased number of unique and higher abundant OTUs, these OTUs also mapped to a larger variety of phyla and genera compared to the OTUs unique to the SW group. Significant differences were also found in terms of the predicted and thereby the functional potential of the FW and SW microbial profile. Interestingly, microbial communities of the salmonid skin showed the opposite trend, with a higher OTU diversity found in SW fish compared to FW fish^11^. The dominance of *Mycoplasma* spp. within the SW group compared to the FW group is one of the most striking differences found in this and previous studies^12,21^. As a member of the core microbiota it may be important to the salmonid intestinal ecosystem regardless of life cycle stage. Since *Mycoplasma* species have an unusual nutritional requirement for cholesterol, related sterols and other serum factors and also have no cell wall, they are difficult to culture and therefore may have be overlooked in previous culture-based studies^21^. This reliance on cholesterol and other sterols may explain in part the difference in abundance between the FW and SW groups, as in salmonids fatty acid metabolism is known to change when fish enter the SW^22^. Interestingly, the PICRUSt and STAMP results support the hypothesis showing an increased potential for lipid metabolism in the SW group. Genes mapping to pathways associated with lipid metabolism that were found statistically more abundant in the SW group include primary and secondary bile acid biosynthesis, linoleic and arachidonic acid metabolism, synthesis and degradation of ketone bodies, glycerol- and etherlipid metabolism and fatty acid metabolism. On the other hand, genes mapping to the pathways lipid biosynthesis proteins and fatty acid biosynthesis were significantly more abundant in the FW group. Further targeted studies on this genus of microbes and lipid metabolism may be especially of interest for aquaculture and better knowledge of this relationship may be useful in the assessment of novel fatty acid sources for Atlantic salmon feed specific to life stage.

Lactic acid bacteria (LAB) are facultative anaerobes and are frequently used as probiotics, due to their reported beneficial effects on the intestinal health by the stimulation of anti-inflammatory signals^23^. OTUs of the genera *Lactobacillus, Weissella, Lactococcus* and *Streptococcus* were identified in the overall core microbiota, further indicating the importance of the LAB group to the fish. Interestingly, the distribution of LAB between the salmon in FW and SW was found significantly different for a number of genera. In SW fish, the genus *Lactobacillus* was dominant with an average of 95.23% of all LAB, followed by *Weissella* and *Streptococcus*. In FW fish, *Lactobacillus* was significantly less abundant compared to fish in SW, however, all other LAB genera were significantly more abundant. This is also reflected in the higher diversity of unique *Lactobacillus* OTUs in the SW group and a higher diversity of LAB OTUs overall in the FW group identified by Metastats analysis. The FW vs SW fish specific distributions of LAB and their implications for fish health make this group a prime target for further research.

Comparing the microbial profiles and their predicted metagenome helped define the substantial differences in community composition harboured in the FW and SW fish intestines, as well as explaining the acquisition of microbes and determinants of their composition.

Salinity gradient has been identified previously as an important shaper of microbial communities and accounts for a number of community differences of bacterioplankton in FW and SW^13,24–26^. Overcoming salinity gradients is an important colonisation barrier and the significantly reduced microbial species richness and diversity in the intestines of the SW group might reflect this evolutionary barrier^26,27^. Furthermore, the intestine of Atlantic salmon during smolting undergoes physiological changes with the transfer to the marine environment. The intestine has a relatively minor role in osmoregulation during the freshwater stage, but once exposed to seawater, it takes on roles for maintaining active fluid absorption^28^. Within the seawater environment fish are ionoregulators and actively maintain the required levels of ions at a level lower than the surrounding seawater, which is achieved by copious water uptake and targeted salt excretion^29^. The change in intestinal function is likely to result in an alteration of habitat niches for bacteria and contribute to altered community compositions. Previous studies showed that the establishment of microbes is dictated by the anatomy and physiology of the intestinal tract as well as pH, osmolality, redox potential, compartment size and structure, passage rate and residence time, which varies between freshwater and seawater fish^30^. Interestingly, predictive metagenomics analysis revealed that the pathway “Ion channels” was significantly more abundant in the SW fish group, suggesting that the bacterial community present is more adapted to the high salinity of the environment within and outside the fish. Furthermore, the potential for motility of bacteria and communication between bacteria was significantly increased in SW fish, which may reflect a preferred enrichment of bacteria that are able to adapt to a changing environment by finding suitable niches through motility and interacting with the wider microbial community.

In commercial aquaculture, diets are adapted following seawater transfer. Such changes could impact on microbial composition, but in the context of this research this was not examined. However, differences in the metagenomics potential between the FW and SW microbial communities in terms of metabolism were identified. Of note is the increased capacity of the microbial FW community for “Oxidative phosphorylation” and “Citrate cycle”, suggesting that more free energy is available to the teleost host, which can be used for the necessary changes during smoltification and energy requirements during the subsequent osmoregulatory adaptation. Another interesting aspect is the difference of metagenomics potential of the FW and SW microbial communities to produce amino acids essential to the teleost host. Important aspects of lipid metabolism were also found differently abundant in the predicted metagenome of the FW and SW groups, which has been discussed above.

Despite the observed differences between the FW and SW group, the identification of a strong core microbiota suggests a level of plasticity by retaining microbes needed for minimum function of the intestinal environment. Previous studies provided evidence that intestinal communities differ significantly from the water communities^17^ and dietary items^31^ and did not share abundant OTUs with either source. In a recent study it was found that the proportion of water and diet derived bacteria declined over time, as they are potentially unable to thrive after establishment of microbes adapted to life in the intestine^20^. The distinct clustering of microbes within and outside the host have also been identified in previous studies^11,15,16^, which suggests that there is a more deterministic host selection process for microbes than just passive uptake from the environment or diet. This “host-effect” system refers to the sub-set of environmental microbes able to overcome host defences and colonise niches within the intestine^17^. Some high abundance organisms within the intestine can be found at very low abundances in the water column and diet, therefore selective retention of appropriate microbes takes place through a “habitat filter” applied by host physiology and immunity^17,31^.

Additionally, some microbes present in fish subject to high salinities have also been identified at low abundances in fish kept at low salinities^17^, which is in agreement with the present study. These opportunistic members of the rare biosphere may thrive better after the changes in physiology and biochemistry in the intestine of SW adapted fish and outcompete previously better adapted “FW” bacteria^17^. Such effects have been proposed for oceanic and FW stickleback populations, separated by habitat for at least 10,000 years, which share a number of microbes in a culture-dependent study^32^. The results of these previous studies combined with our findings points towards a scenario where competitive niche appropriation is determining microbial assemblages as opposed to a solely stochastic colonisation from the surrounding water.

Homeostasis of microbe communities is essential for the immune system and therefore fish health^20^. The identification of key microbial species that could be used as early indicators for dysbiosis may be of great interest for aquaculture. To date, there is still a lack of knowledge of what constitutes a “healthy” microbiota in fish. Therefore further census studies of microbial communities at different life stages of fish, from different genetic backgrounds, at different locations and under different rearing conditions are vital to assess how these factors can influence the microbial compositions and identify key microbes that are stably maintained. Additionally, comparing the microbial communities of fish that are diagnosed “healthy” with fish infected with a pathogen, may enable the detection of microbial biomarkers that could in future be monitored for early disease detection.

## Material and Methods

### Fish maintenance and sampling

Freshwater pre-smolts (FW) were sampled at a commercial flow through hatchery two weeks prior to saltwater transfer. The fish were allowed to acclimate to the saltwater for three weeks before they were sampled (SW). Both groups of fish sampled were from the same cohort and represented an out-bred mixed sex farmed salmon strain. All fish were maintained under natural light conditions and natural fluctuating temperature and pH levels. The fish were fed with a fishmeal rich commercial diet appropriate for their life cycle stage (BioMar).

At each sampling point 20 fish were killed by anaesthesia overdose (phenoxyethanol) followed by destruction of the brain (schedule 1 method of killing). The study was approved by the University of Aberdeen ethics committee and the killing method was in accordance to the UK Animals (Scientific Procedures) Act 1986. The final weights of the fish were approximately 105 g and 120 g for the freshwater and seawater groups respectively.

The digesta samples were collected in 1.5 ml Eppendorf tubes immediately following death and frozen on dry ice before longer term storage at −80 °C.

### DNA extraction

DNA extraction was performed on 100–200 mg of digesta as previously described^18^. In short, digesta in InhibitEx buffer (Qiagen) was mechanically broken down by TissueLyser, after which the QIAamp Fast DNA Stool Mini Kit (Qiagen) was followed according to the manufacturer’s protocol. DNA was quantified by NanoDrop spectrometry.

### PCR amplification and sequencing

Primer targets spanning the V3 and V4 region of the 16S rRNA gene had the following sequences: F 5′ TCGTCGGCAGCGTCAGATGTGTATAAGAGACAG**CCTACGGGNGGCWGCAG** and R 5′GTCTCGTGGGCTCGGAGATGTGTATAAGAGACAG**GACTACHVGGGTATCTAATCC** as described before^19^.

PCR reactions were performed in triplicate for each sample as described previously^18^. PCR products verified by gel electrophoresis were cleaned with AMPure XP (Agencourt) and verification of clean-up product with TapeStation (Agilent) was performed, before they were indexed with Nextera XT barcodes. After another round of AMPure XP (Agencourt) clean-up, multiplexed amplicons were sequenced with Illumina MiSeq from both ends.

### Bioinformatic analysis

Initial forward and reverse read assembly and quality control was performed using mothur^33^. All reads that were <400 bp long, had a Phred quality score average <25, a homopolymer run >10 bases and nucleotide differences >7 bases were excluded. The remaining contigs were imported into QIIME, which was used for all further taxonomic analysis^34^. Uclust was used to pick Operational taxonomic units (OTUs) *de novo* at the 97% similarity level. Taxonomy of the representative OTU set was assigned using the RDP classifier^35^ at an 80% confidence level. Taxonomy-assigned sequences were aligned with PyNAST with a pairwise Uclust alignment algorithm against the Greengenes data base^36^ (Release GG_13_5). Chimeric sequences were identified by ChimeraSlayer and removed from both the OTU table and the alignment. All OTUs mapping to, Cyanobacteria, “Other Bacteria” (OB) and “Unclassified Bacteria” (UB) were removed, as well as singleton OTUs. The final quality-filtered alignment was used to compute core microbiota and identify microbial profiles. Shared and significantly different OTUs between the freshwater and seawater fish were identified by Metastats^37^. The program STAMP (Statistical Analysis of Metagenomics Profiles) was used to test for statistically significant differences between the members of the LAB order *Lactobacillales* using Welch’s t-test corrected for multiple-testing by Benjamini-Hochberg false discovery rate (FDR)^38,39^.

Alpha diversity statistics were calculated for richness and diversity metrics. Rarefaction curves were computed from which the highest common rarefaction level of all samples was identified and used as the basis to test for statistical differences between FW and SW samples through beta-diversity analysis (phylogenetic differences between life stage groups). The distance matrixes for weighted and unweighted Unifrac metrics were used to produce Multiple Dimension Scale (MDS) plots^40,41^. Visualisation of rarefaction plots and cluster analysis was done with the Phyloseq package in R^42^.

For functional analysis of the microbial profiles of FW and SW samples, OTU tables were produced using closed-reference picking, which is pre-requisite for the prediction of metagenomes using PICRUSt (Phylogenetic Investigation of Communities by Reconstruction of Unobserved States)^43^. After normalisation for 16S rRNA copy numbers, metagenomes were predicted based on KEGG terms and summarised by KEGG term function. KEGG levels 1, 2 and 3 were considered and statistical differences calculated in relation to their parent term. The program STAMP was used to test for statistically significant differences between the microbial profiles using Welch’s t-test corrected for multiple-testing by Benjamini-Hochberg false discovery rate (FDR)^38,39^.

## Electronic supplementary material

Supplementary File S1

Supplementary File 2
