# Supplementary material for: Seawater transfer alters the intestinal microbiota profiles of Atlantic salmon (Salmo salar L.)
Source: Sci Rep. 2017 Oct 24;7:13877. doi: 10.1038/s41598-017-13249-8 (PMC5654775; doi:10.1038/s41598-017-13249-8)
Supplement: Supplementary file 2 [file 41598_2017_13249_MOESM2_ESM.doc]

**Seawater transfer alters the intestinal microbiota profiles of Atlantic salmon (*Salmo salar* L.)**

Carola E. Dehler^a^, Christopher J. Secombes^a^, Samuel A.M. Martin^a^,*

^a^ Institute of Biological and Environmental Sciences, University of Aberdeen, Tillydrone Avenue, Aberdeen, AB24 2TZ, UK

* Corresponding author: sam.martin@abdn.ac.uk, Phone: +44 (0)1224 272867

Figure S2. Relative abundance (%) of main bacterial taxa found in digesta collected from the distal intestine of Atlantic salmon at phylum level. Phyla below an abundance of 0.1% are not shown but summarised in a mixed group “< 0.1% abundance”. Bacterial profiles are shown on an individual level, by fresh- or seawater group (FW=freshwater pre-smolt and SW=seawater post-smolt) and overall level (=all).

**
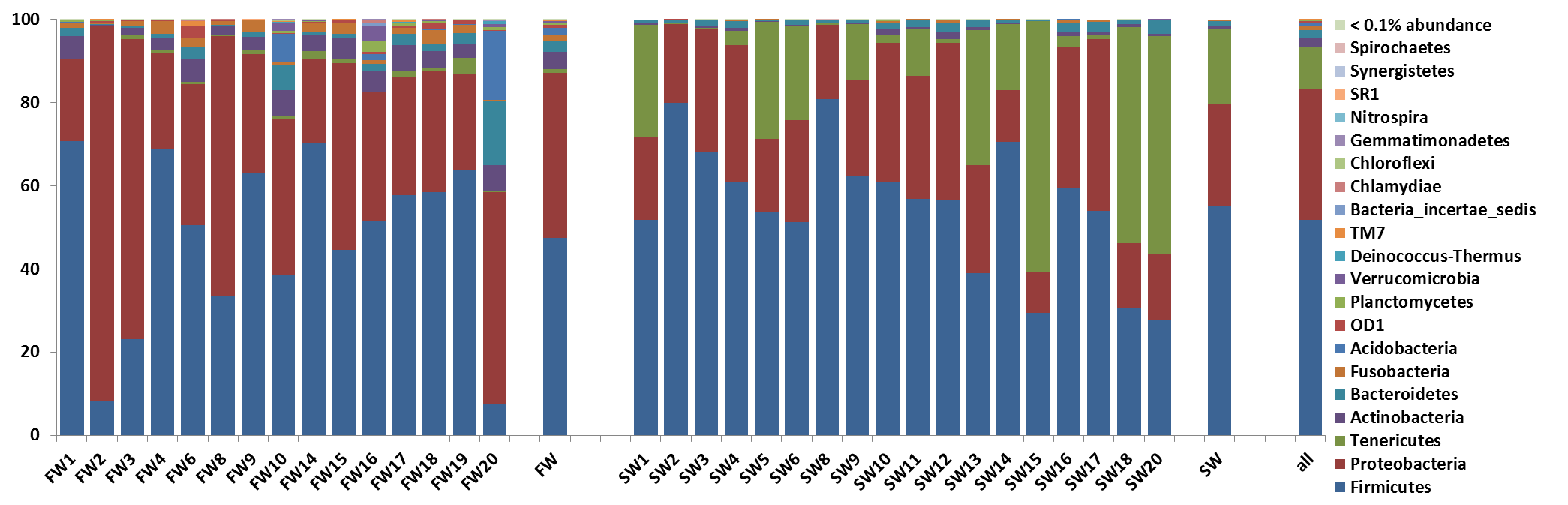
**

Figure S3. Relative abundance (%) of the 50 main bacterial taxa found in digesta collected from the distal intestine of Atlantic salmon at genus level. Phyla at low abundances are not shown but summarised in a mixed group “Others”. Bacterial profiles are shown for the fresh- and seawater group (FW=freshwater pre-smolt and SW=seawater post-smolt) and overall.

**
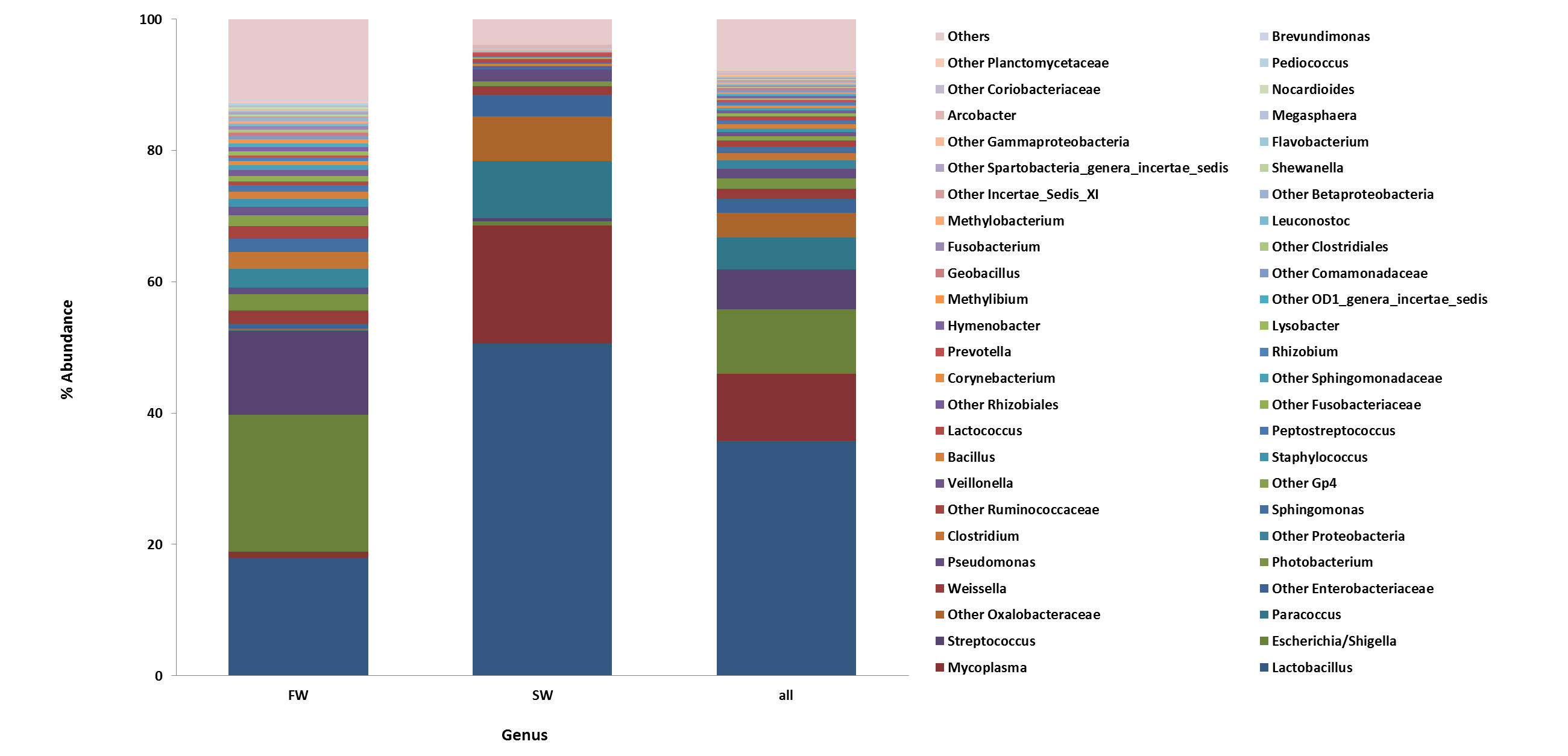
**

Figure S4. Distribution of lactic acid bacteria (LAB) genera within the order *Lactobacillales* found significantly differently abundant in the distal digesta of Atlantic salmon kept in freshwater and seawater. Mean proportion (%) is based on all genera identified within the order *Lactobacillales*, whereas bar graphs are only shown for significantly different genera. The q-values are based on Benjamini-Hochberg corrected p-values that resulted from Welch’s t-tests.


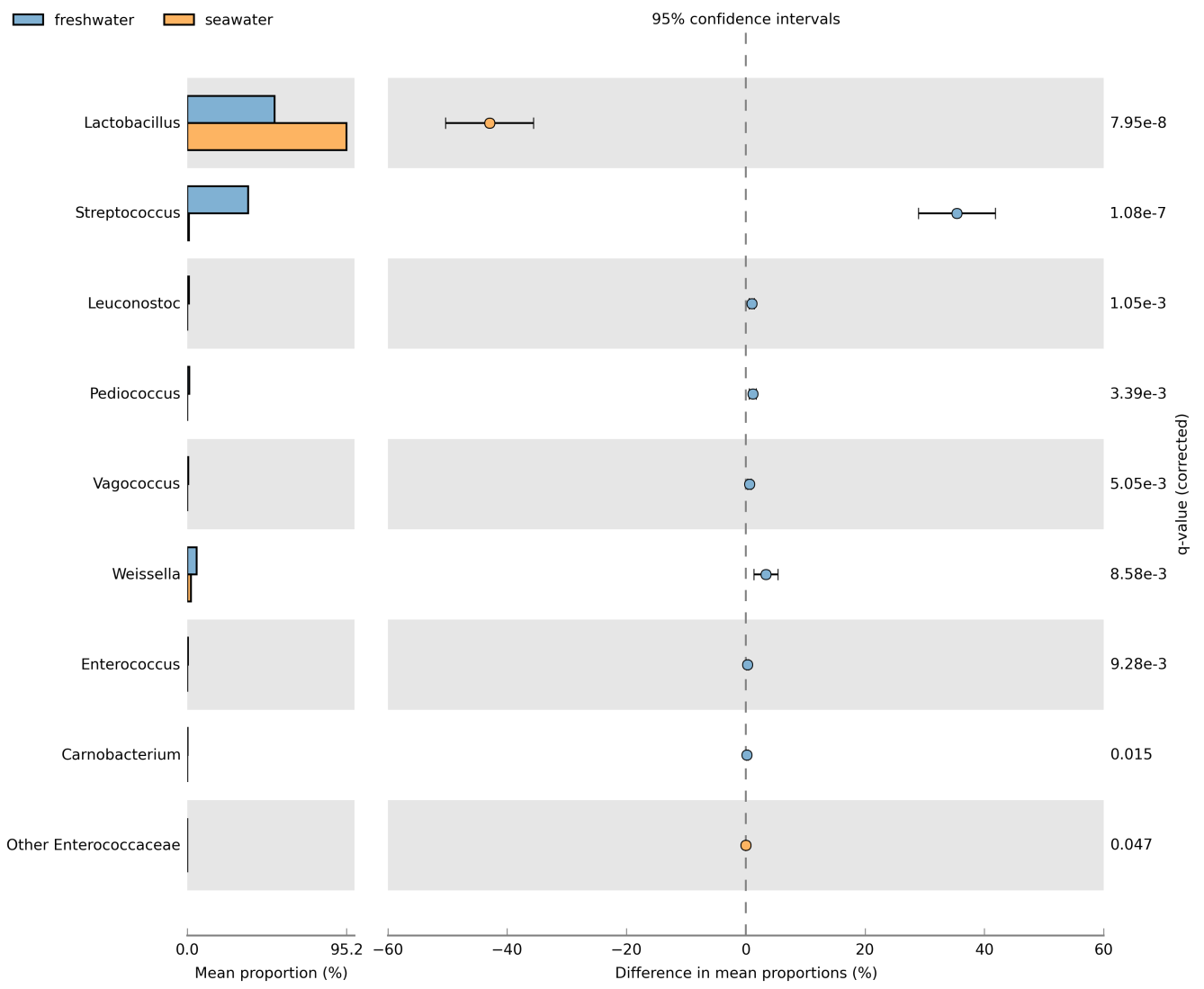


**Table S5: Full list of Operational Taxonomic Units (OTUs) found uniquely in the distal intestine of freshwater Atlantic salmon, as identified by Metastats analysis.**

| **denovo ID^1^** | **Greengenes ID^2^** | **Phylum** | **Taxonomic assignment** | **Blast %^3^** | **mean % FW** | **pvalue** |
| --- | --- | --- | --- | --- | --- | --- |
| 103108 | 151717 | Acidobacteria | Other Gp1 | 97.8 | 0.03592 | 0.0010 |
| 43984 | 221248 | Acidobacteria | Other Gp1 | 98.5 | 0.00505 | 0.0010 |
| 50860 | 1145745 | Acidobacteria | Other Gp1 | 97.8 | 0.00228 | 0.0001 |
| 50008 | 4447214 | Acidobacteria | Other Gp1 | 98.5 | 0.00143 | 0.0307 |
| 23658 | 790392 | Acidobacteria | Other Gp3 | 97.8 | 0.03221 | 0.0010 |
| 59159 | 1119336 | Acidobacteria | Other Gp3 | 98.5 | 0.00263 | 0.0010 |
| 65073 | 160412 | Acidobacteria | Other Gp3 | 92.5 | 0.00241 | 0.0030 |
| 108041 | 4374731 | Acidobacteria | Other Gp3 | 97.0 | 0.00179 | 0.0010 |
| 60964 | 213061 | Acidobacteria | Other Gp4 | 97.8 | 0.09557 | 0.0010 |
| 96221 | 509916 | Acidobacteria | Other Gp4 | 97.5 | 0.07123 | 0.0010 |
| 16617 | 510646 | Acidobacteria | Other Gp4 | 97.3 | 0.01808 | 0.0010 |
| 30882 | 4319244 | Acidobacteria | Other Gp4 | 92.3 | 0.01625 | 0.0010 |
| 63009 | 207176 | Acidobacteria | Other Gp4 | 96.0 | 0.00732 | 0.0010 |
| 6820 | 560335 | Acidobacteria | Other Gp4 | 92.7 | 0.00723 | 0.0010 |
| 18221 | 1032520 | Acidobacteria | Other Gp4 | 92.9 | 0.00708 | <0.0001 |
| 107625 | 155455 | Acidobacteria | Other Gp4 | 95.8 | 0.00413 | 0.0003 |
| 100004 | 4432930 | Acidobacteria | Other Gp4 | 95.8 | 0.00263 | 0.0010 |
| 106886 | 207176 | Acidobacteria | Other Gp4 | 95.5 | 0.00108 | 0.0307 |
| 61052 | 868045 | Acidobacteria | Other Gp4 | 93.5 | 0.00048 | 0.0096 |
| 5041 | 218462 | Acidobacteria | Other Gp6 | 92.1 | 0.00912 | <0.0001 |
| 29507 | 221791 | Acidobacteria | Other Gp7 | 92.5 | 0.00114 | 0.0096 |
| 27800 | 112867 | Acidobacteria | Other Gp16 | 97.3 | 0.02366 | 0.0010 |
| 89872 | 833957 | Acidobacteria | Other Gp16 | 95.5 | 0.00513 | 0.0010 |
| 17623 | 833957 | Acidobacteria | Other Gp16 | 96.3 | 0.00449 | 0.0030 |
| 133748 | 82007 | Acidobacteria | Other Gp16 | 97.0 | 0.00143 | 0.0030 |
| 75031 | 206963 | Acidobacteria | Other Gp16 | 97.3 | 0.00086 | 0.0307 |
|  |  |  |  |  |  |  |
| 34065 | 571103 | Actinobacteria | Actinomyces | 92.2 | 0.00143 | <0.0001 |
| 55696 | 698236 | Actinobacteria | Actinomycetospora | 93.8 | 0.00625 | 0.0010 |
| 6989 | 134209 | Actinobacteria | Arthrobacter | 96.3 | 0.00583 | 0.0010 |
| 5798 | 256173 | Actinobacteria | Arthrobacter | 92.3 | 0.00048 | 0.0096 |
| 10718 | 139478 | Actinobacteria | Atopobium | 94.6 | 0.00257 | 0.0009 |
| 121355 | 897946 | Actinobacteria | Bifidobacterium | 97.1 | 0.00400 | 0.0003 |
| 862 | 250241 | Actinobacteria | Blastococcus | 95.3 | 0.00119 | <0.0001 |
| 42007 | 729697 | Actinobacteria | Conexibacter | 92.0 | 0.01368 | 0.0010 |
| 133673 | 974817 | Actinobacteria | Conexibacter | 92.7 | 0.01067 | 0.0010 |
| 120447 | 799959 | Actinobacteria | Conexibacter | 92.7 | 0.00743 | 0.0010 |
| 41068 | 799959 | Actinobacteria | Conexibacter | 91.8 | 0.00598 | 0.0010 |
| 61094 | 799959 | Actinobacteria | Conexibacter | 92.0 | 0.00526 | 0.0010 |
| 111796 | 1060353 | Actinobacteria | Conexibacter | 91.3 | 0.00145 | 0.0307 |
| 48064 | 799959 | Actinobacteria | Conexibacter | 91.3 | 0.00084 | 0.0003 |
| 121801 | 799959 | Actinobacteria | Conexibacter | 91.1 | 0.00036 | 0.0307 |
| 61693 | 465989 | Actinobacteria | Corynebacterium | 97.0 | 0.00461 | <0.0001 |
| 101084 | 460393 | Actinobacteria | Corynebacterium | 98.0 | 0.00072 | 0.0009 |
| 27732 | 582047 | Actinobacteria | Demequina | 96.6 | 0.00143 | 0.0030 |
| 94124 | 108722 | Actinobacteria | Granulicoccus | 96.6 | 0.00317 | 0.0030 |
| 82678 | 840275 | Actinobacteria | Iamia | 98.0 | 0.00048 | 0.0096 |
| 12720 | 1015820 | Actinobacteria | Illumatobacter | 98.3 | 0.05191 | 0.0010 |
| 79493 | 606789 | Actinobacteria | Illumatobacter | 98.0 | 0.00858 | 0.0010 |
| 83130 | 1011476 | Actinobacteria | Illumatobacter | 97.8 | 0.00674 | 0.0010 |
| 7956 | 813287 | Actinobacteria | Illumatobacter | 97.8 | 0.00241 | 0.0009 |
| 1961 | 1024739 | Actinobacteria | Illumatobacter | 98.0 | 0.00241 | 0.0001 |
| 111167 | 1021335 | Actinobacteria | Kineosporia | 95.6 | 0.00150 | 0.0096 |
| 8095 | 283765 | Actinobacteria | Kocuria | 95.3 | 0.00428 | 0.0010 |
| 101460 | 149895 | Actinobacteria | Kocuria | 95.8 | 0.00340 | <0.0001 |
| 87895 | 1146125 | Actinobacteria | Leifsonia | 96.1 | 0.01355 | 0.0010 |
| 60219 | 902698 | Actinobacteria | Marmoricola | 96.3 | 0.02376 | 0.0010 |
| 28678 | 996116 | Actinobacteria | Marmoricola | 95.6 | 0.00220 | 0.0030 |
| 114355 | 996116 | Actinobacteria | Marmoricola | 94.9 | 0.00161 | 0.0096 |
| 107473 | 13236 | Actinobacteria | Mycobacterium | 93.6 | 0.01984 | 0.0010 |
| 79102 | 2651333 | Actinobacteria | Mycobacterium | 93.8 | 0.00715 | <0.0001 |
| 92682 | 1088254 | Actinobacteria | Nocardioides | 94.8 | 0.06174 | 0.0010 |
| 122113 | 875049 | Actinobacteria | Nocardioides | 94.3 | 0.02475 | 0.0010 |
| 42392 | 875049 | Actinobacteria | Nocardioides | 92.9 | 0.00758 | 0.0010 |
| 69072 | 1105450 | Actinobacteria | Nocardioides | 95.4 | 0.00215 | 0.0010 |
| 41084 | 365033 | Actinobacteria | Olsenella | 97.5 | 0.06767 | 0.0010 |
| 112988 | 4361768 | Actinobacteria | Olsenella | 96.6 | 0.01297 | 0.0010 |
| 123700 | 521275 | Actinobacteria | Olsenella | 94.6 | 0.01193 | 0.0010 |
| 100258 | 99332 | Actinobacteria | Olsenella | 96.6 | 0.00399 | <0.0001 |
| 123139 | 153998 | Actinobacteria | Other Acidimicrobiales | 96.8 | 0.01183 | 0.0010 |
| 76520 | 410072 | Actinobacteria | Other Acidimicrobiales | 97.3 | 0.00188 | 0.0030 |
| 39913 | 225425 | Actinobacteria | Other Acidimicrobiales | 96.0 | 0.00048 | 0.0096 |
| 20903 | 235293 | Actinobacteria | Other Actinobacteria | 91.6 | 0.03325 | 0.0010 |
| 112959 | 4441019 | Actinobacteria | Other Actinobacteria | 95.3 | 0.01867 | 0.0010 |
| 72449 | 4310013 | Actinobacteria | Other Actinobacteria | 93.0 | 0.01537 | 0.0010 |
| 91544 | 529793 | Actinobacteria | Other Actinobacteria | 98.0 | 0.01237 | 0.0010 |
| 36243 | 4314391 | Actinobacteria | Other Actinobacteria | 90.6 | 0.00999 | 0.0010 |
| 22050 | 617900 | Actinobacteria | Other Actinobacteria | 98.3 | 0.00241 | 0.0030 |
| 128771 | 570369 | Actinobacteria | Other Actinobacteria | 95.3 | 0.00241 | 0.0030 |
| 128971 | 2442916 | Actinobacteria | Other Actinobacteria | 97.3 | 0.00201 | 0.0030 |
| 965 | 4461906 | Actinobacteria | Other Actinobacteria | 96.4 | 0.00114 | 0.0096 |
| 117650 | 893916 | Actinobacteria | Other Actinomycetales | 96.6 | 0.02879 | 0.0010 |
| 56220 | 102993 | Actinobacteria | Other Actinomycetales | 97.3 | 0.02258 | 0.0010 |
| 102509 | 801612 | Actinobacteria | Other Actinomycetales | 97.5 | 0.00885 | 0.0010 |
| 28051 | 142681 | Actinobacteria | Other Actinomycetales | 97.3 | 0.00842 | <0.0001 |
| 94218 | 952659 | Actinobacteria | Other Actinomycetales | 96.3 | 0.00571 | <0.0001 |
| 9453 | 1018122 | Actinobacteria | Other Actinomycetales | 97.1 | 0.00513 | 0.0010 |
| 100559 | 97567 | Actinobacteria | Other Actinomycetales | 97.3 | 0.00477 | 0.0001 |
| 99594 | 719335 | Actinobacteria | Other Actinomycetales | 96.6 | 0.00456 | 0.0010 |
| 11885 | 241899 | Actinobacteria | Other Actinomycetales | 96.6 | 0.00323 | 0.0010 |
| 9095 | 259094 | Actinobacteria | Other Actinomycetales | 96.6 | 0.00205 | 0.0030 |
| 109371 | 3905299 | Actinobacteria | Other Actinomycetales | 97.5 | 0.00145 | 0.0307 |
| 93673 | 890516 | Actinobacteria | Other Actinomycetales | 95.3 | 0.00132 | 0.0030 |
| 48203 | 4481011 | Actinobacteria | Other Actinomycetales | 96.5 | 0.00108 | <0.0001 |
| 44336 | 760806 | Actinobacteria | Other Actinomycetales | 95.1 | 0.00084 | 0.0003 |
| 116345 | 240553 | Actinobacteria | Other Actinomycetales | 96.3 | 0.00072 | 0.0009 |
| 118001 | 406112 | Actinobacteria | Other Actinomycetales | 96.1 | 0.00072 | 0.0009 |
| 92105 | 1903534 | Actinobacteria | Other Coriobacteraceae | 97.1 | 0.00359 | 0.0096 |
| 82621 | 435928 | Actinobacteria | Other Microbacteriaceae | 95.6 | 0.00238 | 0.0030 |
| 115607 | 826611 | Actinobacteria | Other Microbacteriaceae | 95.6 | 0.00214 | 0.0030 |
| 28711 | 948180 | Actinobacteria | Other Microbacteriaceae | 95.8 | 0.00096 | 0.0001 |
| 56971 | 335422 | Actinobacteria | Other Micrococcaceae | 96.1 | 0.00728 | 0.0010 |
| 135218 | 931593 | Actinobacteria | Other Micromonosporaceae | 97.1 | 0.00627 | 0.0010 |
| 103297 | 4434807 | Actinobacteria | Other Micromonosporaceae | 96.1 | 0.00472 | 0.0001 |
| 15992 | 220524 | Actinobacteria | Other Nakamurellaceae | 96.8 | 0.05588 | 0.0010 |
| 93395 | 905618 | Actinobacteria | Other Nocardioidaceae | 92.6 | 0.00296 | 0.0009 |
| 101242 | 174732 | Actinobacteria | Other Propionibacteriaceae | 96.8 | 0.00761 | 0.0010 |
| 56912 | 1107468 | Actinobacteria | Other Propionibacteriaceae | 95.6 | 0.00145 | 0.0307 |
| 39422 | 4419473 | Actinobacteria | Other Solirubrobacterales | 91.3 | 0.00434 | <0.0001 |
| 21860 | 244375 | Actinobacteria | Other Solirubrobacterales | 90.9 | 0.00335 | 0.0010 |
| 21779 | 960234 | Actinobacteria | Other Solirubrobacterales | 91.8 | 0.00299 | 0.0010 |
| 92008 | 799959 | Actinobacteria | Other Solirubrobacterales | 90.4 | 0.00131 | <0.0001 |
| 127396 | 4411088 | Actinobacteria | Other Thermomonosporaceae | 96.6 | 0.02936 | 0.0010 |
| 78649 | 2438948 | Actinobacteria | Parascardovia | 96.8 | 0.00622 | 0.0030 |
| 44594 | 114587 | Actinobacteria | Pseudonocardia | 96.8 | 0.01316 | 0.0010 |
| 131828 | 1136762 | Actinobacteria | Pseudonocardia | 97.3 | 0.01140 | 0.0010 |
| 124156 | 665877 | Actinobacteria | Sporichthya | 97.1 | 0.00846 | 0.0010 |
| 78729 | 1009173 | Actinobacteria | Williamsia | 95.1 | 0.00281 | 0.0003 |
| 31195 | 971353 | Actinobacteria | Yaniella | 96.1 | 0.00631 | <0.0001 |
|  |  |  |  |  |  |  |
| 54293 | 1065314 | Bacteroidetes | Adhaeribacter | 93.6 | 0.00241 | 0.0030 |
| 35561 | 4356331 | Bacteroidetes | Bacteroides | 93.4 | 0.01966 | 0.0010 |
| 3717 | 6964777 | Bacteroidetes | Bacteroides | 90.5 | 0.00512 | <0.0001 |
| 51881 | 4312724 | Bacteroidetes | Chitinophaga | 93.6 | 0.08381 | 0.0010 |
| 28339 | 1002658 | Bacteroidetes | Chitinophaga | 93.6 | 0.01034 | 0.0010 |
| 106815 | 3329353 | Bacteroidetes | Chryseobacterium | 94.1 | 0.00809 | <0.0001 |
| 95403 | 4478426 | Bacteroidetes | Chryseobacterium | 94.1 | 0.00570 | <0.0001 |
| 44533 | 1087462 | Bacteroidetes | Cytophaga | 93.3 | 0.01446 | 0.0010 |
| 61110 | 736771 | Bacteroidetes | Ferruginibacter | 93.8 | 0.01422 | 0.0010 |
| 38276 | 983307 | Bacteroidetes | Ferruginibacter | 91.5 | 0.01205 | 0.0010 |
| 79382 | 1013954 | Bacteroidetes | Ferruginibacter | 92.7 | 0.01075 | 0.0010 |
| 131177 | 4446565 | Bacteroidetes | Ferruginibacter | 92.2 | 0.00675 | <0.0001 |
| 86210 | 221734 | Bacteroidetes | Ferruginibacter | 93.6 | 0.00179 | 0.0010 |
| 15306 | 983307 | Bacteroidetes | Ferruginibacter | 93.8 | 0.00155 | <0.0001 |
| 54302 | 873090 | Bacteroidetes | Ferruginibacter | 89.7 | 0.00084 | 0.0096 |
| 88256 | 570107 | Bacteroidetes | Ferruginibacter | 93.1 | 0.00048 | 0.0096 |
| 111951 | 983307 | Bacteroidetes | Ferruginibacter | 92.9 | 0.00036 | 0.0307 |
| 87200 | 896098 | Bacteroidetes | Flavobacterium | 94.1 | 0.02246 | 0.0010 |
| 102087 | 719030 | Bacteroidetes | Hymenobacter | 93.6 | 0.09469 | 0.0010 |
| 99043 | 513548 | Bacteroidetes | Hymenobacter | 92.7 | 0.06170 | 0.0010 |
| 48568 | 789806 | Bacteroidetes | Hymenobacter | 92.9 | 0.03190 | 0.0010 |
| 97849 | 976450 | Bacteroidetes | Hymenobacter | 93.8 | 0.02048 | 0.0010 |
| 80955 | 513548 | Bacteroidetes | Hymenobacter | 91.7 | 0.01118 | 0.0010 |
| 41793 | 936454 | Bacteroidetes | Hymenobacter | 89.6 | 0.00920 | 0.0010 |
| 16740 | 822229 | Bacteroidetes | Hymenobacter | 92.4 | 0.00886 | 0.0010 |
| 59347 | 4404139 | Bacteroidetes | Hymenobacter | 92.7 | 0.00442 | 0.0010 |
| 103433 | 827969 | Bacteroidetes | Hymenobacter | 91.5 | 0.00335 | 0.0010 |
| 128801 | 4468694 | Bacteroidetes | Hymenobacter | 92.2 | 0.00251 | 0.0010 |
| 90074 | 555981 | Bacteroidetes | Hymenobacter | 91.7 | 0.00191 | 0.0010 |
| 81869 | 4404139 | Bacteroidetes | Hymenobacter | 91.9 | 0.00180 | 0.0009 |
| 25169 | 4468694 | Bacteroidetes | Hymenobacter | 93.6 | 0.00167 | <0.0001 |
| 101531 | 1027943 | Bacteroidetes | Hymenobacter | 89.6 | 0.00145 | 0.0307 |
| 120440 | 4468694 | Bacteroidetes | Hymenobacter | 90.8 | 0.00145 | 0.0307 |
| 18057 | 4404139 | Bacteroidetes | Hymenobacter | 92.4 | 0.00119 | <0.0001 |
| 64606 | 513548 | Bacteroidetes | Hymenobacter | 90.5 | 0.00072 | 0.0009 |
| 75778 | 1090978 | Bacteroidetes | Hymenobacter | 92.2 | 0.00048 | 0.0096 |
| 107006 | 966903 | Bacteroidetes | Hymenobacter | 92.2 | 0.00048 | 0.0096 |
| 47394 | 4404139 | Bacteroidetes | Hymenobacter | 91.2 | 0.00036 | 0.0307 |
| 8503 | 583939 | Bacteroidetes | Mucilaginibacter | 93.6 | 0.00603 | 0.0010 |
| 74931 | 200923 | Bacteroidetes | Myroides | 91.7 | 0.01046 | <0.0001 |
| 115580 | 4389652 | Bacteroidetes | Nubsella | 93.8 | 0.00161 | 0.0096 |
| 66580 | 1110064 | Bacteroidetes | Other Bacteroidetes | 83.4 | 0.03679 | 0.0010 |
| 41923 | 4469493 | Bacteroidetes | Other Bacteroidetes | 93.8 | 0.02524 | 0.0010 |
| 88877 | 307869 | Bacteroidetes | Other Bacteroidetes | 93.6 | 0.02323 | 0.0010 |
| 87409 | 2446610 | Bacteroidetes | Other Bacteroidetes | 88.6 | 0.00385 | <0.0001 |
| 122030 | 1040768 | Bacteroidetes | Other Bacteroidetes | 92.7 | 0.00265 | 0.0010 |
| 133137 | 4444621 | Bacteroidetes | Other Bacteroidetes | 93.1 | 0.00263 | 0.0003 |
| 31309 | 777827 | Bacteroidetes | Other Bacteroidetes | 93.8 | 0.00188 | 0.0030 |
| 127471 | 55917 | Bacteroidetes | Other Bacteroidetes | 93.6 | 0.00086 | 0.0307 |
| 112106 | 885618 | Bacteroidetes | Other Chitinophagaceae | 93.8 | 0.07668 | 0.0010 |
| 110504 | 1091635 | Bacteroidetes | Other Chitinophagaceae | 94.1 | 0.05627 | 0.0010 |
| 56839 | 4044057 | Bacteroidetes | Other Chitinophagaceae | 93.1 | 0.03022 | 0.0010 |
| 21493 | 442533 | Bacteroidetes | Other Chitinophagaceae | 90.8 | 0.02285 | 0.0010 |
| 15947 | 137583 | Bacteroidetes | Other Chitinophagaceae | 91.2 | 0.00827 | 0.0010 |
| 52340 | 219534 | Bacteroidetes | Other Chitinophagaceae | 93.1 | 0.00485 | 0.0010 |
| 104595 | 934090 | Bacteroidetes | Other Chitinophagaceae | 91.5 | 0.00370 | 0.0010 |
| 132051 | 557323 | Bacteroidetes | Other Chitinophagaceae | 90.8 | 0.00337 | 0.0003 |
| 126316 | 160010 | Bacteroidetes | Other Chitinophagaceae | 92.4 | 0.00241 | 0.0030 |
| 14200 | 4203053 | Bacteroidetes | Other Chitinophagaceae | 88.9 | 0.00191 | 0.0010 |
| 71639 | 257602 | Bacteroidetes | Other Chitinophagaceae | 91.9 | 0.00191 | 0.0010 |
| 126150 | 4296430 | Bacteroidetes | Other Chitinophagaceae | 93.8 | 0.00145 | 0.0307 |
| 56946 | 885618 | Bacteroidetes | Other Chitinophagaceae | 91.5 | 0.00131 | <0.0001 |
| 12574 | 4301516 | Bacteroidetes | Other Chitinophagaceae | 93.4 | 0.00120 | 0.0096 |
| 68101 | 885618 | Bacteroidetes | Other Chitinophagaceae | 89.4 | 0.00048 | 0.0096 |
| 54364 | 912669 | Bacteroidetes | Other Chitinophagaceae | 91.9 | 0.00036 | 0.0307 |
| 135235 | 4305621 | Bacteroidetes | Other Cytophagaceae | 91.0 | 0.00161 | 0.0096 |
| 132228 | 353686 | Bacteroidetes | Other Flavobacteriaceae | 94.1 | 0.00299 | 0.0003 |
| 67440 | 4446973 | Bacteroidetes | Other Porphyromonadaceae | 93.1 | 0.01825 | 0.0010 |
| 32491 | 1115121 | Bacteroidetes | Other Porphyromonadaceae | 93.8 | 0.00555 | <0.0001 |
| 128411 | 155632 | Bacteroidetes | Other Sphingobacteriaceae | 91.0 | 0.01925 | 0.0010 |
| 97578 | 258308 | Bacteroidetes | Other Sphingobacteriaceae | 87.9 | 0.01333 | 0.0010 |
| 68858 | 974905 | Bacteroidetes | Other Sphingobacteriales | 94.3 | 0.00502 | 0.0010 |
| 105444 | 4359222 | Bacteroidetes | Prevotella | 93.8 | 0.00969 | 0.0010 |
| 106515 | 4454385 | Bacteroidetes | Prevotella | 93.4 | 0.00571 | <0.0001 |
| 443 | 557665 | Bacteroidetes | Prevotella | 93.6 | 0.00114 | 0.0096 |
| 47085 | 307571 | Bacteroidetes | Prevotella | 93.1 | 0.00072 | 0.0009 |
| 111383 | 702181 | Bacteroidetes | Segetibacter | 93.8 | 0.02289 | 0.0010 |
| 8717 | 4341010 | Bacteroidetes | Spirosoma | 87.9 | 0.04841 | 0.0010 |
| 107767 | 832257 | Bacteroidetes | Spirosoma | 89.6 | 0.01004 | 0.0010 |
| 104628 | 1130804 | Bacteroidetes | Spirosoma | 93.1 | 0.00627 | <0.0001 |
| 102884 | 1130804 | Bacteroidetes | Spirosoma | 91.2 | 0.00337 | <0.0001 |
| 105474 | 832257 | Bacteroidetes | Spirosoma | 90.0 | 0.00335 | 0.0010 |
| 129279 | 832257 | Bacteroidetes | Spirosoma | 92.2 | 0.00289 | 0.0009 |
| 122924 | 832257 | Bacteroidetes | Spirosoma | 90.8 | 0.00145 | 0.0307 |
| 18210 | 1076279 | Bacteroidetes | Spirosoma | 91.2 | 0.00143 | <0.0001 |
| 27257 | 1067178 | Bacteroidetes | Spirosoma | 91.0 | 0.00072 | 0.0009 |
|  |  |  |  |  |  |  |
| 57935 | 583949 | Chlamydiae | Neochlamydia | 86.9 | 0.00060 | 0.0030 |
| 34538 | 4326875 | Chlamydiae | Other Chlamydiales | 89.2 | 0.01796 | 0.0010 |
| 79611 | 4335830 | Chlamydiae | Other Chlamydiales | 85.0 | 0.01004 | 0.0010 |
| 15675 | 543064 | Chlamydiae | Other Chlamydiales | 83.6 | 0.00228 | 0.0001 |
| 124720 | 823226 | Chlamydiae | Other Chlamydiales | 80.8 | 0.00113 | 0.0307 |
| 81087 | 552935 | Chlamydiae | Other Chlamydiales | 88.5 | 0.00036 | 0.0307 |
| 38707 | 23644 | Chlamydiae | Other Parachlamydiaceae | 86.4 | 0.00969 | 0.0010 |
| 103378 | 3100657 | Chlamydiae | Other Parachlamydiaceae | 86.7 | 0.00513 | <0.0001 |
| 116725 | 23644 | Chlamydiae | Other Parachlamydiaceae | 85.0 | 0.00270 | 0.0307 |
| 50129 | 605842 | Chlamydiae | Other Parachlamydiaceae | 88.3 | 0.00096 | 0.0001 |
| 133581 | 136719 | Chlamydiae | Parachlamydia | 90.4 | 0.00856 | 0.0010 |
| 124480 | 2931 | Chlamydiae | Parachlamydia | 89.2 | 0.00359 | 0.0096 |
|  |  |  |  |  |  |  |
| 105802 | 112962 | Chloroflexi | Herpetosiphon | 91.3 | 0.00188 | 0.0030 |
| 41151 | 160289 | Chloroflexi | Ktedonobacter | 96.5 | 0.03991 | 0.0010 |
| 598 | 160354 | Chloroflexi | Ktedonobacter | 95.3 | 0.00281 | 0.0096 |
| 7284 | 310817 | Chloroflexi | Other Chloroflexi | 98.3 | 0.00205 | 0.0030 |
| 24163 | 247875 | Chloroflexi | Other Thermomicrobia | 97.8 | 0.00353 | 0.0003 |
|  |  |  |  |  |  |  |
| 20961 | 365101 | Deinococcus-Thermus | Deinococcus | 87.5 | 0.06219 | 0.0010 |
| 58137 | 786067 | Deinococcus-Thermus | Deinococcus | 92.8 | 0.00618 | <0.0001 |
| 41796 | 810918 | Deinococcus-Thermus | Deinococcus | 94.5 | 0.00060 | 0.0030 |
| 21830 | 191980 | Deinococcus-Thermus | Meiothermus | 98.0 | 0.00836 | 0.0010 |
| 6746 | 4470065 | Deinococcus-Thermus | Truepera | 98.3 | 0.00498 | 0.0096 |
|  |  |  |  |  |  |  |
| 58010 | 514182 | Firmicutes | Acidaminococcus | 90.4 | 0.00427 | <0.0001 |
| 124924 | 364935 | Firmicutes | Anaerococcus | 97.2 | 0.01434 | 0.0010 |
| 121193 | 17956 | Firmicutes | Bacillus | 93.0 | 0.03168 | 0.0010 |
| 29069 | 693231 | Firmicutes | Bacillus | 91.6 | 0.01123 | 0.0010 |
| 115841 | 220242 | Firmicutes | Clostridium | 98.8 | 0.00542 | <0.0001 |
| 70024 | 4456142 | Firmicutes | Clostridium | 98.5 | 0.00530 | <0.0001 |
| 120094 | 292299 | Firmicutes | Clostridium | 97.0 | 0.00214 | 0.0030 |
| 38447 | 2850749 | Firmicutes | Clostridium | 94.3 | 0.00211 | 0.0307 |
| 3049 | 1105696 | Firmicutes | Clostridium | 97.5 | 0.00128 | 0.0307 |
| 46975 | 4374194 | Firmicutes | Clostridium | 98.8 | 0.00096 | 0.0001 |
| 5984 | 30555324 | Firmicutes | Coprococcus | 98.8 | 0.00237 | 0.0003 |
| 108464 | 41281 | Firmicutes | Desmospora | 92.8 | 0.00477 | 0.0001 |
| 1824 | 258375 | Firmicutes | Dialister | 92.5 | 0.00060 | 0.0030 |
| 43791 | 4064550 | Firmicutes | Erysipelothrix | 92.7 | 0.00523 | 0.0010 |
| 20030 | 4093791 | Firmicutes | Eubacterium | 98.5 | 0.00188 | 0.0030 |
| 33878 | 523577 | Firmicutes | Gallicola | 97.0 | 0.00904 | 0.0010 |
| 69917 | 4345946 | Firmicutes | Gracilibacillus | 92.5 | 0.00225 | 0.0009 |
| 73112 | 1778275 | Firmicutes | Helcococcus | 93.3 | 0.00590 | <0.0001 |
| 112243 | 727447 | Firmicutes | Jeotgalicoccus | 93.0 | 0.00590 | <0.0001 |
| 28092 | 4305372 | Firmicutes | Lactobacillus | 91.1 | 0.02341 | 0.0010 |
| 103767 | 851794 | Firmicutes | Lactobacillus | 90.9 | 0.01071 | 0.0010 |
| 83581 | 749329 | Firmicutes | Lactobacillus | 92.0 | 0.00602 | <0.0001 |
| 7812 | 851794 | Firmicutes | Lactobacillus | 90.9 | 0.00412 | 0.0001 |
| 23547 | 3946926 | Firmicutes | Lactobacillus | 91.3 | 0.00171 | 0.0096 |
| 31893 | 3946926 | Firmicutes | Lactobacillus | 90.9 | 0.00171 | 0.0096 |
| 107480 | 4305372 | Firmicutes | Lactobacillus | 91.1 | 0.00149 | 0.0307 |
| 131399 | 291816 | Firmicutes | Lactobacillus | 92.3 | 0.00108 | <0.0001 |
| 243 | 830659 | Firmicutes | Lactococcus | 90.6 | 0.00179 | 0.0307 |
| 123734 | 811179 | Firmicutes | Lysinibacillus | 91.3 | 0.00203 | 0.0030 |
| 110937 | 149335 | Firmicutes | Mitsukella | 92.5 | 0.02019 | 0.0010 |
| 85305 | 820843 | Firmicutes | Mogibacterium | 97.5 | 0.00887 | <0.0001 |
| 117578 | 4359869 | Firmicutes | Oceanobacillus | 93.0 | 0.00381 | 0.0001 |
| 78110 | 1129362 | Firmicutes | Other Bacillaceae | 90.9 | 0.02370 | 0.0010 |
| 96978 | 1099445 | Firmicutes | Other Bacillaceae | 90.9 | 0.00574 | <0.0001 |
| 5263 | 330570 | Firmicutes | Other Bacillaceae | 90.2 | 0.00354 | <0.0001 |
| 117304 | 97903 | Firmicutes | Other Bacillaceae | 92.3 | 0.00341 | 0.0001 |
| 104983 | 864140 | Firmicutes | Other Bacillaceae | 89.7 | 0.00145 | 0.0307 |
| 126677 | 100100 | Firmicutes | Other Bacillaceae | 91.4 | 0.00128 | 0.0307 |
| 122425 | 3631784 | Firmicutes | Other Bacillales | 93.6 | 0.00270 | 0.0307 |
| 106189 | 4333285 | Firmicutes | Other Bacillales | 90.6 | 0.00245 | <0.0001 |
| 11309 | 366937 | Firmicutes | Other Bacilli | 92.0 | 0.00326 | 0.0030 |
| 28521 | 2234838 | Firmicutes | Other Clostridiaceae | 93.3 | 0.00211 | 0.0307 |
| 122470 | 4387453 | Firmicutes | Other Clostridiales | 92.8 | 0.06207 | 0.0010 |
| 36962 | 181111 | Firmicutes | Other Clostridiales | 98.8 | 0.02156 | 0.0010 |
| 7853 | 4409117 | Firmicutes | Other Clostridiales | 97.3 | 0.01444 | 0.0010 |
| 89413 | 534031 | Firmicutes | Other Clostridiales | 96.8 | 0.01263 | 0.0010 |
| 46627 | 302433 | Firmicutes | Other Clostridiales | 91.6 | 0.01242 | 0.0010 |
| 15018 | 628226 | Firmicutes | Other Clostridiales | 97.8 | 0.00886 | 0.0010 |
| 88819 | 4101780 | Firmicutes | Other Clostridiales | 92.6 | 0.00763 | 0.0010 |
| 11099 | 4430891 | Firmicutes | Other Clostridiales | 93.3 | 0.00708 | <0.0001 |
| 9228 | 271602 | Firmicutes | Other Clostridiales | 97.5 | 0.00225 | 0.0009 |
| 110819 | 312858 | Firmicutes | Other Clostridiales | 94.1 | 0.00143 | 0.0307 |
| 123644 | 3879985 | Firmicutes | Other Incertae_Sedis_XI | 93.8 | 0.01844 | 0.0010 |
| 110829 | 1086889 | Firmicutes | Other Incertae_Sedis_XI | 98.8 | 0.01487 | <0.0001 |
| 3596 | 1102643 | Firmicutes | Other Incertae_Sedis_XI | 96.5 | 0.01394 | 0.0010 |
| 113789 | 586378 | Firmicutes | Other Incertae_Sedis_XI | 97.3 | 0.00644 | <0.0001 |
| 42798 | 1007750 | Firmicutes | Other Incertae_Sedis_XI | 97.8 | 0.00256 | 0.0009 |
| 103436 | 75475 | Firmicutes | Other Incertae_Sedis_XI | 96.5 | 0.00211 | 0.0307 |
| 53255 | 75475 | Firmicutes | Other Incertae_Sedis_XI | 95.5 | 0.00128 | 0.0307 |
| 83170 | 854765 | Firmicutes | Other Incertae_Sedis_XI | 98.5 | 0.00060 | 0.0030 |
| 14294 | 352243 | Firmicutes | Other Lachnospiraceae | 98.8 | 0.02949 | 0.0010 |
| 18938 | 314095 | Firmicutes | Other Lachnospiraceae | 98.8 | 0.00998 | 0.0010 |
| 109879 | 2393022 | Firmicutes | Other Lachnospiraceae | 98.8 | 0.00855 | 0.0010 |
| 56758 | 707653 | Firmicutes | Other Lachnospiraceae | 98.0 | 0.00482 | <0.0001 |
| 94439 | 189045 | Firmicutes | Other Lachnospiraceae | 97.5 | 0.00323 | 0.0009 |
| 5131 | 192958 | Firmicutes | Other Lachnospiraceae | 98.8 | 0.00113 | 0.0307 |
| 74708 | 200014 | Firmicutes | Other Lactobacillales | 94.8 | 0.00381 | 0.0001 |
| 75761 | 178713 | Firmicutes | Other Ruminococcaceae | 98.5 | 0.01559 | 0.0010 |
| 96867 | 4312167 | Firmicutes | Other Thermoactinomycetaceae | 92.5 | 0.00193 | 0.0096 |
| 52239 | 41128 | Firmicutes | Other Veillonellaceae | 91.1 | 0.01568 | 0.0010 |
| 86344 | 820764 | Firmicutes | Other Veillonellaceae | 89.5 | 0.00671 | <0.0001 |
| 124335 | 820764 | Firmicutes | Other Veillonellaceae | 90.6 | 0.00060 | 0.0030 |
| 111325 | 1144449 | Firmicutes | Paenibacillus | 93.0 | 0.02224 | 0.0010 |
| 124251 | 4465538 | Firmicutes | Pediococcus | 92.0 | 0.03326 | 0.0010 |
| 130764 | 4465538 | Firmicutes | Pediococcus | 90.9 | 0.00425 | 0.0009 |
| 18792 | 4310024 | Firmicutes | Peptococcus | 88.3 | 0.03582 | 0.0010 |
| 129850 | 357495 | Firmicutes | Peptoniphilus | 98.0 | 0.00798 | 0.0010 |
| 77195 | 654307 | Firmicutes | Peptoniphilus | 98.8 | 0.00729 | <0.0001 |
| 95298 | 743263 | Firmicutes | Planifilum | 92.8 | 0.01043 | 0.0010 |
| 54819 | 4433272 | Firmicutes | Propionispira | 89.5 | 0.00299 | 0.0003 |
| 99122 | 793769 | Firmicutes | Pseudoramibacter | 98.3 | 0.00095 | 0.0096 |
| 82709 | 199640 | Firmicutes | Ruminococcus | 98.0 | 0.01767 | 0.0010 |
| 36648 | 4315471 | Firmicutes | Ruminococcus | 96.5 | 0.00649 | <0.0001 |
| 79998 | 906051 | Firmicutes | Rummeliibacillus | 93.0 | 0.00241 | 0.0030 |
| 40442 | 594278 | Firmicutes | Selenomonas | 91.3 | 0.01677 | 0.0010 |
| 38575 | 655531 | Firmicutes | Sporosarcina | 91.3 | 0.00375 | <0.0001 |
| 21976 | 2896107 | Firmicutes | Staphylococcus | 91.1 | 0.00444 | 0.0003 |
| 114885 | 675940 | Firmicutes | Staphylococcus | 91.3 | 0.00133 | 0.0307 |
| 67 | 802262 | Firmicutes | Streptococcus | 92.0 | 0.02472 | 0.0010 |
| 35037 | 1126851 | Firmicutes | Streptococcus | 92.5 | 0.00278 | 0.0009 |
| 125245 | 569215 | Firmicutes | Tepidanaerobacter | 98.8 | 0.01123 | 0.0010 |
| 47845 | 569215 | Firmicutes | Tepidanaerobacter | 94.3 | 0.00123 | 0.0307 |
| 39245 | 343273 | Firmicutes | Vagococcus | 91.6 | 0.00128 | 0.0307 |
| 111381 | 154506 | Firmicutes | Vagococcus | 91.1 | 0.00119 | 0.0307 |
| 119815 | 773541 | Firmicutes | Virgibacillus | 91.1 | 0.00107 | 0.0009 |
| 67819 | 4423978 | Firmicutes | Weissella | 91.6 | 0.01965 | 0.0010 |
| 89891 | 1126009 | Firmicutes | Weissella | 91.5 | 0.01394 | 0.0010 |
| 104658 | 64384 | Firmicutes | Weissella | 90.9 | 0.00499 | 0.0001 |
| 77701 | 299879 | Firmicutes | Weissella | 91.3 | 0.00438 | 0.0003 |
| 87222 | 299879 | Firmicutes | Weissella | 90.9 | 0.00413 | 0.0003 |
| 84399 | 64384 | Firmicutes | Weissella | 90.9 | 0.00365 | 0.0001 |
| 122981 | 299879 | Firmicutes | Weissella | 90.9 | 0.00340 | 0.0001 |
| 111481 | 64384 | Firmicutes | Weissella | 90.6 | 0.00314 | 0.0009 |
| 23762 | 64384 | Firmicutes | Weissella | 90.6 | 0.00233 | 0.0009 |
| 83237 | 299879 | Firmicutes | Weissella | 90.9 | 0.00212 | 0.0003 |
| 70650 | 299879 | Firmicutes | Weissella | 90.6 | 0.00179 | 0.0307 |
| 98943 | 64384 | Firmicutes | Weissella | 91.1 | 0.00149 | 0.0307 |
| 73970 | 64384 | Firmicutes | Weissella | 90.6 | 0.00121 | 0.0030 |
|  |  |  |  |  |  |  |
| 112870 | 532752 | Fusobacteria | Cetobacterium | 95.3 | 0.00801 | 0.0010 |
| 53142 | 941024 | Fusobacteria | Fusobacterium | 97.5 | 0.00128 | 0.0307 |
| 44125 | 31235 | Fusobacteria | Leptotrichia | 97.8 | 0.00469 | <0.0001 |
|  |  |  |  |  |  |  |
| 39440 | 217388 | Gemmatimonadetes | Gemmatimonas | 93.6 | 0.01275 | 0.0010 |
| 104638 | 114389 | Gemmatimonadetes | Gemmatimonas | 93.3 | 0.01186 | 0.0010 |
| 59955 | 1132137 | Gemmatimonadetes | Gemmatimonas | 91.9 | 0.00675 | <0.0001 |
| 7507 | 1044938 | Gemmatimonadetes | Gemmatimonas | 93.8 | 0.00492 | 0.0010 |
| 56365 | 1066717 | Gemmatimonadetes | Gemmatimonas | 94.3 | 0.00396 | 0.0010 |
| 18567 | 1132137 | Gemmatimonadetes | Gemmatimonas | 93.8 | 0.00241 | 0.0030 |
| 21064 | 403359 | Gemmatimonadetes | Gemmatimonas | 94.3 | 0.00239 | 0.0010 |
| 121730 | 779506 | Gemmatimonadetes | Gemmatimonas | 93.3 | 0.00229 | 0.0003 |
| 2855 | 1066717 | Gemmatimonadetes | Gemmatimonas | 92.1 | 0.00143 | <0.0001 |
| 7587 | 855996 | Gemmatimonadetes | Gemmatimonas | 92.4 | 0.00131 | <0.0001 |
| 77163 | 4319210 | Gemmatimonadetes | Gemmatimonas | 93.6 | 0.00072 | 0.0009 |
|  |  |  |  |  |  |  |
| 30096 | 4443105 | OD1 | Other OD1_genera_incertae_sedis | 92.1 | 0.03808 | 0.0010 |
| 360 | 16854 | OD1 | Other OD1_genera_incertae_sedis | 86.7 | 0.03607 | 0.0010 |
| 63838 | 4467411 | OD1 | Other OD1_genera_incertae_sedis | 94.8 | 0.02535 | 0.0010 |
| 9673 | 4467411 | OD1 | Other OD1_genera_incertae_sedis | 87.7 | 0.02343 | 0.0010 |
| 60882 | 65700 | OD1 | Other OD1_genera_incertae_sedis | 91.9 | 0.02225 | 0.0010 |
| 14833 | 515187 | OD1 | Other OD1_genera_incertae_sedis | 92.9 | 0.02054 | 0.0010 |
| 128508 | 4322208 | OD1 | Other OD1_genera_incertae_sedis | 87.4 | 0.02011 | 0.0010 |
| 14701 | 4316797 | OD1 | Other OD1_genera_incertae_sedis | 88.4 | 0.01928 | 0.0010 |
| 41483 | 143479 | OD1 | Other OD1_genera_incertae_sedis | 90.1 | 0.01882 | 0.0010 |
| 129454 | 521023 | OD1 | Other OD1_genera_incertae_sedis | 86.9 | 0.01575 | 0.0010 |
| 68576 | 16854 | OD1 | Other OD1_genera_incertae_sedis | 85.4 | 0.01124 | 0.0010 |
| 75221 | 4439808 | OD1 | Other OD1_genera_incertae_sedis | 91.6 | 0.00996 | 0.0001 |
| 16014 | 210146 | OD1 | Other OD1_genera_incertae_sedis | 89.1 | 0.00898 | 0.0010 |
| 118089 | 105625 | OD1 | Other OD1_genera_incertae_sedis | 85.0 | 0.00871 | 0.0003 |
| 102764 | 16854 | OD1 | Other OD1_genera_incertae_sedis | 88.4 | 0.00864 | <0.0001 |
| 18442 | 2881191 | OD1 | Other OD1_genera_incertae_sedis | 90.9 | 0.00683 | 0.0010 |
| 33294 | 4306563 | OD1 | Other OD1_genera_incertae_sedis | 94.6 | 0.00642 | 0.0010 |
| 125713 | 4467411 | OD1 | Other OD1_genera_incertae_sedis | 92.4 | 0.00632 | <0.0001 |
| 3278 | 4467411 | OD1 | Other OD1_genera_incertae_sedis | 86.2 | 0.00571 | <0.0001 |
| 10934 | 4444006 | OD1 | Other OD1_genera_incertae_sedis | 90.6 | 0.00561 | 0.0001 |
| 111266 | 4310797 | OD1 | Other OD1_genera_incertae_sedis | 85.5 | 0.00551 | 0.0010 |
| 111778 | 4322208 | OD1 | Other OD1_genera_incertae_sedis | 88.5 | 0.00543 | <0.0001 |
| 89054 | 4322208 | OD1 | Other OD1_genera_incertae_sedis | 85.5 | 0.00498 | 0.0010 |
| 22179 | 16854 | OD1 | Other OD1_genera_incertae_sedis | 86.2 | 0.00472 | 0.0001 |
| 99769 | 210146 | OD1 | Other OD1_genera_incertae_sedis | 85.7 | 0.00471 | <0.0001 |
| 84494 | 4467411 | OD1 | Other OD1_genera_incertae_sedis | 93.6 | 0.00459 | 0.0003 |
| 22833 | 4467411 | OD1 | Other OD1_genera_incertae_sedis | 93.6 | 0.00440 | <0.0001 |
| 86782 | 4443105 | OD1 | Other OD1_genera_incertae_sedis | 92.4 | 0.00428 | <0.0001 |
| 131774 | 521023 | OD1 | Other OD1_genera_incertae_sedis | 84.8 | 0.00428 | 0.0010 |
| 71155 | 4467411 | OD1 | Other OD1_genera_incertae_sedis | 86.2 | 0.00417 | 0.0003 |
| 120789 | 210146 | OD1 | Other OD1_genera_incertae_sedis | 87.4 | 0.00386 | 0.0001 |
| 114678 | 136227 | OD1 | Other OD1_genera_incertae_sedis | 92.1 | 0.00385 | <0.0001 |
| 132046 | 4380648 | OD1 | Other OD1_genera_incertae_sedis | 86.6 | 0.00361 | 0.0010 |
| 8474 | 678059 | OD1 | Other OD1_genera_incertae_sedis | 83.5 | 0.00359 | 0.0096 |
| 84471 | 4444006 | OD1 | Other OD1_genera_incertae_sedis | 89.9 | 0.00359 | 0.0096 |
| 97536 | 16854 | OD1 | Other OD1_genera_incertae_sedis | 85.9 | 0.00349 | 0.0010 |
| 32808 | 4322208 | OD1 | Other OD1_genera_incertae_sedis | 85.1 | 0.00342 | <0.0001 |
| 72188 | 4467411 | OD1 | Other OD1_genera_incertae_sedis | 87.0 | 0.00338 | <0.0001 |
| 5500 | 4322208 | OD1 | Other OD1_genera_incertae_sedis | 90.6 | 0.00321 | 0.0001 |
| 85653 | 179710 | OD1 | Other OD1_genera_incertae_sedis | 83.7 | 0.00321 | 0.0001 |
| 82718 | 4477111 | OD1 | Other OD1_genera_incertae_sedis | 93.1 | 0.00311 | 0.0009 |
| 82458 | 678059 | OD1 | Other OD1_genera_incertae_sedis | 91.4 | 0.00301 | 0.0010 |
| 86653 | 678059 | OD1 | Other OD1_genera_incertae_sedis | 86.0 | 0.00270 | 0.0307 |
| 49270 | 678059 | OD1 | Other OD1_genera_incertae_sedis | 90.4 | 0.00263 | 0.0003 |
| 78185 | 104773 | OD1 | Other OD1_genera_incertae_sedis | 86.8 | 0.00257 | 0.0009 |
| 100800 | 16854 | OD1 | Other OD1_genera_incertae_sedis | 89.1 | 0.00257 | 0.0009 |
| 120314 | 508877 | OD1 | Other OD1_genera_incertae_sedis | 86.7 | 0.00238 | 0.0030 |
| 56604 | 678059 | OD1 | Other OD1_genera_incertae_sedis | 91.1 | 0.00236 | 0.0096 |
| 79951 | 4467411 | OD1 | Other OD1_genera_incertae_sedis | 87.2 | 0.00228 | 0.0010 |
| 55366 | 4443105 | OD1 | Other OD1_genera_incertae_sedis | 92.9 | 0.00200 | 0.0003 |
| 113172 | 4375615 | OD1 | Other OD1_genera_incertae_sedis | 86.2 | 0.00200 | 0.0003 |
| 59389 | 1114884 | OD1 | Other OD1_genera_incertae_sedis | 94.3 | 0.00193 | 0.0030 |
| 49769 | 4467411 | OD1 | Other OD1_genera_incertae_sedis | 92.4 | 0.00189 | 0.0009 |
| 52061 | 3733463 | OD1 | Other OD1_genera_incertae_sedis | 86.2 | 0.00188 | 0.0030 |
| 65963 | 104773 | OD1 | Other OD1_genera_incertae_sedis | 84.1 | 0.00171 | 0.0096 |
| 67644 | 678059 | OD1 | Other OD1_genera_incertae_sedis | 92.8 | 0.00171 | 0.0009 |
| 10598 | 4427555 | OD1 | Other OD1_genera_incertae_sedis | 84.2 | 0.00161 | 0.0096 |
| 99365 | 4467411 | OD1 | Other OD1_genera_incertae_sedis | 85.9 | 0.00143 | 0.0307 |
| 128476 | 1132720 | OD1 | Other OD1_genera_incertae_sedis | 84.1 | 0.00143 | 0.0307 |
| 112859 | 4322208 | OD1 | Other OD1_genera_incertae_sedis | 85.5 | 0.00143 | 0.0030 |
| 96532 | 284266 | OD1 | Other OD1_genera_incertae_sedis | 96.0 | 0.00128 | 0.0307 |
| 85040 | 277716 | OD1 | Other OD1_genera_incertae_sedis | 87.7 | 0.00128 | 0.0307 |
| 67982 | 4322208 | OD1 | Other OD1_genera_incertae_sedis | 86.5 | 0.00120 | <0.0001 |
| 112151 | 678059 | OD1 | Other OD1_genera_incertae_sedis | 92.8 | 0.00114 | 0.0096 |
| 22920 | 678059 | OD1 | Other OD1_genera_incertae_sedis | 88.6 | 0.00072 | 0.0009 |
| 113088 | 4477111 | OD1 | Other OD1_genera_incertae_sedis | 88.9 | 0.00072 | 0.0009 |
|  |  |  |  |  |  |  |
| 36057 | 1719550 | Planctomycetes | Gemmata | 91.3 | 0.00370 | 0.0010 |
| 126936 | 2801322 | Planctomycetes | Other Planctomycetaceae | 93.9 | 0.10002 | 0.0010 |
| 21534 | 2339928 | Planctomycetes | Other Planctomycetaceae | 95.1 | 0.03231 | 0.0010 |
| 30883 | 252665 | Planctomycetes | Other Planctomycetaceae | 97.3 | 0.01924 | 0.0010 |
| 52049 | 254402 | Planctomycetes | Other Planctomycetaceae | 94.2 | 0.01796 | 0.0010 |
| 76270 | 4382182 | Planctomycetes | Other Planctomycetaceae | 93.9 | 0.01602 | 0.0010 |
| 118875 | 821747 | Planctomycetes | Other Planctomycetaceae | 91.5 | 0.00964 | 0.0010 |
| 46950 | 655207 | Planctomycetes | Other Planctomycetaceae | 92.7 | 0.00884 | 0.0010 |
| 81260 | 1132306 | Planctomycetes | Other Planctomycetaceae | 90.3 | 0.00531 | <0.0001 |
| 134277 | 214051 | Planctomycetes | Other Planctomycetaceae | 96.3 | 0.00513 | 0.0010 |
| 8926 | 206981 | Planctomycetes | Other Planctomycetaceae | 94.1 | 0.00428 | 0.0010 |
| 115573 | 350943 | Planctomycetes | Other Planctomycetaceae | 93.7 | 0.00402 | <0.0001 |
| 17248 | 244300 | Planctomycetes | Other Planctomycetaceae | 91.6 | 0.00394 | 0.0010 |
| 118769 | 807595 | Planctomycetes | Other Planctomycetaceae | 93.9 | 0.00371 | <0.0001 |
| 94519 | 563036 | Planctomycetes | Other Planctomycetaceae | 95.0 | 0.00314 | <0.0001 |
| 11013 | 655207 | Planctomycetes | Other Planctomycetaceae | 92.0 | 0.00228 | 0.0001 |
| 72436 | 3276016 | Planctomycetes | Other Planctomycetaceae | 96.6 | 0.00171 | 0.0009 |
| 123170 | 2193695 | Planctomycetes | Other Planctomycetaceae | 92.2 | 0.00156 | <0.0001 |
| 42681 | 279355 | Planctomycetes | Other Planctomycetaceae | 96.6 | 0.00152 | 0.0030 |
| 18428 | 2718809 | Planctomycetes | Other Planctomycetaceae | 86.5 | 0.00143 | <0.0001 |
| 112990 | 713582 | Planctomycetes | Other Planctomycetaceae | 88.9 | 0.00113 | 0.0307 |
| 67374 | 4382182 | Planctomycetes | Other Planctomycetaceae | 92.2 | 0.00108 | <0.0001 |
| 33222 | 4382182 | Planctomycetes | Other Planctomycetaceae | 91.4 | 0.00060 | 0.0030 |
| 78612 | 4382182 | Planctomycetes | Other Planctomycetaceae | 91.4 | 0.00048 | 0.0096 |
| 46361 | 4382182 | Planctomycetes | Other Planctomycetaceae | 92.2 | 0.00036 | 0.0307 |
| 12514 | 4437 | Planctomycetes | Pirellula | 92.6 | 0.00456 | 0.0010 |
| 111834 | 518697 | Planctomycetes | Planctomyces | 92.9 | 0.00150 | 0.0096 |
| 31296 | 267819 | Planctomycetes | Planctomyces | 90.4 | 0.00143 | 0.0030 |
| 23824 | 202033 | Planctomycetes | Singulisphaera | 91.9 | 0.01522 | 0.0010 |
| 89491 | 890463 | Planctomycetes | Singulisphaera | 93.3 | 0.01161 | 0.0010 |
| 6506 | 1060246 | Planctomycetes | Singulisphaera | 94.9 | 0.00778 | 0.0010 |
| 99690 | 972396 | Planctomycetes | Singulisphaera | 94.6 | 0.00556 | <0.0001 |
| 84700 | 227534 | Planctomycetes | Singulisphaera | 94.4 | 0.00485 | 0.0010 |
| 45719 | 1052451 | Planctomycetes | Singulisphaera | 92.9 | 0.00408 | 0.0010 |
| 64612 | 775313 | Planctomycetes | Singulisphaera | 95.1 | 0.00145 | 0.0307 |
| 68713 | 3837248 | Planctomycetes | Singulisphaera | 91.9 | 0.00048 | 0.0096 |
| 127871 | 962448 | Planctomycetes | Zavarzinella | 92.1 | 0.00855 | 0.0010 |
|  |  |  |  |  |  |  |
| 64349 | 209511 | Proteobacteria | Acinetobacter | 91.4 | 0.00666 | <0.0001 |
| 97188 | 360440 | Proteobacteria | Acinetobacter | 91.8 | 0.00300 | 0.0001 |
| 75882 | 831300 | Proteobacteria | Aeromonas | 91.3 | 0.00150 | 0.0096 |
| 95183 | 4466150 | Proteobacteria | Aggregatibacter | 93.0 | 0.00983 | <0.0001 |
| 131726 | 2881877 | Proteobacteria | Aquabacterium | 84.3 | 0.00434 | <0.0001 |
| 74817 | 320125 | Proteobacteria | Aquicella | 89.7 | 0.00476 | <0.0001 |
| 17798 | 574974 | Proteobacteria | Aquicella | 91.6 | 0.00201 | 0.0030 |
| 110573 | 333779 | Proteobacteria | Aquicella | 88.1 | 0.00168 | <0.0001 |
| 16547 | 714033 | Proteobacteria | Arcobacter | 97.3 | 0.00311 | 0.0001 |
| 104984 | 11162 | Proteobacteria | Arcobacter | 98.3 | 0.00241 | 0.0030 |
| 81467 | 662915 | Proteobacteria | Aurantimonas | 96.8 | 0.00596 | <0.0001 |
| 121336 | 2286322 | Proteobacteria | Bartonella | 97.0 | 0.03811 | 0.0010 |
| 51823 | 338371 | Proteobacteria | Bdellovibrio | 92.1 | 0.00964 | 0.0010 |
| 29593 | 972024 | Proteobacteria | Bdellovibrio | 96.5 | 0.00413 | 0.0003 |
| 111464 | 843663 | Proteobacteria | Beijerinckia | 97.8 | 0.00241 | 0.0030 |
| 125642 | 240075 | Proteobacteria | Brevundimonas | 97.5 | 0.00072 | 0.0009 |
| 111773 | 169006 | Proteobacteria | Brevundimonas | 98.5 | 0.00168 | <0.0001 |
| 37287 | 784002 | Proteobacteria | Curvibacter | 92.5 | 0.00510 | 0.0030 |
| 20623 | 570988 | Proteobacteria | Devosia | 97.3 | 0.00036 | 0.0307 |
| 73225 | 1125248 | Proteobacteria | Dokdonella | 92.5 | 0.00171 | 0.0009 |
| 64984 | 1060517 | Proteobacteria | Duganella | 91.4 | 0.00299 | 0.0003 |
| 43214 | 4480952 | Proteobacteria | Duganella | 83.8 | 0.00128 | 0.0307 |
| 36734 | 299851 | Proteobacteria | Escherichia/Shigella | 89.2 | 0.00087 | 0.0307 |
| 61349 | 581782 | Proteobacteria | Escherichia/Shigella | 89.9 | 0.00081 | 0.0307 |
| 89539 | 9870 | Proteobacteria | Escherichia/Shigella | 89.9 | 0.00067 | 0.0307 |
| 70410 | 4366499 | Proteobacteria | Geminicoccus | 98.5 | 0.00281 | 0.0096 |
| 36891 | 4456499 | Proteobacteria | Geminicoccus | 95.8 | 0.00150 | 0.0096 |
| 115443 | 1145138 | Proteobacteria | Hydrogenophaga | 92.5 | 0.00413 | 0.0003 |
| 8463 | 239537 | Proteobacteria | Hyphomicrobium | 98.0 | 0.01669 | 0.0010 |
| 86077 | 4371421 | Proteobacteria | Hyphomicrobium | 97.5 | 0.00385 | <0.0001 |
| 15853 | 974797 | Proteobacteria | Ideonella | 91.6 | 0.00555 | <0.0001 |
| 103399 | 4480775 | Proteobacteria | Kingella | 93.0 | 0.00666 | <0.0001 |
| 21198 | 315994 | Proteobacteria | Legionella | 92.0 | 0.01618 | <0.0001 |
| 69382 | 1120485 | Proteobacteria | Legionella | 92.3 | 0.01112 | 0.0010 |
| 30248 | 829266 | Proteobacteria | Lysobacter | 92.7 | 0.40503 | 0.0010 |
| 4298 | 4469864 | Proteobacteria | Lysobacter | 90.6 | 0.00621 | 0.0010 |
| 74776 | 4386631 | Proteobacteria | Methylibium | 91.3 | 0.02890 | 0.0010 |
| 67028 | 226906 | Proteobacteria | Methylibium | 92.5 | 0.01165 | 0.0010 |
| 75872 | 689696 | Proteobacteria | Methylibium | 92.0 | 0.00809 | 0.0010 |
| 99157 | 689696 | Proteobacteria | Methylibium | 91.6 | 0.00471 | <0.0001 |
| 114145 | 226906 | Proteobacteria | Methylibium | 93.2 | 0.00072 | 0.0009 |
| 4743 | 4314749 | Proteobacteria | Neisseria | 83.8 | 0.00675 | 0.0010 |
| 57323 | 1106060 | Proteobacteria | Neisseria | 89.2 | 0.00211 | 0.0307 |
| 116577 | 4480022 | Proteobacteria | Novosphingobium | 96.5 | 0.00036 | 0.0307 |
| 94696 | 811982 | Proteobacteria | Oceanisphaera | 92.3 | 0.00214 | 0.0030 |
| 133086 | 838788 | Proteobacteria | Other Acetobacteriaceae | 98.3 | 0.03178 | 0.0010 |
| 31812 | 407742 | Proteobacteria | Other Acetobacteriaceae | 96.8 | 0.01665 | 0.0010 |
| 37943 | 532895 | Proteobacteria | Other Acetobacteriaceae | 98.3 | 0.01394 | 0.0010 |
| 82880 | 4459144 | Proteobacteria | Other Acetobacteriaceae | 97.0 | 0.01226 | 0.0010 |
| 70866 | 831282 | Proteobacteria | Other Acetobacteriaceae | 95.3 | 0.01076 | 0.0010 |
| 52179 | 360826 | Proteobacteria | Other Acetobacteriaceae | 97.3 | 0.01028 | 0.0010 |
| 87252 | 838678 | Proteobacteria | Other Acetobacteriaceae | 97.3 | 0.01003 | 0.0010 |
| 610 | 841979 | Proteobacteria | Other Acetobacteriaceae | 97.5 | 0.00434 | <0.0001 |
| 47780 | 815314 | Proteobacteria | Other Acetobacteriaceae | 96.3 | 0.00402 | 0.0010 |
| 40522 | 845577 | Proteobacteria | Other Acetobacteriaceae | 96.0 | 0.00257 | 0.0009 |
| 59105 | 815314 | Proteobacteria | Other Acetobacteriaceae | 97.5 | 0.00211 | 0.0307 |
| 26803 | 216023 | Proteobacteria | Other Acetobacteriaceae | 96.3 | 0.00167 | <0.0001 |
| 133652 | 360826 | Proteobacteria | Other Acetobacteriaceae | 96.3 | 0.00156 | <0.0001 |
| 28669 | 361359 | Proteobacteria | Other Acetobacteriaceae | 97.0 | 0.00143 | <0.0001 |
| 61523 | 360500 | Proteobacteria | Other Acetobacteriaceae | 98.3 | 0.00143 | <0.0001 |
| 31742 | 221365 | Proteobacteria | Other Acetobacteriaceae | 95.8 | 0.00096 | 0.0001 |
| 4548 | 361359 | Proteobacteria | Other Acetobacteriaceae | 96.8 | 0.00048 | 0.0096 |
| 15305 | 221365 | Proteobacteria | Other Acetobacteriaceae | 96.0 | 0.00036 | 0.0307 |
| 54513 | 815116 | Proteobacteria | Other Acetobacteriaceae | 94.8 | 0.00036 | 0.0307 |
| 98675 | 360826 | Proteobacteria | Other Acetobacteriaceae | 96.8 | 0.00036 | 0.0307 |
| 34543 | 2679706 | Proteobacteria | Other Alphaproteobacteria | 98.8 | 0.01368 | 0.0010 |
| 18436 | 4476892 | Proteobacteria | Other Alphaproteobacteria | 96.5 | 0.00793 | 0.0010 |
| 24722 | 101800 | Proteobacteria | Other Alphaproteobacteria | 93.8 | 0.00556 | <0.0001 |
| 88120 | 1045198 | Proteobacteria | Other Alphaproteobacteria | 98.5 | 0.00289 | 0.0009 |
| 36696 | 579026 | Proteobacteria | Other Alphaproteobacteria | 97.8 | 0.00200 | 0.0003 |
| 64737 | 248953 | Proteobacteria | Other Alphaproteobacteria | 94.3 | 0.00131 | <0.0001 |
| 19291 | 4396338 | Proteobacteria | Other Alphaproteobacteria | 89.8 | 0.00128 | 0.0307 |
| 117002 | 709657 | Proteobacteria | Other Alphaproteobacteria | 97.0 | 0.00126 | 0.0030 |
| 11674 | 1054800 | Proteobacteria | Other Alphaproteobacteria | 93.5 | 0.00036 | 0.0307 |
| 127397 | 1104923 | Proteobacteria | Other Betaproteobacteria | 92.0 | 0.04774 | 0.0010 |
| 98014 | 226599 | Proteobacteria | Other Betaproteobacteria | 90.9 | 0.03103 | 0.0010 |
| 19345 | 226599 | Proteobacteria | Other Betaproteobacteria | 91.6 | 0.00840 | 0.0010 |
| 97976 | 313830 | Proteobacteria | Other Betaproteobacteria | 89.7 | 0.00798 | 0.0010 |
| 39659 | 431910 | Proteobacteria | Other Betaproteobacteria | 91.3 | 0.00428 | <0.0001 |
| 98931 | 4091428 | Proteobacteria | Other Betaproteobacteria | 91.3 | 0.00384 | <0.0001 |
| 23251 | 279993 | Proteobacteria | Other Betaproteobacteria | 92.7 | 0.00314 | <0.0001 |
| 23300 | 1085127 | Proteobacteria | Other Betaproteobacteria | 92.3 | 0.00200 | 0.0003 |
| 46892 | 676066 | Proteobacteria | Other Bradyrhizobiaceae | 97.8 | 0.03003 | 0.0010 |
| 70504 | 4386631 | Proteobacteria | Other Burkholderiales | 91.1 | 0.00288 | 0.0010 |
| 42139 | 4386631 | Proteobacteria | Other Burkholderiales | 90.9 | 0.00276 | <0.0001 |
| 70726 | 61836 | Proteobacteria | Other Burkholderiales | 91.3 | 0.00236 | 0.0096 |
| 133298 | 4386631 | Proteobacteria | Other Burkholderiales | 90.6 | 0.00108 | 0.0307 |
| 61362 | 226906 | Proteobacteria | Other Burkholderiales_incertae_sedis | 90.9 | 0.00264 | 0.0010 |
| 64638 | 4386631 | Proteobacteria | Other Burkholderiales_incertae_sedis | 91.1 | 0.00216 | <0.0001 |
| 35474 | 1044235 | Proteobacteria | Other Caulobacteriaceae | 98.3 | 0.00036 | 0.0307 |
| 27706 | 119515 | Proteobacteria | Other Comamonadaceae | 90.4 | 0.05087 | 0.0010 |
| 8373 | 119515 | Proteobacteria | Other Comamonadaceae | 90.6 | 0.03574 | 0.0010 |
| 41441 | 838837 | Proteobacteria | Other Comamonadaceae | 91.1 | 0.01326 | 0.0010 |
| 58225 | 119515 | Proteobacteria | Other Comamonadaceae | 89.9 | 0.00144 | 0.0009 |
| 21471 | 574721 | Proteobacteria | Other Comamonadaceae | 90.6 | 0.00132 | 0.0030 |
| 121886 | 758877 | Proteobacteria | Other Comamonadaceae | 90.6 | 0.00096 | 0.0001 |
| 5958 | 119515 | Proteobacteria | Other Comamonadaceae | 90.2 | 0.00036 | 0.0307 |
| 93889 | 119515 | Proteobacteria | Other Comamonadaceae | 90.6 | 0.00036 | 0.0307 |
| 120443 | 38267 | Proteobacteria | Other Comamonadaceae | 91.3 | 0.00036 | 0.0307 |
| 90085 | 4416974 | Proteobacteria | Other Deltaproteobacteria | 92.3 | 0.02936 | 0.0010 |
| 91327 | 822250 | Proteobacteria | Other Deltaproteobacteria | 92.3 | 0.01397 | 0.0010 |
| 72788 | 2183856 | Proteobacteria | Other Deltaproteobacteria | 90.7 | 0.01112 | 0.0010 |
| 108197 | 236923 | Proteobacteria | Other Deltaproteobacteria | 90.4 | 0.00428 | <0.0001 |
| 122888 | 51583 | Proteobacteria | Other Deltaproteobacteria | 90.6 | 0.00383 | 0.0010 |
| 16960 | 1114189 | Proteobacteria | Other Deltaproteobacteria | 91.6 | 0.00200 | 0.0003 |
| 80446 | 738432 | Proteobacteria | Other Deltaproteobacteria | 90.9 | 0.00128 | 0.0307 |
| 126501 | 91557 | Proteobacteria | Other Enterobacteriaceae | 91.6 | 0.00413 | 0.0003 |
| 11032 | 9918 | Proteobacteria | Other Enterobacteriaceae | 91.1 | 0.00196 | 0.0096 |
| 112908 | 1134377 | Proteobacteria | Other Enterobacteriaceae | 91.1 | 0.00111 | 0.0307 |
| 53607 | 218419 | Proteobacteria | Other Erythrobacteriaceae | 98.8 | 0.00193 | 0.0096 |
| 71093 | 279180 | Proteobacteria | Other Gammaproteobacteria | 94.5 | 0.02871 | 0.0010 |
| 71814 | 204786 | Proteobacteria | Other Gammaproteobacteria | 92.7 | 0.01795 | 0.0010 |
| 120006 | 2144521 | Proteobacteria | Other Gammaproteobacteria | 90.4 | 0.01454 | 0.0010 |
| 124912 | 1111525 | Proteobacteria | Other Gammaproteobacteria | 86.2 | 0.01067 | 0.0010 |
| 75881 | 581900 | Proteobacteria | Other Gammaproteobacteria | 92.0 | 0.00950 | 0.0010 |
| 22952 | 237001 | Proteobacteria | Other Gammaproteobacteria | 89.9 | 0.00386 | 0.0001 |
| 15000 | 753656 | Proteobacteria | Other Gammaproteobacteria | 90.6 | 0.00241 | 0.0030 |
| 12272 | 622861 | Proteobacteria | Other Gammaproteobacteria | 91.8 | 0.00214 | 0.0030 |
| 103396 | 4468101 | Proteobacteria | Other Gammaproteobacteria | 92.3 | 0.00086 | 0.0307 |
| 19099 | 524595 | Proteobacteria | Other Gammaproteobacteria | 91.8 | 0.00048 | 0.0096 |
| 100609 | 204335 | Proteobacteria | Other Hyphomicrobiaceae | 98.3 | 0.02138 | 0.0010 |
| 132180 | 240029 | Proteobacteria | Other Legionellaceae | 89.7 | 0.00362 | <0.0001 |
| 48888 | 260335 | Proteobacteria | Other Myxococcales | 91.3 | 0.00490 | 0.0010 |
| 91703 | 872600 | Proteobacteria | Other Myxococcales | 92.0 | 0.00193 | 0.0096 |
| 28760 | 4353904 | Proteobacteria | Other Myxococcales | 91.1 | 0.00171 | 0.0009 |
| 2335 | 113261 | Proteobacteria | Other Myxococcales | 91.3 | 0.00048 | 0.0096 |
| 93489 | 147685 | Proteobacteria | Other Oxalobacteraceae | 84.3 | 0.04779 | 0.0010 |
| 92686 | 4418009 | Proteobacteria | Other Oxalobacteraceae | 91.6 | 0.01806 | 0.0010 |
| 23715 | 548434 | Proteobacteria | Other Oxalobacteraceae | 91.6 | 0.00413 | 0.0003 |
| 122821 | 823479 | Proteobacteria | Other Oxalobacteraceae | 82.2 | 0.00256 | 0.0009 |
| 130352 | 137451 | Proteobacteria | Other Oxalobacteraceae | 92.3 | 0.00072 | 0.0009 |
| 22611 | 832215 | Proteobacteria | Other Polyangiaceae | 89.9 | 0.00192 | 0.0010 |
| 85357 | 912160 | Proteobacteria | Other Proteobacteria | 92.5 | 0.01362 | 0.0010 |
| 125325 | 509440 | Proteobacteria | Other Proteobacteria | 91.6 | 0.00899 | <0.0001 |
| 93580 | 241214 | Proteobacteria | Other Proteobacteria | 91.8 | 0.00482 | <0.0001 |
| 67956 | 3862614 | Proteobacteria | Other Proteobacteria | 86.9 | 0.00381 | 0.0001 |
| 79023 | 156878 | Proteobacteria | Other Proteobacteria | 87.4 | 0.00333 | 0.0003 |
| 15815 | 3222178 | Proteobacteria | Other Proteobacteria | 90.9 | 0.00295 | 0.0030 |
| 11700 | 509440 | Proteobacteria | Other Proteobacteria | 91.6 | 0.00285 | <0.0001 |
| 125636 | 3927146 | Proteobacteria | Other Proteobacteria | 82.3 | 0.00238 | 0.0030 |
| 123319 | 816965 | Proteobacteria | Other Proteobacteria | 92.8 | 0.00084 | 0.0003 |
| 19613 | 1117661 | Proteobacteria | Other Proteobacteria | 84.6 | 0.00060 | 0.0030 |
| 85309 | 1004427 | Proteobacteria | Other Proteobacteria | 89.4 | 0.00060 | 0.0030 |
| 26749 | 198655 | Proteobacteria | Other Rhizobiales | 97.0 | 0.49028 | 0.0010 |
| 112182 | 101542 | Proteobacteria | Other Rhizobiales | 98.3 | 0.12324 | 0.0010 |
| 102637 | 4402900 | Proteobacteria | Other Rhizobiales | 98.5 | 0.02199 | 0.0010 |
| 107565 | 806726 | Proteobacteria | Other Rhizobiales | 96.0 | 0.01829 | 0.0010 |
| 118804 | 951794 | Proteobacteria | Other Rhizobiales | 97.3 | 0.01235 | 0.0010 |
| 54005 | 558957 | Proteobacteria | Other Rhizobiales | 94.8 | 0.01151 | 0.0010 |
| 20240 | 162188 | Proteobacteria | Other Rhizobiales | 97.5 | 0.00896 | 0.0010 |
| 128576 | 3984866 | Proteobacteria | Other Rhizobiales | 96.8 | 0.00428 | 0.0010 |
| 123477 | 253167 | Proteobacteria | Other Rhizobiales | 96.3 | 0.00413 | 0.0003 |
| 5598 | 205156 | Proteobacteria | Other Rhizobiales | 96.8 | 0.00337 | 0.0003 |
| 132047 | 815975 | Proteobacteria | Other Rhizobiales | 98.0 | 0.00337 | 0.0010 |
| 76946 | 566212 | Proteobacteria | Other Rhizobiales | 97.3 | 0.00323 | 0.0010 |
| 32502 | 557145 | Proteobacteria | Other Rhizobiales | 98.3 | 0.00285 | <0.0001 |
| 51811 | 614357 | Proteobacteria | Other Rhizobiales | 95.0 | 0.00238 | 0.0030 |
| 134544 | 230258 | Proteobacteria | Other Rhodobacteraceae | 91.9 | 0.05334 | 0.0010 |
| 15131 | 80289 | Proteobacteria | Other Rhodobacteraceae | 96.5 | 0.00375 | <0.0001 |
| 86951 | 948119 | Proteobacteria | Other Rhodocyclaceae | 92.5 | 0.00655 | <0.0001 |
| 48963 | 1125639 | Proteobacteria | Other Rhodocyclaceae | 91.3 | 0.00225 | 0.0009 |
| 126515 | 1142584 | Proteobacteria | Other Rhodospirillales | 98.5 | 0.02993 | 0.0010 |
| 76367 | 822913 | Proteobacteria | Other Rhodospirillales | 95.3 | 0.00427 | <0.0001 |
| 3820 | 4253643 | Proteobacteria | Other Rhodospirillales | 95.0 | 0.00143 | 0.0030 |
| 68398 | 4398656 | Proteobacteria | Other Rhodospirillales | 97.0 | 0.00086 | 0.0307 |
| 32641 | 822853 | Proteobacteria | Other Sphingomonadaceae | 98.8 | 0.23594 | 0.0010 |
| 120668 | 367995 | Proteobacteria | Other Sphingomonadaceae | 97.3 | 0.02695 | 0.0010 |
| 52609 | 164830 | Proteobacteria | Other Sphingomonadaceae | 96.3 | 0.01328 | 0.0010 |
| 102085 | 4447334 | Proteobacteria | Other Sphingomonadaceae | 98.3 | 0.01212 | 0.0010 |
| 110861 | 4480022 | Proteobacteria | Other Sphingomonadaceae | 96.5 | 0.00624 | 0.0010 |
| 28558 | 1135077 | Proteobacteria | Other Sphingomonadaceae | 98.8 | 0.00358 | 0.0010 |
| 109530 | 4480022 | Proteobacteria | Other Sphingomonadaceae | 96.5 | 0.00131 | <0.0001 |
| 72156 | 836331 | Proteobacteria | Other Sphingomonadaceae | 96.0 | 0.00048 | 0.0096 |
| 85349 | 519510 | Proteobacteria | Other Sphingomonadales | 98.0 | 0.00940 | 0.0010 |
| 11817 | 982582 | Proteobacteria | Other Xanthomonadaceae | 92.5 | 0.00996 | 0.0010 |
| 91045 | 105699 | Proteobacteria | Other Xanthomonadaceae | 89.0 | 0.00539 | 0.0009 |
| 99407 | 506057 | Proteobacteria | Other Xanthomonadaceae | 91.8 | 0.00418 | 0.0010 |
| 131580 | 4455895 | Proteobacteria | Pantoea | 91.8 | 0.01540 | 0.0010 |
| 80113 | 112891 | Proteobacteria | Parasutterella | 93.0 | 0.00518 | <0.0001 |
| 31683 | 61836 | Proteobacteria | Pelomonas | 84.5 | 0.01636 | 0.0010 |
| 110572 | 185100 | Proteobacteria | Peredibacter | 90.7 | 0.00144 | <0.0001 |
| 112242 | 1088618 | Proteobacteria | Phenylobacterium | 98.3 | 0.00241 | 0.0030 |
| 19553 | 765388 | Proteobacteria | Phenylobacterium | 95.5 | 0.00161 | 0.0096 |
| 51758 | 757089 | Proteobacteria | Phenylobacterium | 97.8 | 0.00036 | 0.0307 |
| 22807 | 4452489 | Proteobacteria | Photobacterium | 91.6 | 0.00150 | 0.0096 |
| 96652 | 5158 | Proteobacteria | Phyllobacterium | 96.3 | 0.00233 | 0.0030 |
| 64358 | 345333 | Proteobacteria | Phyllobacterium | 96.5 | 0.00225 | 0.0096 |
| 115591 | 345333 | Proteobacteria | Phyllobacterium | 97.0 | 0.00221 | 0.0030 |
| 83321 | 4410974 | Proteobacteria | Polaromonas | 91.6 | 0.00155 | <0.0001 |
| 5810 | 4315605 | Proteobacteria | Porphyromonas | 94.1 | 0.00402 | <0.0001 |
| 87855 | 634948 | Proteobacteria | Pseudidiomarina | 91.1 | 0.00177 | 0.0307 |
| 79822 | 202466 | Proteobacteria | Pseudomonas | 90.6 | 0.00224 | 0.0307 |
| 79412 | 541223 | Proteobacteria | Pseudomonas | 91.3 | 0.00179 | 0.0096 |
| 82507 | 557974 | Proteobacteria | Pseudomonas | 91.1 | 0.00140 | 0.0307 |
| 124468 | 1566691 | Proteobacteria | Pseudomonas | 90.9 | 0.00083 | 0.0307 |
| 19993 | 499198 | Proteobacteria | Ralstonia | 92.5 | 0.00578 | <0.0001 |
| 64572 | 543416 | Proteobacteria | Rhodobacter | 95.8 | 0.00145 | 0.0307 |
| 19411 | 882616 | Proteobacteria | Roseomonas | 98.8 | 0.01446 | 0.0010 |
| 85259 | 740323 | Proteobacteria | Roseomonas | 95.3 | 0.00800 | 0.0010 |
| 94793 | 648004 | Proteobacteria | Roseomonas | 98.3 | 0.00333 | 0.0003 |
| 94747 | 805561 | Proteobacteria | Shewanella | 91.1 | 0.20072 | 0.0010 |
| 51625 | 575480 | Proteobacteria | Shewanella | 90.9 | 0.01180 | 0.0010 |
| 9154 | 814252 | Proteobacteria | Shewanella | 89.9 | 0.00421 | 0.0009 |
| 33597 | 4434188 | Proteobacteria | Simplicispira | 92.7 | 0.00490 | 0.0009 |
| 56400 | 820713 | Proteobacteria | Sphingomonas | 97.0 | 0.00526 | 0.0010 |
| 116348 | 836331 | Proteobacteria | Sphingomonas | 96.0 | 0.00502 | 0.0010 |
| 114129 | 836331 | Proteobacteria | Sphingomonas | 97.0 | 0.00239 | 0.0010 |
| 89370 | 820713 | Proteobacteria | Sphingomonas | 96.8 | 0.00191 | 0.0010 |
| 64444 | 820713 | Proteobacteria | Sphingomonas | 96.8 | 0.00096 | 0.0001 |
| 41739 | 627479 | Proteobacteria | Sphingomonas | 96.8 | 0.00072 | 0.0307 |
| 128272 | 217266 | Proteobacteria | Sphingomonas | 96.0 | 0.00048 | 0.0096 |
| 8629 | 820713 | Proteobacteria | Sphingomonas | 96.5 | 0.00036 | 0.0307 |
| 33157 | 820713 | Proteobacteria | Sphingomonas | 96.8 | 0.00036 | 0.0307 |
| 52004 | 820713 | Proteobacteria | Sphingomonas | 95.3 | 0.00036 | 0.0307 |
| 15832 | 4419310 | Proteobacteria | Starkeya | 97.5 | 0.00060 | 0.0030 |
| 60925 | 817926 | Proteobacteria | Stenotrophomonas | 91.4 | 0.00163 | 0.0096 |
| 84725 | 333380 | Proteobacteria | Sutterella | 91.1 | 0.00944 | 0.0010 |
| 48879 | 546567 | Proteobacteria | Tepidimonas | 92.0 | 0.00684 | 0.0010 |
| 111710 | 808818 | Proteobacteria | Thiovirga | 92.3 | 0.00385 | <0.0001 |
| 5612 | 1145927 | Proteobacteria | Wohlfartiimonas | 88.6 | 0.02672 | 0.0010 |
|  |  |  |  |  |  |  |
| 11093 | 3907 | Spirochaetes | Treponema | 92.8 | 0.01189 | 0.0010 |
|  |  |  |  |  |  |  |
| 58110 | 4400869 | SR1 | Other SR1_genera_incertae_sedis | 98.5 | 0.00357 | 0.0009 |
| 122995 | 346610 | SR1 | Other SR1_genera_incertae_sedis | 94.8 | 0.01027 | 0.0010 |
|  |  |  |  |  |  |  |
| 2967 | 302106 | Synergistes | Pyramidobacter | 90.6 | 0.00871 | 0.0003 |
|  |  |  |  |  |  |  |
| 106138 | 594001 | Tenericutes | Mycoplasma | 93.0 | 0.00510 | 0.0010 |
|  |  |  |  |  |  |  |
| 29222 | 1118721 | TM7 | Other TM7_genera_incertae_sedis | 94.0 | 0.00502 | 0.0010 |
| 41913 | 279572 | TM7 | Other TM7_genera_incertae_sedis | 94.8 | 0.00108 | 0.0009 |
| 42372 | 580909 | TM7 | Other TM7_genera_incertae_sedis | 87.2 | 0.00114 | 0.0096 |
| 50495 | 245302 | TM7 | Other TM7_genera_incertae_sedis | 97.5 | 0.00337 | 0.0003 |
| 60621 | 32738 | TM7 | Other TM7_genera_incertae_sedis | 95.5 | 0.00171 | 0.0096 |
| 69226 | 101901 | TM7 | Other TM7_genera_incertae_sedis | 97.5 | 0.00649 | <0.0001 |
| 82930 | 222592 | TM7 | Other TM7_genera_incertae_sedis | 91.8 | 0.01180 | 0.0010 |
| 84847 | 107878 | TM7 | Other TM7_genera_incertae_sedis | 95.3 | 0.00941 | 0.0010 |
| 92023 | 169160 | TM7 | Other TM7_genera_incertae_sedis | 92.3 | 0.00200 | 0.0003 |
| 97464 | 580909 | TM7 | Other TM7_genera_incertae_sedis | 93.3 | 0.00253 | 0.0010 |
| 99704 | 4472518 | TM7 | Other TM7_genera_incertae_sedis | 90.6 | 0.00086 | 0.0307 |
| 100676 | 65695 | TM7 | Other TM7_genera_incertae_sedis | 89.8 | 0.00115 | 0.0003 |
| 110853 | 333899 | TM7 | Other TM7_genera_incertae_sedis | 93.5 | 0.00951 | 0.0010 |
| 111811 | 220302 | TM7 | Other TM7_genera_incertae_sedis | 90.6 | 0.00770 | 0.0010 |
| 112030 | 101901 | TM7 | Other TM7_genera_incertae_sedis | 95.0 | 0.00285 | 0.0009 |
| 130289 | 693124 | TM7 | Other TM7_genera_incertae_sedis | 91.3 | 0.01583 | 0.0010 |
| 3608 | 4377584 | TM7 | Other TM7_genera_incertae_sedis | 91.6 | 0.00036 | 0.0307 |
| 22146 | 4332061 | TM7 | Other TM7_genera_incertae_sedis | 97.3 | 0.00060 | 0.0030 |
| 23718 | 4472518 | TM7 | Other TM7_genera_incertae_sedis | 92.1 | 0.00442 | <0.0001 |
|  |  |  |  |  |  |  |
| 49136 | 4458675 | Verrucomicrobia | Luteolibacter | 92.5 | 0.00402 | <0.0001 |
| 79441 | 313004 | Verrucomicrobia | Other Spartobacteria_genera_incertae_sedis | 92.5 | 0.07245 | 0.0010 |
| 19465 | 224834 | Verrucomicrobia | Other Spartobacteria_genera_incertae_sedis | 92.7 | 0.04590 | 0.0010 |
| 59684 | 630306 | Verrucomicrobia | Other Spartobacteria_genera_incertae_sedis | 92.0 | 0.01767 | 0.0010 |
| 51205 | 544067 | Verrucomicrobia | Other Spartobacteria_genera_incertae_sedis | 92.0 | 0.01392 | 0.0010 |
| 84627 | 2316426 | Verrucomicrobia | Other Spartobacteria_genera_incertae_sedis | 92.0 | 0.01107 | 0.0010 |
| 36797 | 220629 | Verrucomicrobia | Other Spartobacteria_genera_incertae_sedis | 91.6 | 0.01045 | 0.0010 |
| 88044 | 104216 | Verrucomicrobia | Other Spartobacteria_genera_incertae_sedis | 92.0 | 0.00627 | 0.0010 |
| 27379 | 1107011 | Verrucomicrobia | Other Spartobacteria_genera_incertae_sedis | 89.0 | 0.00517 | 0.0010 |
| 10869 | 223724 | Verrucomicrobia | Other Spartobacteria_genera_incertae_sedis | 92.7 | 0.00513 | 0.0010 |
| 9789 | 946250 | Verrucomicrobia | Other Spartobacteria_genera_incertae_sedis | 91.5 | 0.00494 | <0.0001 |
| 75035 | 1115406 | Verrucomicrobia | Other Spartobacteria_genera_incertae_sedis | 92.7 | 0.00257 | 0.0009 |
| 100324 | 1009340 | Verrucomicrobia | Other Spartobacteria_genera_incertae_sedis | 92.7 | 0.00216 | <0.0001 |
| 86666 | 929699 | Verrucomicrobia | Other Spartobacteria_genera_incertae_sedis | 92.0 | 0.00125 | 0.0307 |
| 99262 | 223724 | Verrucomicrobia | Other Spartobacteria_genera_incertae_sedis | 92.5 | 0.00114 | 0.0096 |
| 6762 | 225632 | Verrucomicrobia | Other Subdivision3_genera_incertae_sedis | 92.0 | 0.00822 | 0.0010 |
| 26548 | 4415674 | Verrucomicrobia | Other Verrucomicrobia | 86.4 | 0.05184 | 0.0010 |
| 17865 | 3426090 | Verrucomicrobia | Other Verrucomicrobiaceae | 91.6 | 0.00238 | 0.0096 |
| 96274 | 221896 | Verrucomicrobia | Spartobacteria | 97.5 | 0.00257 | <0.0001 |
| 121827 | 908868 | Verrucomicrobia | Verrucomicrobium | 94.9 | 0.00349 | 0.0010 |
| 127988 | 908868 | Verrucomicrobia | Verrucomicrobium | 91.2 | 0.00108 | <0.0001 |

^1^ unique number assigned to sequences with less than 97% similarity to other sequences

^2^ number that is associated with the sequence in the aligned Greengenes database that fits the query sequence

^3^ percent identity of the query sequence to the assigned Greengenes reference sequence

**Table S6: Full list of Operational Taxonomic Units (OTUs) found uniquely in the distal intestine of seawater Atlantic salmon, as identified by Metastats analysis.**

| **denovo ID^1^** | **Greengenes ID^2^** | **Phylum** | **Taxonomic assignment** | **Blast %** | **mean % SW** | **pvalue** |
| --- | --- | --- | --- | --- | --- | --- |
| 92888 | 1925459 | Acidobacteria | Other Gp6 | 92.5 | 0.00162 | 0.0218 |
|  |  |  |  |  |  |  |
| 56814 | 960871 | Actinobacteria | Actinomyces | 94.7 | 0.00270 | 0.0120 |
| 67248 | 140401 | Actinobacteria | Aeromicrobium | 95.8 | 0.00148 | 0.0236 |
| 89794 | 4407603 | Actinobacteria | Bifidobacterium | 93.4 | 0.01787 | 0.0010 |
| 26244 | 1134896 | Actinobacteria | Brevibacterium | 96.8 | 0.00992 | 0.0010 |
| 7259 | 759855 | Actinobacteria | Other Actinomycetales | 97.3 | 0.00173 | 0.0131 |
| 30596 | 2985422 | Actinobacteria | Other Bifidobacteriaceae | 97.5 | 0.01109 | 0.0010 |
| 74321 | 4383820 | Actinobacteria | Other Microbacteriaceae | 95.8 | 0.00443 | 0.0070 |
| 22011 | 271621 | Actinobacteria | Other Micrococcaceae | 95.1 | 0.00459 | 0.0080 |
| 66070 | 689950 | Actinobacteria | Rhodococcus | 97.5 | 0.00339 | 0.0010 |
|  |  |  |  |  |  |  |
| 6159 | 538296 | Bacteroidetes | Bacteroides | 89.3 | 0.00122 | 0.0076 |
| 28783 | 589071 | Bacteroidetes | Bacteroides | 94.1 | 0.00691 | 0.0010 |
| 30952 | 4390319 | Bacteroidetes | Bacteroides | 94.1 | 0.00307 | 0.0076 |
| 74244 | 363887 | Bacteroidetes | Bacteroides | 94.1 | 0.00831 | 0.0010 |
| 93359 | 681357 | Bacteroidetes | Bacteroides | 92.9 | 0.00704 | 0.0030 |
| 78673 | 219189 | Bacteroidetes | Chryseobacterium | 93.6 | 0.00570 | 0.0010 |
| 90245 | 736769 | Bacteroidetes | Chryseobacterium | 93.8 | 0.00263 | 0.0010 |
| 783 | 358766 | Bacteroidetes | Flavobacterium | 94.1 | 0.02181 | 0.0010 |
| 115019 | 961678 | Bacteroidetes | Flavobacterium | 92.9 | 0.00428 | 0.0410 |
| 5564 | 4296699 | Bacteroidetes | Gillisia | 94.1 | 0.00295 | 0.0010 |
| 60429 | 593226 | Bacteroidetes | Hymenobacter | 92.4 | 0.00158 | 0.0046 |
| 86424 | 1057116 | Bacteroidetes | Hymenobacter | 93.6 | 0.00180 | 0.0370 |
| 122203 | 145419 | Bacteroidetes | Krokinobacter | 93.8 | 0.00316 | 0.0027 |
| 101320 | 4326002 | Bacteroidetes | Lutibacter | 93.8 | 0.06359 | 0.0070 |
| 39188 | 80944 | Bacteroidetes | Maribacter | 93.8 | 0.00550 | 0.0360 |
| 43731 | 160714 | Bacteroidetes | Maribacter | 93.1 | 0.00174 | 0.0370 |
| 75478 | 149844 | Bacteroidetes | Marinifilum | 93.4 | 0.00265 | 0.0131 |
| 44209 | 4457195 | Bacteroidetes | Mucilaginibacter | 92.9 | 0.00209 | 0.0010 |
| 59832 | 248219 | Bacteroidetes | Myroides | 93.6 | 0.00497 | 0.0050 |
| 101194 | 1019904 | Bacteroidetes | Myroides | 93.1 | 0.00343 | 0.0236 |
| 7686 | 625911 | Bacteroidetes | Other Bacteroidales | 92.2 | 0.01065 | 0.0130 |
| 45963 | 4029448 | Bacteroidetes | Other Cryomorphaceae | 94.1 | 0.00469 | 0.0020 |
| 1851 | 170720 | Bacteroidetes | Other Flavobacteriaceae | 91.7 | 0.00407 | 0.0370 |
| 11821 | 4453765 | Bacteroidetes | Other Flavobacteriaceae | 93.8 | 0.00165 | 0.0218 |
| 37184 | 262661 | Bacteroidetes | Other Flavobacteriaceae | 93.8 | 0.01204 | 0.0010 |
| 47056 | 4346116 | Bacteroidetes | Other Flavobacteriaceae | 90.5 | 0.01400 | 0.0020 |
| 49967 | 519367 | Bacteroidetes | Other Flavobacteriaceae | 93.4 | 0.00281 | 0.0131 |
| 55141 | 3494920 | Bacteroidetes | Other Flavobacteriaceae | 91.3 | 0.00122 | 0.0330 |
| 85328 | 822144 | Bacteroidetes | Other Flavobacteriaceae | 93.6 | 0.00978 | 0.0010 |
| 101462 | 909231 | Bacteroidetes | Other Flavobacteriaceae | 93.8 | 0.00359 | 0.0020 |
| 101517 | 795842 | Bacteroidetes | Other Flavobacteriaceae | 94.1 | 0.01016 | 0.0010 |
| 111483 | 560027 | Bacteroidetes | Other Flavobacteriaceae | 92.7 | 0.00506 | 0.0370 |
| 112302 | 593919 | Bacteroidetes | Other Flavobacteriaceae | 92.4 | 0.01802 | 0.0010 |
| 131596 | 1013356 | Bacteroidetes | Other Flavobacteriaceae | 94.1 | 0.00196 | 0.0046 |
| 38742 | 537134 | Bacteroidetes | Other Flavobacteriales | 92.9 | 0.00780 | 0.0010 |
| 69992 | 4346003 | Bacteroidetes | Other Porphyromonadaceae | 94.1 | 0.01004 | 0.0010 |
| 105289 | 1112814 | Bacteroidetes | Other Porphyromonadaceae | 88.1 | 0.02255 | 0.0010 |
| 47882 | 2992307 | Bacteroidetes | Other Prevotellaceae | 93.8 | 0.00792 | 0.0190 |
| 42999 | 277106 | Bacteroidetes | Other Sphingobacteriaceae | 91.5 | 0.01014 | 0.0010 |
| 80042 | 277106 | Bacteroidetes | Other Sphingobacteriaceae | 91.5 | 0.00517 | 0.0046 |
| 14699 | 4453839 | Bacteroidetes | Polaribacter | 93.8 | 0.01121 | 0.0010 |
| 2965 | 1010283 | Bacteroidetes | Prevotella | 94.1 | 0.00215 | 0.0027 |
| 36163 | 577260 | Bacteroidetes | Prevotella | 93.4 | 0.01746 | 0.0010 |
| 66824 | 226618 | Bacteroidetes | Prevotella | 94.1 | 0.00219 | 0.0048 |
| 99884 | 694713 | Bacteroidetes | Prevotella | 93.6 | 0.00554 | 0.0046 |
| 19359 | 296609 | Bacteroidetes | Sphingobacterium | 91.7 | 0.00162 | 0.0218 |
| 53980 | 824606 | Bacteroidetes | Sphingobacterium | 94.1 | 0.00598 | 0.0010 |
| 97786 | 580835 | Bacteroidetes | Tenacibaculum | 94.1 | 0.04947 | 0.0010 |
| 120962 | 547181 | Bacteroidetes | Wautersiella | 93.8 | 0.00540 | 0.0070 |
|  |  |  |  |  |  |  |
| 112100 | 4362236 | Firmicutes | Acholeplasma | 94.3 | 0.09479 | 0.0010 |
| 131880 | 4380539 | Firmicutes | Bacillus | 92.7 | 0.00397 | 0.0040 |
| 21125 | 16103 | Firmicutes | Caldicellulosiruptor | 97.5 | 0.00292 | 0.0218 |
| 91915 | 4459733 | Firmicutes | Clostridium | 97.8 | 0.00216 | 0.0046 |
| 108001 | 4342297 | Firmicutes | Clostridium | 98.5 | 0.00490 | 0.0048 |
| 115751 | 54730 | Firmicutes | Holdemania | 89.8 | 0.00255 | 0.0027 |
| 6788 | 132829 | Firmicutes | Lactobacillus | 89.2 | 0.00192 | 0.0131 |
| 7251 | 4431608 | Firmicutes | Lactobacillus | 92.0 | 0.01572 | 0.0010 |
| 11653 | 4469032 | Firmicutes | Lactobacillus | 90.4 | 0.00388 | 0.0360 |
| 21207 | 4469032 | Firmicutes | Lactobacillus | 89.7 | 0.00127 | 0.0370 |
| 37337 | 4416659 | Firmicutes | Lactobacillus | 91.8 | 0.01087 | 0.0010 |
| 51570 | 4389033 | Firmicutes | Lactobacillus | 91.1 | 0.00866 | 0.0010 |
| 77689 | 4412873 | Firmicutes | Lactobacillus | 93.0 | 0.00759 | 0.0010 |
| 95073 | 4469032 | Firmicutes | Lactobacillus | 90.6 | 0.00535 | 0.0070 |
| 98265 | 4469032 | Firmicutes | Lactobacillus | 89.9 | 0.00268 | 0.0110 |
| 98446 | 84709 | Firmicutes | Lactobacillus | 93.0 | 0.00140 | 0.0027 |
| 108461 | 4469032 | Firmicutes | Lactobacillus | 90.9 | 0.00102 | 0.0010 |
| 119530 | 619224 | Firmicutes | Lactobacillus | 93.0 | 0.00726 | 0.0010 |
| 121194 | 221299 | Firmicutes | Lactobacillus | 90.6 | 0.00075 | 0.0370 |
| 126342 | 4389033 | Firmicutes | Lactobacillus | 91.8 | 0.00690 | 0.0010 |
| 52713 | 151623 | Firmicutes | Megasphaera | 92.5 | 0.00282 | 0.0076 |
| 89543 | 2978122 | Firmicutes | Oscillibacter | 98.3 | 0.00268 | 0.0236 |
| 35362 | 611528 | Firmicutes | Other Bacillaceae | 90.9 | 0.00569 | 0.0010 |
| 35484 | 1875545 | Firmicutes | Other Bacillaceae | 93.8 | 0.00339 | 0.0218 |
| 74307 | 4414596 | Firmicutes | Other Bacillaceae | 90.9 | 0.00955 | 0.0010 |
| 83145 | 1998479 | Firmicutes | Other Bacillales | 92.5 | 0.00573 | 0.0020 |
| 123432 | 245654 | Firmicutes | Other Bacillales | 92.3 | 0.00189 | 0.0218 |
| 20099 | 3793 | Firmicutes | Other Bacilli | 78.0 | 0.00324 | 0.0010 |
| 90492 | 715174 | Firmicutes | Other Clostridia | 97.0 | 0.00539 | 0.0048 |
| 95133 | 167215 | Firmicutes | Other Clostridiaceae | 98.3 | 0.01662 | 0.0020 |
| 66964 | 536989 | Firmicutes | Other Clostridiales | 98.5 | 0.00187 | 0.0076 |
| 99868 | 179861 | Firmicutes | Other Clostridiales | 98.0 | 0.00687 | 0.0131 |
| 383 | 200455 | Firmicutes | Other Firmicutes | 93.1 | 0.00263 | 0.0046 |
| 44254 | 240826 | Firmicutes | Other Firmicutes | 92.8 | 0.00573 | 0.0040 |
| 87768 | 315506 | Firmicutes | Other Firmicutes | 83.8 | 0.00062 | 0.0218 |
| 23403 | 2750808 | Firmicutes | Other Incertae_Sedis_XI | 98.5 | 0.00298 | 0.0350 |
| 100958 | 1108744 | Firmicutes | Other Incertae_Sedis_XI | 98.5 | 0.00518 | 0.0010 |
| 10470 | 259772 | Firmicutes | Other Lachnospiraceae | 97.0 | 0.00230 | 0.0048 |
| 32805 | 305437 | Firmicutes | Other Lachnospiraceae | 97.8 | 0.00114 | 0.0370 |
| 46514 | 540190 | Firmicutes | Other Lachnospiraceae | 98.8 | 0.00147 | 0.0370 |
| 327 | 15202 | Firmicutes | Other Lactobacillaceae | 85.8 | 0.00099 | 0.0370 |
| 31377 | 3225199 | Firmicutes | Other Lactobacillales | 81.4 | 0.00142 | 0.0027 |
| 32818 | 301921 | Firmicutes | Other Lactobacillales | 86.7 | 0.02869 | 0.0010 |
| 75887 | 147311 | Firmicutes | Other Lactobacillales | 88.0 | 0.00099 | 0.0027 |
| 83912 | 147311 | Firmicutes | Other Lactobacillales | 84.7 | 0.00068 | 0.0236 |
| 89494 | 288784 | Firmicutes | Other Lactobacillales | 85.4 | 0.00108 | 0.0010 |
| 111708 | 2682867 | Firmicutes | Other Lactobacillales | 83.3 | 0.00670 | 0.0150 |
| 29102 | 4387488 | Firmicutes | Other Ruminococcaceae | 94.6 | 0.00179 | 0.0131 |
| 54193 | 148501 | Firmicutes | Other Veillonellaceae | 87.8 | 0.00322 | 0.0370 |
| 77368 | 362899 | Firmicutes | Other Veillonellaceae | 90.7 | 0.00164 | 0.0236 |
| 12898 | 548679 | Firmicutes | Paenibacillus | 93.0 | 0.00870 | 0.0160 |
| 92440 | 4416610 | Firmicutes | Paenibacillus | 92.3 | 0.00211 | 0.0046 |
| 56888 | 2311550 | Firmicutes | Parasporobacterium | 97.3 | 0.00173 | 0.0236 |
| 106030 | 141835 | Firmicutes | Proteocatella | 98.3 | 0.00248 | 0.0140 |
| 130378 | 1107359 | Firmicutes | Tepidimicrobium | 98.8 | 0.00166 | 0.0076 |
| 61807 | 257863 | Firmicutes | Thermoanaerobacterium | 98.0 | 0.03772 | 0.0010 |
|  |  |  |  |  |  |  |
| 63307 | 345757 | Planctomycetes | Other Planctomycetaceae | 97.8 | 0.00112 | 0.0218 |
|  |  |  |  |  |  |  |
| 39050 | 4333237 | Proteobacteria | Acetobacter | 98.0 | 0.00237 | 0.0046 |
| 112268 | 580578 | Proteobacteria | Acidovorax | 91.6 | 0.00091 | 0.0218 |
| 19524 | 219826 | Proteobacteria | Acinetobacter | 90.2 | 0.00218 | 0.0370 |
| 43082 | 248521 | Proteobacteria | Acinetobacter | 92.5 | 0.01493 | 0.0010 |
| 116340 | 3217705 | Proteobacteria | Alkanindiges | 91.8 | 0.00770 | 0.0218 |
| 60783 | 529786 | Proteobacteria | Aquicella | 87.6 | 0.00194 | 0.0218 |
| 131914 | 555423 | Proteobacteria | Arcobacter | 98.5 | 0.00684 | 0.0010 |
| 116894 | 640589 | Proteobacteria | Brachymonas | 85.7 | 0.13450 | 0.0010 |
| 9899 | 132704 | Proteobacteria | Burkholderia | 92.7 | 0.00149 | 0.0370 |
| 101498 | 1131479 | Proteobacteria | Colwellia | 92.7 | 0.00124 | 0.0048 |
| 124536 | 230607 | Proteobacteria | Comamonas | 91.6 | 0.00156 | 0.0046 |
| 70762 | 150959 | Proteobacteria | Herminiimonas | 92.5 | 0.00234 | 0.0076 |
| 20609 | 633838 | Proteobacteria | Hydrogenophaga | 91.1 | 0.00199 | 0.0076 |
| 30728 | 280661 | Proteobacteria | Idiomarina | 92.7 | 0.00366 | 0.0076 |
| 60254 | 225370 | Proteobacteria | Marinomonas | 92.7 | 0.02114 | 0.0010 |
| 113502 | 225370 | Proteobacteria | Marinomonas | 92.5 | 0.00543 | 0.0310 |
| 22119 | 2307130 | Proteobacteria | Massilia | 92.7 | 0.00616 | 0.0060 |
| 70998 | 582997 | Proteobacteria | Massilia | 91.6 | 0.00324 | 0.0131 |
| 78150 | 85614 | Proteobacteria | Morganella | 92.7 | 0.00658 | 0.0010 |
| 7158 | 3796804 | Proteobacteria | Oligella | 92.5 | 0.00266 | 0.0048 |
| 61532 | 70363 | Proteobacteria | Oligella | 92.5 | 0.00716 | 0.0010 |
| 23123 | 830414 | Proteobacteria | Other Alphaproteobacteria | 98.5 | 0.00107 | 0.0236 |
| 49463 | 127587 | Proteobacteria | Other Alphaproteobacteria | 98.8 | 0.00196 | 0.0131 |
| 56179 | 4439836 | Proteobacteria | Other Alphaproteobacteria | 97.3 | 0.00665 | 0.0236 |
| 75189 | 807799 | Proteobacteria | Other Alphaproteobacteria | 95.0 | 0.00298 | 0.0236 |
| 70059 | 812024 | Proteobacteria | Other Alteromonadaceae | 92.5 | 0.01634 | 0.0010 |
| 54711 | 545268 | Proteobacteria | Other Alteromonadales | 92.3 | 0.00194 | 0.0236 |
| 70270 | 1141071 | Proteobacteria | Other Brucellaceae | 97.8 | 0.00226 | 0.0220 |
| 47099 | 215097 | Proteobacteria | Other Burkholderiales | 92.5 | 0.00909 | 0.0010 |
| 62967 | 830764 | Proteobacteria | Other Comamonadaceae | 91.6 | 0.00158 | 0.0236 |
| 38679 | 783638 | Proteobacteria | Other Enterobacteriaceae | 91.6 | 0.00774 | 0.0010 |
| 50516 | 274365 | Proteobacteria | Other Enterobacteriaceae | 92.5 | 0.00773 | 0.0010 |
| 58588 | 219248 | Proteobacteria | Other Enterobacteriaceae | 91.6 | 0.02206 | 0.0010 |
| 104340 | 4358093 | Proteobacteria | Other Enterobacteriaceae | 84.5 | 0.00194 | 0.0046 |
| 80724 | 810399 | Proteobacteria | Other Enterococcaceae | 93.0 | 0.00624 | 0.0010 |
| 12248 | 96014 | Proteobacteria | Other Gammaproteobacteria | 93.0 | 0.00670 | 0.0010 |
| 28553 | 795623 | Proteobacteria | Other Gammaproteobacteria | 92.7 | 0.00816 | 0.0010 |
| 57889 | 756297 | Proteobacteria | Other Gammaproteobacteria | 93.0 | 0.00364 | 0.0030 |
| 62659 | 831634 | Proteobacteria | Other Gammaproteobacteria | 93.0 | 0.00753 | 0.0010 |
| 64820 | 589597 | Proteobacteria | Other Gammaproteobacteria | 85.1 | 0.00079 | 0.0076 |
| 82746 | 642309 | Proteobacteria | Other Gammaproteobacteria | 93.0 | 0.06463 | 0.0010 |
| 83281 | 1918737 | Proteobacteria | Other Gammaproteobacteria | 93.0 | 0.00926 | 0.0410 |
| 86617 | 593801 | Proteobacteria | Other Gammaproteobacteria | 93.0 | 0.04347 | 0.0010 |
| 98321 | 581393 | Proteobacteria | Other Gammaproteobacteria | 93.0 | 0.00357 | 0.0040 |
| 125729 | 4373778 | Proteobacteria | Other Gammaproteobacteria | 93.0 | 0.00796 | 0.0010 |
| 128295 | 3310570 | Proteobacteria | Other Gammaproteobacteria | 93.0 | 0.00752 | 0.0010 |
| 79178 | 255657 | Proteobacteria | Other Halomonadaceae | 92.5 | 0.00465 | 0.0236 |
| 97135 | 312197 | Proteobacteria | Other Methylophilaceae | 91.8 | 0.00517 | 0.0060 |
| 79624 | 4303853 | Proteobacteria | Other Neisseriaceae | 90.4 | 0.00346 | 0.0040 |
| 21356 | 677064 | Proteobacteria | Other Oxalobacteraceae | 83.4 | 0.00527 | 0.0070 |
| 40342 | 304491 | Proteobacteria | Other Oxalobacteraceae | 84.3 | 0.00125 | 0.0010 |
| 45872 | 1126662 | Proteobacteria | Other Oxalobacteraceae | 91.8 | 0.00094 | 0.0046 |
| 53645 | 4445096 | Proteobacteria | Other Oxalobacteraceae | 82.9 | 0.00199 | 0.0048 |
| 72344 | 661838 | Proteobacteria | Other Oxalobacteraceae | 80.3 | 0.00153 | 0.0218 |
| 133671 | 1137185 | Proteobacteria | Other Oxalobacteraceae | 89.2 | 0.00227 | 0.0048 |
| 29075 | 256955 | Proteobacteria | Other Proteobacteria | 98.3 | 0.00339 | 0.0010 |
| 34595 | 769643 | Proteobacteria | Other Proteobacteria | 88.8 | 0.00062 | 0.0218 |
| 67963 | 589597 | Proteobacteria | Other Pseudomonadaceae | 90.4 | 0.00102 | 0.0010 |
| 98831 | 568727 | Proteobacteria | Other Pseudomonadaceae | 92.5 | 0.00532 | 0.0020 |
| 113275 | 273239 | Proteobacteria | Other Pseudomonadaceae | 92.5 | 0.00375 | 0.0150 |
| 91215 | 417366 | Proteobacteria | Other Rhizobiales | 98.0 | 0.00408 | 0.0010 |
| 125265 | 4300153 | Proteobacteria | Other Rhodobacteraceae | 86.7 | 0.00084 | 0.0236 |
| 16531 | 1138056 | Proteobacteria | Paracoccus | 95.5 | 0.00216 | 0.0048 |
| 74507 | 812921 | Proteobacteria | Paracoccus | 94.3 | 0.00498 | 0.0070 |
| 10773 | 1011514 | Proteobacteria | Pelagibacter | 98.8 | 0.01073 | 0.0010 |
| 93763 | 326484 | Proteobacteria | Pelagibacter | 98.8 | 0.02103 | 0.0010 |
| 117730 | 4317875 | Proteobacteria | Petrobacter | 89.7 | 0.00126 | 0.0236 |
| 34686 | 161024 | Proteobacteria | Photobacterium | 91.6 | 0.00288 | 0.0046 |
| 10274 | 4465803 | Proteobacteria | Porphyromonas | 93.4 | 0.00747 | 0.0236 |
| 27125 | 1129328 | Proteobacteria | Providencia | 93.0 | 0.00659 | 0.0030 |
| 4171 | 295031 | Proteobacteria | Pseudomonas | 91.6 | 0.00722 | 0.0010 |
| 18375 | 541223 | Proteobacteria | Pseudomonas | 91.1 | 0.00113 | 0.0076 |
| 73379 | 256215 | Proteobacteria | Pseudomonas | 92.5 | 0.00620 | 0.0340 |
| 108121 | 338200 | Proteobacteria | Pseudomonas | 91.3 | 0.00247 | 0.0010 |
| 124253 | 295031 | Proteobacteria | Pseudomonas | 91.3 | 0.00281 | 0.0030 |
| 125558 | 103728 | Proteobacteria | Pseudomonas | 91.3 | 0.00100 | 0.0048 |
| 125912 | 312742 | Proteobacteria | Pseudomonas | 92.5 | 0.00238 | 0.0218 |
| 108061 | 766791 | Proteobacteria | Pseudoxanthomonas | 91.1 | 0.01557 | 0.0010 |
| 38959 | 55243 | Proteobacteria | Roseomonas | 97.8 | 0.00208 | 0.0370 |
| 18238 | 643857 | Proteobacteria | Salinisphaera | 91.1 | 0.00131 | 0.0046 |
| 28375 | 759061 | Proteobacteria | Serratia | 93.0 | 0.00551 | 0.0020 |
| 3337 | 286679 | Proteobacteria | Shewanella | 90.6 | 0.00119 | 0.0370 |
| 3751 | 286679 | Proteobacteria | Shewanella | 90.6 | 0.00233 | 0.0048 |
| 11476 | 286679 | Proteobacteria | Shewanella | 90.6 | 0.00129 | 0.0218 |
| 12690 | 286679 | Proteobacteria | Shewanella | 90.9 | 0.00297 | 0.0010 |
| 22272 | 286679 | Proteobacteria | Shewanella | 91.3 | 0.00310 | 0.0010 |
| 22890 | 286679 | Proteobacteria | Shewanella | 90.9 | 0.00247 | 0.0048 |
| 58733 | 189118 | Proteobacteria | Shewanella | 92.3 | 0.00242 | 0.0131 |
| 63068 | 286679 | Proteobacteria | Shewanella | 90.4 | 0.00149 | 0.0370 |
| 64604 | 286679 | Proteobacteria | Shewanella | 90.9 | 0.00269 | 0.0010 |
| 79923 | 3974550 | Proteobacteria | Shewanella | 92.7 | 0.01066 | 0.0010 |
| 86598 | 286679 | Proteobacteria | Shewanella | 91.1 | 0.00125 | 0.0370 |
| 101282 | 286679 | Proteobacteria | Shewanella | 90.6 | 0.00297 | 0.0027 |
| 105421 | 663258 | Proteobacteria | Shewanella | 90.8 | 0.00253 | 0.0046 |
| 94557 | 276010 | Proteobacteria | Stenotrophomonas | 92.5 | 0.00566 | 0.0020 |
| 37339 | 46595 | Proteobacteria | Sulfitobacter | 98.8 | 0.00351 | 0.0210 |
| 3242 | 343783 | Proteobacteria | Thalassobacter | 98.8 | 0.02168 | 0.0010 |
| 87297 | 4393354 | Proteobacteria | Vibrio | 93.0 | 0.00651 | 0.0080 |
|  |  |  |  |  |  |  |
| 2464 | 174919 | Thermotogae | Fervidobacterium | 98.3 | 0.00214 | 0.0236 |

^1^ unique number assigned to sequences with less than 97% similarity to other sequences

^2^ number that is associated with the sequence in the aligned Greengenes database that fits the query sequence

^3^ percent identity of the query sequence to the assigned Greengenes reference sequence

**Table S7: Full list of Operational Taxonomic Units (OTUs) found at significantly different relative abundances in the distal intestine of fresh- and seawater Atlantic salmon, as identified by Metastats analysis.**

| **denovo ID^1^** | **Greengenes ID^2^** | **Phylum** | **Taxonomic assignment** | **Blast %^3^** | **mean % FW** | **mean % SW** | **pvalue** |
| --- | --- | --- | --- | --- | --- | --- | --- |
| 9553 | 4421933 | Acidobacteria | Other Gp4 | 97.3 | 0.0681 | 0.0002 | 0.036 |
| 68028 | 636687 | Acidobacteria | Other Gp4 | 97.5 | 0.9923 | 0.0001 | 0.014 |
| 91061 | 209520 | Acidobacteria | Other Gp4 | 98.0 | 0.0013 | 0.0001 | < 0.001 |
|  |  |  |  |  |  |  |  |
| 40108 | 816529 | Actinobacteria | Arthrobacter | 95.0 | 0.0021 | 0.0204 | 0.001 |
| 112184 | 362790 | Actinobacteria | Arthrobacter | 97.5 | 0.1273 | 0.0243 | 0.029 |
| 124434 | 4354149 | Actinobacteria | Arthrobacter | 94.0 | 0.0080 | 0.0001 | < 0.001 |
| 109986 | 788268 | Actinobacteria | Cellulomonas | 97.3 | 0.0024 | 0.0002 | 0.035 |
| 133163 | 799959 | Actinobacteria | Conexibacter | 92.3 | 0.0041 | 0.0002 | < 0.001 |
| 8542 | 1146291 | Actinobacteria | Corynebacterium | 96.6 | 0.1127 | 0.0145 | 0.030 |
| 68230 | 974249 | Actinobacteria | Corynebacterium | 96.3 | 0.0089 | 0.0001 | 0.031 |
| 70395 | 503389 | Actinobacteria | Corynebacterium | 96.6 | 0.0851 | 0.0069 | 0.019 |
| 85802 | 282360 | Actinobacteria | Corynebacterium | 96.1 | 0.0050 | 0.0049 | 0.010 |
| 113750 | 446403 | Actinobacteria | Corynebacterium | 96.1 | 0.1359 | 0.0348 | 0.043 |
| 127667 | 211156 | Actinobacteria | Corynebacterium | 95.8 | 0.0016 | 0.0003 | 0.036 |
| 99573 | 4458619 | Actinobacteria | Dermacoccus | 97.3 | 0.0943 | 0.0053 | 0.030 |
| 71442 | 317451 | Actinobacteria | Friedmanniella | 97.5 | 0.0080 | 0.0001 | 0.029 |
| 85345 | 4432889 | Actinobacteria | Janibacter | 97.3 | 0.1342 | 0.0087 | 0.012 |
| 86291 | 368097 | Actinobacteria | Kocuria | 97.3 | 0.0726 | 0.0139 | 0.031 |
| 82846 | 821644 | Actinobacteria | Leucobacter | 96.3 | 0.0106 | 0.0012 | < 0.001 |
| 39852 | 103628 | Actinobacteria | Microbacterium | 97.1 | 0.0264 | 0.0022 | 0.039 |
| 83343 | 252119 | Actinobacteria | Micrococcus | 97.5 | 0.2017 | 0.0249 | 0.005 |
| 30118 | 28341 | Actinobacteria | Mycobacterium | 94.3 | 0.0047 | 0.0013 | 0.029 |
| 30043 | 151442 | Actinobacteria | Nocardioides | 96.8 | 0.0266 | 0.0004 | 0.029 |
| 96147 | 971270 | Actinobacteria | Nocardioides | 93.3 | 0.0435 | 0.0006 | 0.005 |
| 72801 | 384657 | Actinobacteria | Other Actinomycetales | 96.6 | 0.0047 | 0.0006 | 0.001 |
| 70482 | 232900 | Actinobacteria | Other Coriobacteriaceae | 92.8 | 0.2007 | 0.0006 | 0.001 |
| 101804 | 247757 | Actinobacteria | Other Coriobacteriaceae | 90.0 | 0.1054 | 0.0146 | 0.025 |
| 15704 | 842941 | Actinobacteria | Other Microbacteriaceae | 97.5 | 0.1532 | 0.0600 | 0.015 |
| 57967 | 4331137 | Actinobacteria | Other Microbacteriaceae | 95.6 | 0.0018 | 0.0010 | 0.013 |
| 63899 | 256173 | Actinobacteria | Other Micrococcaceae | 93.8 | 0.0030 | 0.0014 | 0.044 |
| 75768 | 12727 | Actinobacteria | Propionibacterium | 96.3 | 0.0004 | 0.0051 | 0.006 |
| 134940 | 933896 | Actinobacteria | Propionibacterium | 97.5 | 0.2023 | 0.0195 | 0.001 |
| 92451 | 4339777 | Actinobacteria | Rothia | 97.5 | 0.1986 | 0.0034 | 0.010 |
| 98317 | 4387303 | Actinobacteria | Streptomyces | 96.1 | 0.0055 | 0.0006 | < 0.001 |
| 34766 | 4422519 | Actinobacteria | Turicella | 94.3 | 0.0016 | 0.0017 | 0.002 |
|  |  |  |  |  |  |  |  |
| 102456 | 1123250 | Bacteroidetes | Bacteroides | 94.1 | 0.0024 | 0.0010 | 0.013 |
| 127415 | 258175 | Bacteroidetes | Chryseobacterium | 93.8 | 0.0828 | 0.0087 | 0.015 |
| 45712 | 4402671 | Bacteroidetes | Flavobacterium | 94.1 | 0.0130 | 0.0645 | 0.029 |
| 73454 | 251173 | Bacteroidetes | Flavobacterium | 93.8 | 0.0005 | 0.0299 | 0.001 |
| 117017 | 1138568 | Bacteroidetes | Flavobacterium | 92.9 | 0.0063 | 0.0003 | < 0.001 |
| 129456 | 256046 | Bacteroidetes | Flavobacterium | 92.9 | 0.0001 | 0.0042 | 0.007 |
| 26560 | 626864 | Bacteroidetes | Other Flavobacteriaceae | 93.8 | 0.0005 | 0.0129 | 0.010 |
| 32640 | 568419 | Bacteroidetes | Other Flavobacteriaceae | 93.8 | 0.0078 | 0.0006 | < 0.001 |
| 46045 | 277106 | Bacteroidetes | Other Sphingobacteriaceae | 93.1 | 0.0004 | 0.0615 | 0.001 |
| 19354 | 323139 | Bacteroidetes | Porphyromonadaceae | 93.8 | 0.0013 | 0.0072 | 0.034 |
| 27264 | 4338742 | Bacteroidetes | Prevotella | 93.8 | 0.0001 | 0.4466 | 0.001 |
| 28167 | 4304901 | Bacteroidetes | Prevotella | 93.8 | 0.0089 | 0.0018 | < 0.001 |
| 80182 | 4332410 | Bacteroidetes | Prevotella | 94.1 | 0.0039 | 0.0002 | < 0.001 |
| 90906 | 2096960 | Bacteroidetes | Weeksella | 91.7 | 0.0008 | 0.0054 | 0.021 |
|  |  |  |  |  |  |  |  |
| 50285 | 4335830 | Chlamydiae | Other Chlamydiales | 85.5 | 0.0039 | 0.0003 | 0.001 |
|  |  |  |  |  |  |  |  |
| 56639 | 4396446 | Deinococcus-Thermus | Deinococcus | 92.6 | 0.0024 | 0.0001 | 0.013 |
|  |  |  |  |  |  |  |  |
| 11638 | 408492 | Firmicutes | Anaerococcus | 98.2 | 0.0437 | 0.0004 | 0.007 |
| 30564 | 236650 | Firmicutes | Anaerococcus | 98.5 | 0.0099 | 0.0018 | 0.023 |
| 133132 | 4330001 | Firmicutes | Anaerovorax | 93.6 | 0.0056 | 0.0002 | 0.001 |
| 86287 | 4449704 | Firmicutes | Aneurinibacillus | 93.4 | 0.1036 | 0.0007 | 0.015 |
| 113344 | 4473250 | Firmicutes | Aneurinibacillus | 93.4 | 0.0063 | 0.0001 | < 0.001 |
| 11867 | 4361178 | Firmicutes | Bacillus | 91.4 | 0.0244 | 0.0006 | 0.010 |
| 34383 | 159299 | Firmicutes | Bacillus | 92.8 | 0.1334 | 0.0182 | 0.001 |
| 53322 | 14592 | Firmicutes | Bacillus | 91.4 | 0.0036 | 0.0010 | 0.036 |
| 64550 | 115246 | Firmicutes | Bacillus | 92.7 | 0.0026 | 0.0003 | 0.005 |
| 67761 | 554916 | Firmicutes | Bacillus | 91.8 | 0.0593 | 0.0009 | 0.001 |
| 87945 | 4455979 | Firmicutes | Bacillus | 92.7 | 0.0505 | 0.0001 | 0.047 |
| 121533 | 4364491 | Firmicutes | Bacillus | 92.8 | 0.2430 | 0.0214 | 0.001 |
| 99251 | 1016369 | Firmicutes | Brochothrix | 92.5 | 0.0284 | 0.2196 | 0.001 |
| 27574 | 183703 | Firmicutes | Butyricicoccus | 97.5 | 0.0056 | 0.0007 | 0.004 |
| 119265 | 686789 | Firmicutes | Carnobacterium | 93.0 | 0.0822 | 0.0189 | 0.011 |
| 47024 | 104766 | Firmicutes | Cerasibacillus | 92.8 | 0.1787 | 0.0152 | 0.002 |
| 9407 | 1105928 | Firmicutes | Clostridium | 98.8 | 0.4688 | 0.0002 | 0.001 |
| 18923 | 791998 | Firmicutes | Clostridium | 98.8 | 0.6298 | 0.0011 | 0.001 |
| 32916 | 16164 | Firmicutes | Clostridium | 93.3 | 0.0017 | 0.0002 | 0.036 |
| 59371 | 2234838 | Firmicutes | Clostridium | 98.5 | 0.1166 | 0.0005 | 0.001 |
| 63828 | 707087 | Firmicutes | Clostridium | 98.8 | 0.0382 | 0.0001 | 0.001 |
| 86334 | 322130 | Firmicutes | Clostridium | 98.8 | 0.1563 | 0.0030 | 0.001 |
| 97515 | 3576174 | Firmicutes | Clostridium | 97.8 | 0.3053 | 0.0066 | 0.001 |
| 102495 | 1140978 | Firmicutes | Clostridium | 98.8 | 0.4335 | 0.0072 | 0.001 |
| 108145 | 94261 | Firmicutes | Clostridium | 98.8 | 0.0878 | 0.0001 | 0.001 |
| 124540 | 616688 | Firmicutes | Clostridium | 97.8 | 0.1566 | 0.0048 | 0.001 |
| 14742 | 132661 | Firmicutes | Enterococcus | 92.8 | 0.0954 | 0.0044 | 0.002 |
| 83459 | 171416 | Firmicutes | Gemella | 85.7 | 0.0785 | 0.0123 | 0.004 |
| 15416 | 192776 | Firmicutes | Lactobacillus | 92.3 | 0.0005 | 0.0305 | 0.001 |
| 16346 | 4414257 | Firmicutes | Lactobacillus | 93.0 | 0.0526 | 1.9323 | 0.001 |
| 19383 | 578837 | Firmicutes | Lactobacillus | 92.5 | 0.0005 | 0.0055 | 0.030 |
| 21895 | 3136117 | Firmicutes | Lactobacillus | 91.6 | 0.0493 | 0.0037 | 0.001 |
| 24376 | 745465 | Firmicutes | Lactobacillus | 91.8 | 0.0019 | 0.0334 | 0.001 |
| 26683 | 187233 | Firmicutes | Lactobacillus | 93.0 | 4.5098 | 1.1421 | 0.002 |
| 31241 | 4411596 | Firmicutes | Lactobacillus | 92.5 | 1.0083 | 0.0128 | 0.001 |
| 31656 | 343431 | Firmicutes | Lactobacillus | 93.0 | 1.0623 | 0.1226 | 0.001 |
| 47949 | 4168094 | Firmicutes | Lactobacillus | 93.0 | 0.0035 | 0.0005 | 0.014 |
| 54361 | 433086 | Firmicutes | Lactobacillus | 93.0 | 0.0697 | 0.5885 | 0.001 |
| 79231 | 338757 | Firmicutes | Lactobacillus | 92.5 | 0.5920 | 0.0465 | 0.001 |
| 86684 | 281310 | Firmicutes | Lactobacillus | 93.0 | 0.0011 | 0.0224 | 0.028 |
| 87732 | 3136117 | Firmicutes | Lactobacillus | 91.3 | 0.0069 | 0.0001 | < 0.001 |
| 108414 | 543173 | Firmicutes | Lactobacillus | 93.0 | 0.1135 | 0.0040 | 0.011 |
| 128257 | 3136117 | Firmicutes | Lactobacillus | 91.1 | 0.0029 | 0.0002 | 0.002 |
| 131858 | 4469032 | Firmicutes | Lactobacillus | 93.0 | 0.5905 | 39.5699 | 0.001 |
| 67625 | 830659 | Firmicutes | Lactococcus | 92.7 | 0.0155 | 0.4643 | 0.001 |
| 79073 | 557570 | Firmicutes | Lactococcus | 92.7 | 0.5522 | 0.0600 | 0.001 |
| 85720 | 1130824 | Firmicutes | Lactococcus | 92.7 | 0.0370 | 0.0005 | 0.026 |
| 26692 | 254021 | Firmicutes | Leuconostoc | 92.5 | 0.3789 | 0.0395 | 0.001 |
| 43300 | 298813 | Firmicutes | Leuconostoc | 92.5 | 0.0021 | 0.0004 | 0.035 |
| 59078 | 540940 | Firmicutes | Leuconostoc | 91.3 | 0.0040 | 0.0024 | 0.019 |
| 80278 | 811179 | Firmicutes | Lysinibacillus | 91.3 | 0.0039 | 0.0002 | 0.005 |
| 87568 | 1125443 | Firmicutes | Lysinibacillus | 93.0 | 0.0443 | 0.0089 | 0.036 |
| 99617 | 4319637 | Firmicutes | Lysinibacillus | 93.0 | 0.0780 | 0.0108 | 0.011 |
| 82484 | 1027904 | Firmicutes | Macrococcus | 92.5 | 0.0402 | 0.0013 | 0.032 |
| 13708 | 3039313 | Firmicutes | Megasphaera | 92.7 | 0.1070 | 0.0006 | 0.001 |
| 54132 | 1132622 | Firmicutes | Megasphaera | 92.5 | 0.0058 | 0.1959 | 0.001 |
| 99941 | 1145719 | Firmicutes | Megasphaera | 92.3 | 0.0031 | 0.0002 | < 0.001 |
| 73550 | 1126217 | Firmicutes | Oceanobacillus | 92.7 | 0.2291 | 0.0021 | 0.001 |
| 41683 | 1115106 | Firmicutes | Other Bacillaceae | 92.8 | 0.0037 | 0.0567 | 0.001 |
| 82907 | 152111 | Firmicutes | Other Bacillaceae | 93.0 | 0.0058 | 0.0208 | 0.031 |
| 109921 | 244056 | Firmicutes | Other Bacillaceae | 92.5 | 0.0400 | 0.0001 | 0.002 |
| 127807 | 4481478 | Firmicutes | Other Bacillaceae | 92.3 | 0.0048 | 0.0008 | < 0.001 |
| 18954 | 366937 | Firmicutes | Other Bacillales | 90.9 | 0.0306 | 0.0001 | 0.003 |
| 47182 | 708285 | Firmicutes | Other Clostridiales | 94.6 | 0.0186 | 0.0033 | 0.023 |
| 48619 | 308309 | Firmicutes | Other Clostridiales | 98.8 | 0.0646 | 0.0073 | 0.023 |
| 67893 | 300267 | Firmicutes | Other Clostridiales | 95.3 | 0.1896 | 0.0052 | 0.001 |
| 86614 | 755148 | Firmicutes | Other Clostridiales | 98.5 | 0.0071 | 0.0006 | < 0.001 |
| 9134 | 146014 | Firmicutes | Other Incertae_Sedis_XI | 98.8 | 0.1372 | 0.0013 | 0.001 |
| 52243 | 768514 | Firmicutes | Other Incertae_Sedis_XI | 98.3 | 0.1143 | 0.0028 | 0.001 |
| 57843 | 2597238 | Firmicutes | Other Incertae_Sedis_XI | 95.5 | 0.0029 | 0.0005 | 0.035 |
| 10368 | 297648 | Firmicutes | Other Lachnospiraceae | 98.8 | 0.0075 | 0.0003 | < 0.001 |
| 32730 | 104740 | Firmicutes | Other Lachnospiraceae | 93.3 | 0.0792 | 0.0110 | 0.013 |
| 48071 | 4358921 | Firmicutes | Other Lachnospiraceae | 98.8 | 0.0034 | 0.0035 | 0.010 |
| 130933 | 347553 | Firmicutes | Other Peptostreptococcaceae | 98.5 | 0.0071 | 0.0015 | 0.009 |
| 39574 | 694337 | Firmicutes | Other Ruminococcaceae | 92.8 | 1.9023 | 0.1046 | 0.001 |
| 104533 | 743075 | Firmicutes | Other Ruminococcaceae | 97.0 | 0.0041 | 0.0004 | < 0.001 |
| 80339 | 102222 | Firmicutes | Pediococcus | 93.0 | 0.2850 | 0.0038 | 0.001 |
| 21990 | 252727 | Firmicutes | Peptostreptococcus | 98.8 | 0.6236 | 0.0735 | 0.001 |
| 102705 | 342268 | Firmicutes | Peptostreptococcus | 95.8 | 0.2265 | 0.0245 | 0.001 |
| 83746 | 198071 | Firmicutes | Roseburia | 98.8 | 0.0041 | 0.0008 | 0.029 |
| 62210 | 4308213 | Firmicutes | Selenomonas | 92.7 | 0.0082 | 0.0001 | < 0.001 |
| 106504 | 368134 | Firmicutes | Staphylococcus | 93.0 | 1.1299 | 0.0771 | 0.001 |
| 111908 | 1040220 | Firmicutes | Staphylococcus | 91.6 | 0.0054 | 0.0001 | < 0.001 |
| 10222 | 4309550 | Firmicutes | Streptococcus | 92.3 | 0.1328 | 0.0190 | 0.001 |
| 14478 | 4337090 | Firmicutes | Streptococcus | 92.7 | 2.6784 | 0.0931 | 0.001 |
| 58824 | 299659 | Firmicutes | Streptococcus | 92.7 | 9.4564 | 0.1409 | 0.001 |
| 3117 | 244503 | Firmicutes | Tepidimicrobium | 97.3 | 0.0621 | 0.0078 | 0.038 |
| 92138 | 921110 | Firmicutes | Vagococcus | 92.0 | 0.1695 | 0.0065 | 0.001 |
| 20354 | 962249 | Firmicutes | Veillonella | 92.7 | 0.1619 | 0.0088 | 0.012 |
| 83740 | 222433 | Firmicutes | Veillonella | 92.7 | 0.8718 | 0.0023 | 0.001 |
| 114465 | 1133367 | Firmicutes | Virgibacillus | 92.8 | 0.0052 | 0.0006 | < 0.001 |
| 7597 | 64384 | Firmicutes | Weissella | 91.1 | 0.0144 | 0.0020 | 0.003 |
| 9741 | 299879 | Firmicutes | Weissella | 91.1 | 0.0277 | 0.0019 | 0.001 |
| 17221 | 299879 | Firmicutes | Weissella | 91.3 | 0.0235 | 0.0031 | 0.001 |
| 37091 | 299879 | Firmicutes | Weissella | 90.9 | 0.0079 | 0.0002 | < 0.001 |
| 53684 | 299879 | Firmicutes | Weissella | 91.1 | 0.0072 | 0.0012 | 0.001 |
| 57199 | 299879 | Firmicutes | Weissella | 91.1 | 0.0527 | 0.0072 | 0.001 |
| 66183 | 64384 | Firmicutes | Weissella | 91.1 | 0.0055 | 0.0002 | < 0.001 |
| 68545 | 64384 | Firmicutes | Weissella | 90.6 | 0.0037 | 0.0006 | 0.005 |
| 70485 | 299879 | Firmicutes | Weissella | 91.1 | 0.0034 | 0.0001 | 0.036 |
| 71383 | 64384 | Firmicutes | Weissella | 90.9 | 0.0043 | 0.0014 | 0.002 |
| 78259 | 64384 | Firmicutes | Weissella | 90.9 | 0.0335 | 0.0015 | 0.002 |
| 82072 | 299879 | Firmicutes | Weissella | 90.9 | 0.0030 | 0.0004 | 0.002 |
| 133624 | 64384 | Firmicutes | Weissella | 90.6 | 0.0024 | 0.0001 | 0.036 |
| 134510 | 64384 | Firmicutes | Weissella | 90.9 | 0.0091 | 0.0004 | 0.003 |
|  |  |  |  |  |  |  |  |
| 39062 | 4435370 | Fusobacteria | Cetobacterium | 97.3 | 0.0521 | 0.0057 | 0.006 |
| 123538 | 11302 | Fusobacteria | Cetobacterium | 97.5 | 0.0257 | 0.0032 | 0.028 |
| 57300 | 2614328 | Fusobacteria | Fusobacterium | 97.8 | 0.1542 | 0.0030 | 0.002 |
| 57554 | 1654477 | Fusobacteria | Fusobacterium | 95.8 | 0.2926 | 0.0165 | 0.001 |
| 244 | 345114 | Fusobacteria | Other Fusobacteriaceae | 94.8 | 0.3524 | 0.0877 | 0.002 |
| 19435 | 4439398 | Fusobacteria | Other Fusobacteriaceae | 93.8 | 0.0034 | 0.0002 | 0.001 |
| 97945 | 572889 | Fusobacteria | Other Fusobacteriaceae | 95.6 | 0.4785 | 0.0649 | 0.001 |
| 70239 | 726699 | Fusobacteria | Psychrilyobacter | 97.0 | 0.2300 | 0.0381 | 0.001 |
|  |  |  |  |  |  |  |  |
| 60355 | 4477462 | OD1 | Other OD1_genera_incertae_sedis | 86.8 | 0.0013 | 0.0003 | 0.036 |
| 86736 | 678059 | OD1 | Other OD1_genera_incertae_sedis | 85.0 | 0.0019 | 0.0003 | 0.036 |
| 115167 | 4467411 | OD1 | Other OD1_genera_incertae_sedis | 95.3 | 0.0167 | 0.0001 | 0.023 |
|  |  |  |  |  |  |  |  |
| 112590 | 889025 | Proteobacteria | Acinetobacter | 92.1 | 0.0546 | 0.0001 | 0.007 |
| 129922 | 203157 | Proteobacteria | Aeromonas | 92.3 | 0.0030 | 0.0001 | 0.002 |
| 19284 | 225259 | Proteobacteria | Aquabacterium | 93.0 | 0.0253 | 0.0001 | 0.001 |
| 24003 | 4455962 | Proteobacteria | Arcobacter | 97.8 | 0.0019 | 0.3497 | 0.001 |
| 39576 | 2655357 | Proteobacteria | Azohydromonas | 92.3 | 0.0047 | 0.0011 | 0.002 |
| 97226 | 4353264 | Proteobacteria | Brevundimonas | 98.8 | 0.2438 | 0.0333 | 0.002 |
| 85555 | 256276 | Proteobacteria | Brucella | 98.8 | 0.2916 | 0.0250 | 0.001 |
| 131794 | 151835 | Proteobacteria | Collimonas | 83.4 | 0.0029 | 0.0007 | 0.031 |
| 68943 | 807416 | Proteobacteria | Colwellia | 92.7 | 0.0060 | 0.0003 | < 0.001 |
| 124977 | 749805 | Proteobacteria | Cupriavidus | 84.3 | 0.0099 | 0.0004 | < 0.001 |
| 37352 | 834138 | Proteobacteria | Delftia | 83.6 | 0.0114 | 0.0788 | 0.001 |
| 57471 | 574102 | Proteobacteria | Enhydrobacter | 92.8 | 0.0211 | 0.0010 | 0.030 |
| 81007 | 91557 | Proteobacteria | Erwinia | 91.1 | 0.0020 | 0.0006 | 0.036 |
| 22350 | 114510 | Proteobacteria | Escherichia/Shigella | 93.0 | 20.7405 | 0.6304 | 0.001 |
| 107880 | 558981 | Proteobacteria | Lysobacter | 92.7 | 0.2826 | 0.0002 | 0.015 |
| 4789 | 360547 | Proteobacteria | Mesorhizobium | 97.5 | 0.1340 | 0.0302 | 0.001 |
| 40919 | 4303249 | Proteobacteria | Methylobacterium | 98.0 | 0.0148 | 0.0017 | 0.047 |
| 82604 | 979344 | Proteobacteria | Methylobacterium | 98.8 | 0.1513 | 0.0005 | 0.026 |
| 84435 | 677165 | Proteobacteria | Methylobacterium | 98.8 | 0.1217 | 0.0046 | 0.002 |
| 115111 | 360826 | Proteobacteria | Other Acetobacteraceae | 96.5 | 0.0008 | 0.0003 | 0.036 |
| 59109 | 661229 | Proteobacteria | Other Alcaligenaceae | 92.0 | 0.0056 | 0.0001 | < 0.001 |
| 102205 | 94511 | Proteobacteria | Other Alphaproteobacteria | 95.5 | 0.0106 | 0.0007 | 0.043 |
| 75940 | 7425 | Proteobacteria | Other Betaproteobacteria | 89.0 | 0.2371 | 0.0002 | 0.013 |
| 4869 | 340208 | Proteobacteria | Other Comamonadaceae | 92.0 | 0.0236 | 0.0005 | 0.042 |
| 48927 | 230607 | Proteobacteria | Other Comamonadaceae | 84.1 | 0.0012 | 0.0067 | 0.032 |
| 21918 | 4466618 | Proteobacteria | Other Enterobacteriaceae | 91.1 | 0.0062 | 0.0002 | 0.008 |
| 27265 | 1101413 | Proteobacteria | Other Enterobacteriaceae | 93.0 | 0.0047 | 1.3902 | 0.001 |
| 27547 | 569459 | Proteobacteria | Other Enterobacteriaceae | 93.0 | 0.0361 | 0.4983 | 0.001 |
| 69873 | 4466618 | Proteobacteria | Other Enterobacteriaceae | 91.1 | 0.0029 | 0.0003 | 0.014 |
| 79986 | 583590 | Proteobacteria | Other Enterobacteriaceae | 93.0 | 0.0014 | 0.0880 | 0.001 |
| 109957 | 581021 | Proteobacteria | Other Enterobacteriaceae | 91.3 | 0.0056 | 0.0054 | 0.039 |
| 117087 | 539107 | Proteobacteria | Other Enterobacteriaceae | 91.3 | 0.0041 | 0.0003 | 0.002 |
| 129367 | 3946926 | Proteobacteria | Other Enterobacteriaceae | 91.3 | 0.0023 | 0.0019 | 0.002 |
| 135326 | 148620 | Proteobacteria | Other Enterobacteriaceae | 93.0 | 0.5458 | 1.2523 | 0.001 |
| 37524 | 339660 | Proteobacteria | Other Gammaproteobacteria | 93.0 | 0.0023 | 0.0002 | 0.031 |
| 1802 | 210201 | Proteobacteria | Other Oxalobacteriaceae | 84.8 | 0.0451 | 2.8595 | 0.001 |
| 9051 | 831328 | Proteobacteria | Other Oxalobacteriaceae | 82.4 | 0.0063 | 0.0011 | < 0.001 |
| 26267 | 661838 | Proteobacteria | Other Oxalobacteriaceae | 84.1 | 0.0588 | 3.7850 | 0.001 |
| 41337 | 666883 | Proteobacteria | Other Proteobacteria | 97.8 | 0.0007 | 0.0010 | 0.005 |
| 106345 | 592425 | Proteobacteria | Other Proteobacteria | 92.8 | 2.8228 | 0.0010 | 0.030 |
| 112326 | 617287 | Proteobacteria | Other Rhodobacteraceae | 98.8 | 0.0004 | 0.0153 | 0.001 |
| 8212 | 927479 | Proteobacteria | Other Sphingomonadaceae | 96.8 | 0.5077 | 0.0004 | 0.036 |
| 83782 | 538602 | Proteobacteria | Other Vibrionaceae | 92.5 | 0.0006 | 0.0184 | 0.004 |
| 6874 | 4376318 | Proteobacteria | Paracoccus | 98.8 | 0.0908 | 8.7127 | 0.001 |
| 84159 | 245648 | Proteobacteria | Paracoccus | 97.5 | 0.0065 | 0.0007 | 0.001 |
| 6050 | 4452489 | Proteobacteria | Photobacterium | 91.6 | 0.0048 | 0.0006 | 0.006 |
| 115014 | 161024 | Proteobacteria | Photobacterium | 91.8 | 0.0009 | 0.0126 | 0.007 |
| 116227 | 4452489 | Proteobacteria | Photobacterium | 93.0 | 2.1653 | 0.4979 | 0.001 |
| 10828 | 345333 | Proteobacteria | Phyllobacterium | 97.0 | 0.0041 | 0.0010 | 0.031 |
| 131247 | 4400016 | Proteobacteria | Pseudoalteromonas | 92.7 | 0.0079 | 0.0034 | 0.023 |
| 103 | 91834 | Proteobacteria | Pseudomonas | 93.0 | 0.1428 | 0.2963 | 0.001 |
| 38639 | 4316720 | Proteobacteria | Pseudomonas | 91.1 | 0.0052 | 0.0006 | < 0.001 |
| 39283 | 1566691 | Proteobacteria | Pseudomonas | 91.1 | 0.0019 | 0.0001 | 0.036 |
| 40418 | 1566691 | Proteobacteria | Pseudomonas | 91.8 | 0.1697 | 0.0446 | 0.001 |
| 54974 | 114572 | Proteobacteria | Pseudomonas | 93.0 | 0.1060 | 0.9459 | 0.001 |
| 96752 | 4327501 | Proteobacteria | Pseudomonas | 93.0 | 0.0062 | 0.0006 | < 0.001 |
| 114231 | 280459 | Proteobacteria | Pseudomonas | 93.0 | 0.0523 | 0.2187 | 0.001 |
| 66191 | 79540 | Proteobacteria | Psychrobacter | 91.6 | 0.0025 | 0.0006 | 0.031 |
| 127036 | 110450 | Proteobacteria | Psychrobacter | 92.0 | 0.0033 | 0.0224 | 0.005 |
| 113270 | 4374816 | Proteobacteria | Psychromonas | 92.3 | 0.0059 | 0.0002 | < 0.001 |
| 13545 | 241441 | Proteobacteria | Rhizobium | 98.8 | 0.6498 | 0.1052 | 0.001 |
| 65181 | 370301 | Proteobacteria | Roseomonas | 98.5 | 0.0704 | 0.0001 | 0.045 |
| 60322 | 163061 | Proteobacteria | Shewanella | 92.7 | 0.0004 | 0.0035 | 0.031 |
| 5443 | 4449608 | Proteobacteria | Sphingomonas | 98.5 | 0.0084 | 0.0004 | 0.050 |
| 6428 | 1143645 | Proteobacteria | Sphingomonas | 98.3 | 0.1561 | 0.0324 | 0.001 |
| 117837 | 824146 | Proteobacteria | Sphingomonas | 98.0 | 0.4196 | 0.0011 | 0.021 |
| 130666 | 146007 | Proteobacteria | Sphingomonas | 98.8 | 1.2201 | 0.0954 | 0.001 |
| 82227 | 801210 | Proteobacteria | Sutterella | 90.4 | 0.0038 | 0.0002 | < 0.001 |
| 5353 | 160246 | Proteobacteria | Vibrio | 93.0 | 0.0014 | 0.0076 | 0.017 |
|  |  |  |  |  |  |  |  |
| 67629 | 147311 | Tenericutes | Mycoplasma | 90.2 | 0.8879 | 17.9903 | 0.002 |
|  |  |  |  |  |  |  |  |
| 758 | 909170 | Verrucomicrobia | Luteolibacter | 92.7 | 0.0058 | 0.0001 | < 0.001 |

^1^ unique number assigned to sequences with less than 97% similarity to other sequences

^2^ number that is associated with the sequence in the aligned Greengenes database that fits the query sequence

^3^ percent identity of the query sequence to the assigned Greengenes reference sequence
